# Energy Landscape of Relaxation and Interaction of an Amino Acid, Glutamine (L), on Pristine and Au/Ag/Cu-Doped TiO_2_ Surfaces

**DOI:** 10.3390/nano13192688

**Published:** 2023-09-30

**Authors:** Dušica Jovanović, Johann Christian Schön, Dejan Zagorac, Aleksandra Zarubica, Branko Matović, Jelena Zagorac

**Affiliations:** 1 Materials Science Laboratory, Institute of Nuclear Sciences Vinča, University of Belgrade, 11000 Belgrade, Serbia; dusica.jovanovic011@gmail.com (D.J.); dzagorac@vin.bg.ac.rs (D.Z.); mato@vin.bg.ac.rs (B.M.); 2 Department of Chemistry, Faculty of Science and Mathematics, University of Niš, 18000 Niš, Serbia; zarubica2000@yahoo.com; 3 Max Planck Institute for Solid State Research, 70569 Stuttgart, Germany; 4 Center for Synthesis, Processing and Characterization of Materials for Application in the Extreme Conditions-Cextreme Lab, 11000 Belgrade, Serbia

**Keywords:** energy landscape, glutamine, TiO_2_ surface, anatase 001 and 101 surfaces, Au/Ag/Cu-doped TiO_2_, amino acid on metal-oxide surfaces, DFT, ab initio, amino acid on anatase in vacuum

## Abstract

Studying the interaction of inorganic systems with organic ones is a highly important avenue for finding new drugs and treatment methods. Tumor cells show an increased demand for amino acids due to their rapid proliferation; thus, targeting their metabolism is becoming a potential oncological therapeutic strategy. One of the inorganic materials that show antitumor properties is titanium dioxide, while its doping was found to enhance interactions with biological systems. Thus, in this study, we investigated the energy landscape of glutamine (L), an amino acid, on pristine and doped TiO_2_ surfaces. We first locally optimized 2D-slab structures of pristine and Au/Ag/Cu-doped anatase (001 and 101 surfaces) and similarly optimized a single molecule of glutamine in vacuum. Next, we placed the pre-optimized glutamine molecule in various orientations and on a variety of locations onto the relaxed substrate surfaces (in vacuum) and performed ab initio relaxations of the molecule on the substrate slabs. We employed the DFT method with a GGA-PBE functional implemented in the Quantum Espresso code. Comparisons of the optimized conformations and electronic structures of the amino acid in vacuum and on the surfaces yield useful insights into various biological processes.

## 1. Introduction

Understanding the molecular inorganic–organic interactions in biological environments is of great importance for elucidating physiological processes, the safe application of medical implants, medicaments, drug delivery systems, the efficiency of biosensor surfaces, genetic engineering, etc. [1]. Quite generally, amino acids, which constitute the building blocks of the cell membrane, peptides, proteins, and genetic material, play a major role in biological systems, while some of the tumor cells (of lung, breast, and colon cancers that are at the top of the mortality rate) show an increased demand for amino acids due to their rapid proliferation [2]. In recent years, theoretical studies have been able to support and complement the experimental work in the area of (bio)molecules on various kinds of metallic or inorganic surfaces, such as theoretical studies of the adsorption of selected amino acids on anatase [3], on ZnO [4], on Pt [5], or of the binding mechanisms to rutile [6,7], while the transport and internal synthesis pathways for cysteine, serine, and glutamine were the most interesting targets for the development of novel redox-based therapeutics [8,9].

Recent studies have shown that tumor cells have altered metabolic pathways involving glutamine which was recently identified as an alternative to glucose for fueling the tricarboxylic acid (TCA) cycle in cancer cells [9], and many tumor cells depend on extracellular glutamine for survival. While, in general, glutamine (C_5_H_10_N_2_O_3_) is a non-essential amino acid of 20 atoms and amino, amide, and carboxyl functional groups, it is commonly recognized as a conditionally essential amino acid [10] because it plays a central role in the human nitrogen, protein, and energy metabolism [11,12,13,14,15,16], and it is a major fuel source for cells. Some metabolic regulations meet the high glutamine demand of proliferating tumor cells, which supports tumor growth by facilitating energy production and building materials’ biosynthesis [11] and can regulate redox control, gene transcription, and intracellular signaling [17,18,19]. Thus, targeting glutamine metabolism is becoming a promising oncological therapeutic strategy [10]. In addition, amino acids are often used as model systems when studying the properties of proteins in different environments [20,21]. Such research can provide a description of amino acid adsorption on, e.g., the nanocrystalline surface of some TiO_2_ modification, and an analysis of the Gibbs free energy of adsorption and thermodynamic and catalytic features of the amino acids. Here, we note that TiO_2_, a photocatalytic material with many practical applications [22], is particularly interesting since it is one of the inorganic materials that show antitumor properties; for example, the photodegradation of amino acids over UV-excited TiO_2_ particles has shown some promising results [23]. The functional groups of the amino acids have an important role in photocatalytic degradation—the carboxylate group provides the formation of stable surface complexes, while the amino group can contribute to the surface complex formation by hydrogen bonding or ligand exchange mechanisms [24,25,26,27]. Specifically, it is known that the presence of –NH_2_ and –OH groups significantly enhances the toxic effects compared to –COOH groups [28]. To better understand the impact of the adsorption on protein structure, a comprehensive understanding of amino acid–nanoparticle interactions is required [29,30]. Doping TiO_2_ was found to enhance interactions with biological systems: Au/Ag/Cu-doped TiO_2_ particles showed toxic effects on cervical cancer cell lines [31] and on human colon carcinoma [32,33,34,35,36,37,38,39,40]. Adding nanoparticles of silver improves the pathophysiological effects of photocatalytic systems, and similarly, the cytotoxic activity of Cu dopant in TiO_2_ results in a high bactericidal activity even under very weak UV light [41]. The presence of these metals on the TiO_2_ surface can enhance the charge transfer and separation efficiency, leading to improved photocatalytic performance. Moreover, cell viability was observed to depend on particle concentrations, cell types, and surface chemistry [42]. Another potential application of studying glutamine–TiO_2_ interactions is the development of new photocatalytic materials, environmental remediation, and energy conversion [43]. Many studies focus on the (101) surface orientation of anatase (one of the TiO_2_ crystal modifications) [1,2,22,23,44], but the (001) orientation has lower stability and seems to be more reactive and may contribute significantly to the anatase surface chemistry in these interactions [1,2,3,22,23,45].

Studies have shown that glutamine can interact with TiO_2_ slabs through a variety of mechanisms, including hydrogen bonding, electrostatic interactions, and van der Waals forces [44,46]. Thus, modeling of molecules [47], surfaces [48], low-dimensional systems [49], and combinations thereof will be of great importance in the investigation of glutamine on TiO_2_ surfaces. Since noble metals enhance some properties of titanium dioxide, such as anticancer and antimicrobial properties, the optimized titanium dioxide structures in this study were doped with Au, Ag, and Cu cations creating large supercells, in order to support an amino acid and to mimic potential experimental set-ups. Here, the different conformations of the glutamine (L) molecule on different anatase surfaces and their interactions in a vacuum, i.e., without a solvent, were investigated by performing quantum chemical calculations. We found that depending on the orientation of the molecule when interacting with the surface and on the kind of doping present, different reactions including dissociation of hydrogen atoms and break-up of the glutamine molecule can take place.

## 2. System and Computational Details

### 2.1. System

**Figure 1:**
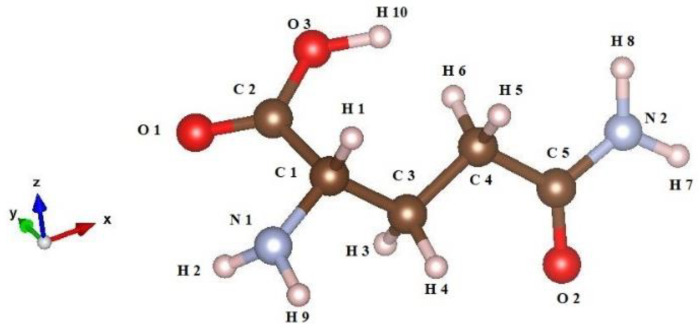
Glutamine (L) molecule, visualized by using the Vesta program [52] (atoms’ color legend: C—brown, O—red, N—light blue, H—rose).

**Figure 2:**
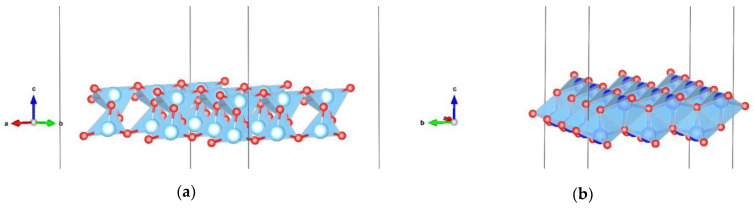
Anatase 2D slabs, visualized by using the Vesta program [52]: (**a**) 101 type of surface; (**b**) 001 type of surface (atoms’ color legend: Ti—blue, O—red).

The system being studied consists of a single glutamine molecule (Figure 1) that is placed on two different surfaces (101 and 001) of the anatase modification of TiO_2_ (Figure 2). Both pristine surfaces and surfaces doped with one metal atom (Au, Ag, and Cu) per glutamine molecule are investigated. Here, no solvent molecules were present, i.e., the glutamine–anatase system was placed in a vacuum. The initial positions of the atoms belonging to the glutamine molecule, before optimization, in a vacuum, and near the surface were adopted from the AVOGADRO program [50]. For comparison, the molecule was also optimized by itself in a vacuum using the DFT method in the generalized gradient approximation (GGA) with the Perdew–Burke–Ernzerhof (PBE) functional implemented in the Quantum Espresso code [51].

### 2.2. Computational Details

For the generation of the anatase slabs, we employed the DFT method with the exchange-correlation local-density approximation (LDA) and the Perdew–Zunger (PZ) correlation functional implemented in the CRYSTAL17 code [53], with subsequent re-optimization using the Quantum Espresso code. For the calculations involving the glutamine molecule, we used DFT (GGA plus PBE) implemented in the Quantum Espresso code [51]. Due to the low symmetry of a molecule on a slab surface, as well as the rather large number of atoms involved (54 atoms for a single slab, i.e., 18 Ti and 36 O atoms, and 74 in total for the molecule on a slab surface), the calculations were very expensive computationally. In particular, we did not perform a full global optimization of the glutamine molecule on the anatase surfaces and instead employed a simplified iterative approach.

Starting from experimental data in the ICSD database [54], we optimized the bulk anatase structure (I41/amd, Space Group 141) using the CRYSTAL17 code [53]. Next, we constructed energetically optimal slab supercells (3 × 3) of a primitive unit cell of the fully optimized bulk anatase structure, for the 001 and 101 surfaces (using the SUPERCEL, SLABCUT, and REDEFINE keywords in CRYSTAL17), and then performed a re-optimization using the Quantum Espresso code. We optimized pristine anatase slabs of both surface types (total energies are provided in Table 1) where the three top atom layers of the slabs were allowed to relax, while the remaining (bottom) layers were kept fixed. Furthermore, we doped the optimized 101 and 001 slabs by substitution of one Ti atom from the surface (Ti atom number 8 in the structure close to the center of the unit supercell, denoted as atom Ti8 in the text) by single Au, Ag, and Cu atoms, respectively (the general electronic property data of these atoms are shown in Table S1 [54]), followed by a relaxation analogous to the pristine surfaces (c.f. Table 1). Since only one dopant atom per supercell was employed, the dopant density of the substrate was very low.

Since the computational effort for full global optimization of the glutamine molecule on the surface, on the ab initio level, would have been too expensive computationally, we employed an efficient iterative approach. We first prepared the (pre-optimized) glutamine molecule in 10 different orientation configurations (labeled Orient-1 to Orient-10) by rotation about angles of 0°, 90°, and 180° with respect to the *x*-, *y*-, and *z*-axes together with choosing the location of the molecule on the surface. The atom of the molecule closest to the surface was placed at a distance of 3 Å above the slab, close to the Ti8 atom, which is later substituted by a dopant atom.

The ten glutamine + surface systems were locally optimized for both surfaces for the pristine anatase slab, where again the top three atom layers of the slab were allowed to relax. The five conformations with the lowest energies, for each surface, were selected for the next stage, where we substituted one of the Ti-atoms (Ti8) of the slab’s surface with a Au, Ag, and Cu atom. Since this resulted in noticeable changes for most of the glutamine conformations, we reversed the doping by replacing the dopant atom (for all three types of dopant atoms Au, Ag, and Cu) with the original Ti atom, and we performed another local optimization of the five conformations for each of the (now again pristine, labeled as Minimum 2–4 in the following text) anatase surfaces, leading to noticeable changes compared to the original relaxation on the pristine anatase surfaces. Next, the surface was doped again, and the glutamine + doped surface system was relaxed a second time. This time, essentially the same conformations as after the first doping step were obtained, and the iterative procedure was converged.

Finally, we computed the electron density distribution for the optimized systems and calculated the gain energies, i.e., energies gained, for all interactions and the interaction energies for systems where the molecule kept all atoms bonded, by performing additional single-point energy calculations of the optimized molecules on the surface after removing the slab, and of the optimized surfaces after removing the molecule and subtracting them from the total energy of the molecule + slab system. The energy gain (or “gain energy”) equals E_gain_ = E_Gln+slab_ − E_molrelvac_ − E_slabrel_ for all systems regardless of whether the molecule suffers a break-up, loses H atoms, or remains unbroken. The interaction energies for unbroken molecules and molecules without H atom separation were calculated as follows: E_int_ = E_Gln+slab_ − E_Gln-sp_ − E_slab-sp_ and comparing them with the relaxed molecule in a vacuum and the relaxed slabs without the molecule. Here, we use the following abbreviations: E_Gln+slab_ = total energy of system glutamine on a slab; E_molrelvac_ = energy of the molecule relaxed in a vacuum; E_slabrel_ = slab - relaxed without the molecule; E_int_ = interaction energy; E_Gln-sp_ = single-point energy of optimized/deformed glutamine without slab present; E_slab-sp_ = single-point energy of optimized/deformed slab without molecule present. All relevant Figures and Tables have been provided in the Supplementary Materials as Figure S1–S90 and Tables S1–S21.

## 3. Results

Table 2 shows the results of the first local optimization of the ten differently oriented glutamine conformations Orient-1 to Orient-10 on the pristine anatase surfaces; the corresponding structures are shown in Figures S5–S24 in the Supplementary Material (visualized by using the Vesta program [52]). We noticed significant spontaneous physical interactions between the amino acid and the slab for systems Orient-4 on the 001 pristine anatase surface and Orient-4 on the 101 pristine anatase surface, supporting the fact that the conformation Orient-4 has the lowest total energies of the system, for both types of surfaces, at this initial stage of the iterative approach.

As described in the computational details section, for further investigations of the energy landscape and the properties of the glutamine molecule on the pristine and doped anatase surfaces, we chose, for each surface (001 and 101), the conformations with the five lowest energies—mimicking five different energetically favorable ways the molecule might approach the slab. Besides Orient-4, the lowest energies were obtained for Orient-1, -3, -6, and -7 on the 001 surface and for Orient-3, -6, -7, and -9 on the 101 surface. Employing the iterative procedure described above, we obtained modified conformations of lower energy for the five orientations of the glutamine molecule with respect to the 001 and 101 surfaces, both for the doped and undoped slabs. Tables S2–S5 present the total energies of the five conformations on pristine (Minimum 1–4) 001 and 101 surfaces. We note that the ranking in the energy of the five conformations on the undoped slab changed from the initial deposition on the pristine slab (c.f. Table 1) and that the ranking of the final conformations can be different depending on the type of doping of the slab (pristine, Au-, Ag-, and Cu-dopant atom). Furthermore, we note that for some of the orientations, the contact with the doped slab resulted in various levels of breaking-up of the molecule, i.e., the removal of atoms/functional groups from the glutamine molecule, where we often distinguish between the separation of just a hydrogen atom or the separation of a whole group of atoms. Overall, we noted three different types of molecule adsorption on the surfaces—the physical interaction (only weak interaction between the molecule and a slab, without big deformation), chemical interaction (chemical bonding between the molecule and a slab, with significant deformations), and breaking-up of a molecule/separation of H atoms from a molecule (very long H-C/O distances), with noticeable deformations. The structural changes of these conformations, the interaction between the atoms in the molecule and the surface, and the possible break-ups/H dissociations of the molecule are discussed in more detail in the following subsections.

Table 3 and Table 4 present the lowest total energies of the final conformations on the two anatase surfaces for pristine and doped slabs after the iterative procedure, and the corresponding gain energies and interaction energies for unbroken and broken molecules (on 001 surfaces, Table 3) and for the molecules without the separation of H atoms (called “non-separated” case) and with separated H atoms (called “H separated” or “separated H” case) (on 101 surfaces, Table 4). Table 5 presents the lowest interaction energies of unbroken molecules without H separation and corresponding orientations with the type of interaction (while Tables S7, S9, S13 and S15 present all interaction energies for intact molecules on 001 and 101 pristine and Au/Ag/Cu-doped surfaces). Since the broken molecules were not suitable for the calculation of interaction energies that could be compared with the interaction energies of unbroken structures, we calculated gain energies for all systems obtained by using an iterative method of doping/undoping and compared them (Tables S6, S8, S12 and S14 present the gain energies of all unbroken and broken/H separated molecule conformations).

The lowest total energies for the unbroken molecules on 001 surfaces were found for the Orient-1 configuration on pristine (Minimum 2 conformation), Ag-doped, and Cu-doped slab surfaces and Orient-4 on Au-doped surfaces, while the broken systems showed the lowest total energies for Orient-7 Minimum 4 on the pristine 001 slab surface, Orient-3 on the Au-doped, and Orient-4 on the Ag-doped and Cu-doped 001 slab surfaces. The lowest total energies for the case of non-separated H atoms on 101 surfaces were obtained for Orient-3 on pristine Minimum 4/Ag/Cu-doped slabs and Orient-9 on the Au-doped surface, while the systems with separated H atoms showed the lowest total energies for Orient-3 on pristine Minimum 3, Orient-6 on Au/Ag-doped surfaces, and Orient-9 on the Cu-doped 101 surface. The biggest energy gain was seen for the unbroken Orient-1 Cu-doped system and a broken Orient-4 Ag-doped system on the 001 surfaces and the Orient-3 Ag-doped with non-separated H atoms system and the Orient-6 Au-doped system with H atom separation on 101 surfaces. The lowest interaction energies correspond to the structures with the lowest total energies—Orient-1 Minimum 2 on the pristine 001 surface (chemical interaction) and Orient-3 on the Ag-doped 101 surface (physical interaction).

### 3.1. Glutamine (L) Molecule on Pristine and Doped 001 Anatase Surface Interactions

Upon placing the molecule on the (001) anatase slab surface, we observed interactions between the molecule and surface, both for the pristine and the three doped surfaces (Au-, Ag-, and Cu-doped), which resulted in deformations of both the molecule and the slab with newly formed bonds. Depending on the degree of deformation and on the formation of bonds to the surface or break-up of the molecule, we speak of physical interactions, chemical interactions, and the breaking of a molecule. If the break-up was observed for a given orientation of the molecule on the surface, then the energy of the broken molecule on the surface was usually lower than the best minimum we found for the unbroken molecule on the surface for this orientation. In order to demonstrate the wide range of possible minimum configurations encountered for the glutamine molecule on the pristine surface, for a given orientation, in Figure S1, we show four such minima (columns are labeled Minimum 1 to Minimum 4 in the figure) for the pristine surface for each of the five selected orientations. 

On the pristine anatase 001 surfaces, the lowest total energy and the interaction energy of the molecule were found for the Orient-1 Minimum 2 configuration (Table 3 and Table 5), with chemical interaction and physical deformation of both the molecule and surface, while the Orient-7 Minimum 4 exhibited the lowest total energy for the broken molecule case. As can be seen in Figure S1, physical interactions with the pristine surface were exhibited by Orient-1—Minimum 1, Orient-3—Minimum 1, 2, and 3, Orient-6—Minimum 1 and 2, and Orient-7—Minimum 1, 2, and 3. Orient-1—Minimum 2, 3, and 4, Orient-4—Minimum 1 and 2, and Orient-6—Minimum 4 showed chemical interactions, while the molecule was broken for Orient-3—Minimum 4, Orient-4—Minimum 3 and 4, Orient-6—Minimum 3, and Orient-7—Minimum 4. All these examples also involved a physical deformation of the molecule and the slab (Tables S2 and S18).

**Figure 3:**
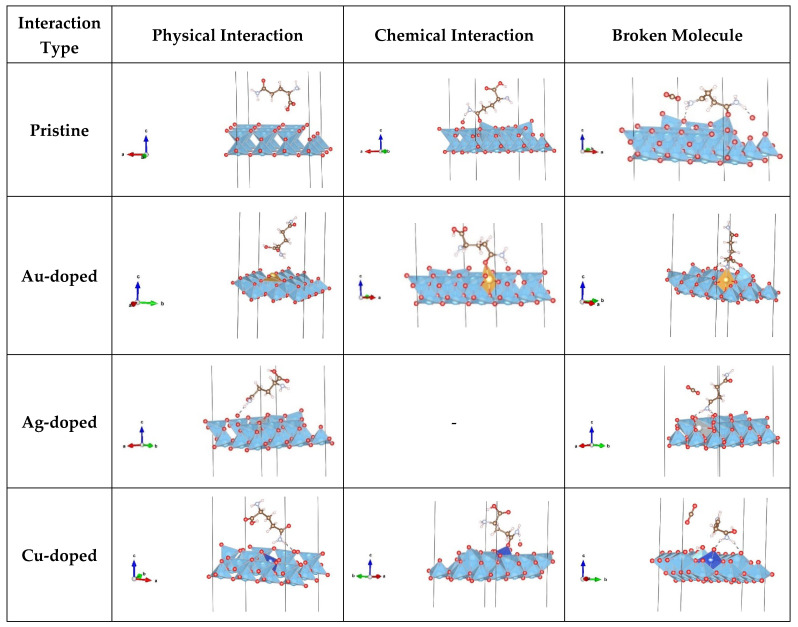
Visualized various glutamine (L) molecule conformations (Orient-1, -3, -4, -6, and -7) on pristine and Au/Ag/Cu-doped 001 anatase surfaces that exhibited a physical/chemical interaction and/or a breaking of the molecule. Pristine—Orient-7 (physical), Orient-1 (chemical), Orient-4 (breaking); Au-doped: Orient-6 (physical), Orient-4 (chemical), Orient-3 (breaking); Ag-doped: Orient-1 (physical), Orient-6 (breaking); Cu-doped: Orient-7 (physical), Orient-1 (chemical), Orient-4 (breaking).

**Figure 4:**
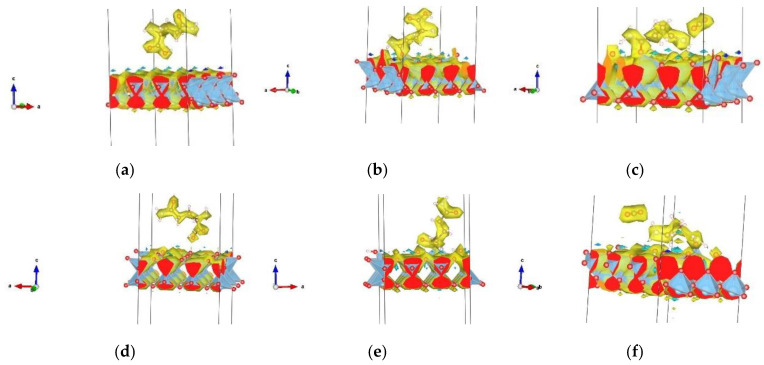
Examples of electron densities: (**a**) physical interaction of Minimum 1 conformation of Orient-7 on pristine anatase 001 surface; (**b**) chemical interaction of Minimum 2 conformation of Orient-1 on pristine anatase 001 surface; (**c**) molecule’s breaking-up of Minimum 3 conformation of Orient-4 on pristine 001 anatase surface; (**d**) physical interaction of Orient-7 on Au-doped anatase 001 surface; (**e**) chemical interaction with the breaking of the molecule—H atom separated and bonded with the surface, of Orient-3 on Cu-doped anatase 001 surface; (**f**) molecule’s breaking-up of Orient-4 on Ag-doped 001 anatase surface.

On the three doped (Au, Ag, Cu) (001) anatase surfaces, the lowest total energies (Table 4) were found for Orient-4 on the Au-doped surface, with a chemical interaction, Orient-1 on the Ag-doped surface with physical interaction, and Orient-1 on the Cu-doped surface with a chemical interaction, while the lowest total energies found for a broken molecule appeared for the Orient-3 on the Au-doped surface with the separation of one H atom bonded to oxygen from the surface, Orient-4 on the Ag-doped surface, with the separation of the O-C-O chain and chemical interaction, and Orient-4 on the Cu-doped surface with the separation of the O-C-O chain and physical interaction. Physical interactions were also observed for Orient-6 and Orient-7 on the Au-doped surfaces, Orient-1 and Orient-7 on the Ag-doped surfaces, and Orient-6 and Orient-7 on the Cu-doped surfaces (Figure S2, Table S18). Chemical interactions were observed for Orient-1 on the Au-doped and Cu-doped surfaces, while Orient-3 and Orient-4 both on the Ag-doped and Cu-doped surfaces showed chemical interactions together with the molecule’s break-up. The break-up of the molecule occurred for Orient-3 on all three doped surfaces, for Orient-4 on the Ag- and Cu-doped surfaces, and for Orient-6 on the Ag-doped surfaces. Some of the most representative structures for each occurred interaction with pristine and doped 001 surfaces are presented in Figure 3. For several characteristic instances, Figure 4 depicts the electron density distribution of the molecule (or its fragments) on the anatase (001) surface; additional pictures can be found in the Supplementary Material (Figures S84, S87 and S88).

Analyzing these interactions of the various local minimum configurations of the glutamine molecule with the anatase (001) surface in more detail, we find that the Orient-1 conformation showed physical interactions between an amide group (-CONH_2_) and both the pristine and the doped surfaces. Regarding Orient-3, we observe interactions and breaking of the amino group (-NH_2_) for the Minimum configurations 2 and 3 on the pristine surface, while both amino and carboxyl (-COOH) groups were involved in breaking and interactions with the pristine surface for Minimum 4 and with all three doped surfaces. Next, in the case of Orient-4 there are interactions between the amide group and the pristine surface for Minimum 1 and 2, and with the Au-doped surface; stronger interactions of the amide group accompanied by breaking and the separation of the plain, i.e., without hydrogen atoms, chain (O1-C2-O3) of the carboxyl group occurred for Minimum 3 and 4 on the pristine surface and on the Ag- and Cu-doped surfaces. Regarding Orient-6 we observed a similar breaking and separation of the (O1-C2-O3) chain of the carboxyl group during interaction for Minimum 3 on the pristine surface and for the lowest-energy configuration on the Ag-doped surface, while the interaction of Minimum 4 on the pristine surface involved binding with the amide group; finally, the interaction of Orient-6 with the Cu-doped surface occurred via the amino group. Lastly, Orient-7 exhibited both interaction and breaking of the amide group on the pristine surface for Minimum 4 and an interaction between the amide group and the Cu-doped surface. The interatomic distances for all of these interactions are presented in Tables S10 and S11, while all types of interactions for each conformation are presented in Table S18.

### 3.2. Glutamine (L) Molecule on Pristine and Doped 101 Anatase Surface Interactions

**Figure 5:**
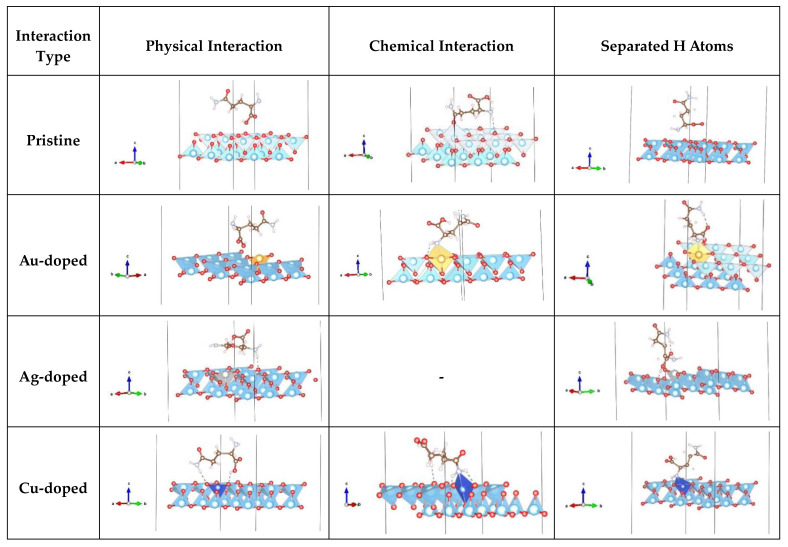
Visualizations of various glutamine (L) molecule conformations (Orient-3, -4, -6, -7, and -9) on pristine and Au/Ag/Cu-doped 101 anatase surfaces that exhibited a physical/chemical interaction with the surface and/or saw a breaking of the molecule. Pristine: Orient-7 (physical), Orient-4 (chemical), Orient-3 (breaking); Au-doped: Orient-7 (physical), Orient-9 (chemical), Orient-6 (breaking); Ag-doped: Orient-4 (physical), Orient-6 (breaking)—no example of a purely chemical interaction was observed for this surface type; Cu-doped: Orient-7 (physical), Orient-4, (chemical), Orient-9 (breaking). Note that a break-up of the molecule frequently included a chemical interaction of the remnant with the surface.

An analogous analysis of the deposition of the glutamine molecule on the pristine and doped anatase (101) surfaces was performed. Again, we show results for four minimum configurations for the deposition on the pristine (101) surface and the lowest-energy configurations for the Au-, Ag-, and Cu-doped surfaces, for five orientations that had been selected after a first round of local minimizations. On the pristine surface, the Orient-3 Minimum 4 conformation was the lowest one in total energy, energy gain, and interaction energy and showed physical interaction, for unbroken/non-separated H atoms of the molecule (Table 4 and Table 5), while for the structures where H atoms had separated from the molecule, the lowest total energy was obtained for Orient-3 Minimum 3 on the pristine surface. As can be seen in Figure 5, physical interactions with the pristine surface were exhibited by Orient-3—Minimum 4, Orient-4—Minimum 4, and Orient-7—Minimum 1, 2, 3, and 4. Orient-9—Minimum 1, 2, 3, and 4 and Orient-4—Minimum 1, 2, and 3 showed a chemical interaction, while for Orient-3—Minimum 1, 2, and 3 and Orient-6—Minimum 1, 2, 3, and 4, a separation of H atoms took place (Table S19).

Concerning the doped surfaces, the lowest energies of glutamine on the three doped surfaces were observed with an unbroken molecule for Orient-9 on the Au-doped surface (chemical interaction) and Orient-3 on the Ag-doped (physical interaction) and Cu-doped (chemical interaction) surfaces, while for the case of configurations with separated H atoms on the Au/Ag-doped 101 surfaces, the lowest total energies were found for Orient-6 and for Cu-doped Orient-9 (Table S19). Figure S3 shows four local minimum configurations of glutamine, for each of the five selected orientations (Orient-3, -4, -6, -7, -9), on the pristine anatase (101) surface, while Figure S4 shows the energy configurations on the three doped surfaces.

**Figure 6:**
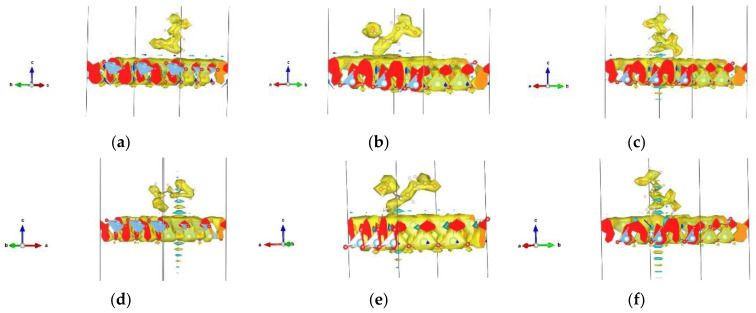
Examples of electron densities: (**a**) physical interaction with separated H atoms of configuration Orient-3 Minimum 2 on the pristine anatase 101 surface; (**b**) chemical interaction of Orient-9 Minimum 4 on the pristine anatase 101 surface; (**c**) physical interaction with H atom separation of Orient-3 Minimum 3 on the pristine 101 anatase surface; (**d**) physical interaction of Orient-7 on the Au-doped anatase 101 surface; (**e**) chemical interaction of Orient-9 on the Ag-doped anatase 101 surface; (**f**) physical interaction with H atoms separated from Orient-3 on the Au-doped 101 anatase surface. For more examples, see Figures S85, S89 and S90.

On the three doped (Au, Ag, Cu) (101) anatase surfaces, physical interactions were observed for Orient-7 on the Au-doped surface, for Orient-3, Orient-4, Orient-7, and Orient-9 on the Ag-doped surfaces, and for Orient-7 on the Cu-doped surfaces. Chemical interactions were present for Orient-3, Orient-4, and Orient-9 on the Au-doped slab, while for Orient-6, a separation of H atoms besides a chemical interaction occurred. Orient-4 showed a chemical interaction with the Cu-doped surface, while Orient-3 and Orient-9 exhibited chemical interactions and separation of H atoms. Configuration Orient-6 experienced a separation of the H atoms on all three doped surfaces, while Orient-3 showed such behavior only on the Au-doped surface, and Orient-9 on the Cu-doped surface (Table S19). For several characteristic instances, Figure 5 shows some of the most representative interactions for each type of surface. Figure 6 depicts the electron density distribution of the molecule (or its fragments) on the anatase (101) surface; additional pictures can be found in the Supplementary Material (Figures S85, S89 and S90).

Considering the interactions in more detail, we find that Orient-3 showed interactions between the carboxyl and amino groups (with hydrogen separation) and all types of dopant atoms in the 101 doped surfaces, while the same orientation conformation showed interactions between the carboxyl group and the pristine surface for Minimum 2 and 3, together with the hydrogen separation mentioned above.

For Orient-4, there is an interaction between the amide group of Minimum 3 and the pristine surface and between the amino group and the Ag-doped (101) surface; in fact, both functional groups showed interactions with Au-doped and Cu-doped surfaces and for Minimum 1 and 2 for pristine surfaces. Minimum 1, 2, and 4 of Orient-6 on the pristine surface involved interactions between the amino or the carboxyl groups and the surface while Au/Ag-doped 101 surfaces established interactions with molecules that had been deformed into a roundish shape, approaching carboxyl and amide groups, with the presence of separated hydrogen atoms in all cases except pristine and Au-undoped 101 surfaces. Orient-7 exhibited a heart-shaped deformation of the molecule when in interaction with the pristine (Minimum 4) and the Cu-doped (101) surfaces, resulting in a bond between the carboxyl group and surface for the pristine case and between the carboxyl and amide groups for the Cu-doped one. Finally, for Orient-9, we observed an interaction between the amino group and the pristine surface (for Minimum 2–4) and all three doped (101) surfaces, with direct binding to the dopant atoms in the cases of Au- and Cu-doping, combined with the aforementioned hydrogen separation during the interaction with the Cu-doped surface. The separation of H atoms in all cases was noticed in the interatomic distance range of 1.208–1.276 Å (all interatomic distances for 101 surfaces are presented in Tables S16 and S17).

### 3.3. Dominant Interactions of Glutamine (L) Molecule on Investigated Anatase Surface Systems

In the previous Tables and Figures, we showed the best energetical candidates of investigated systems, the gain and the interaction energies, and the significant interactions (physical, chemical, and break-ups/H atom separations of the molecule) together with electron density visualization (and interatomic distances of interactions in the Supporting Material).

**Figure 7:**
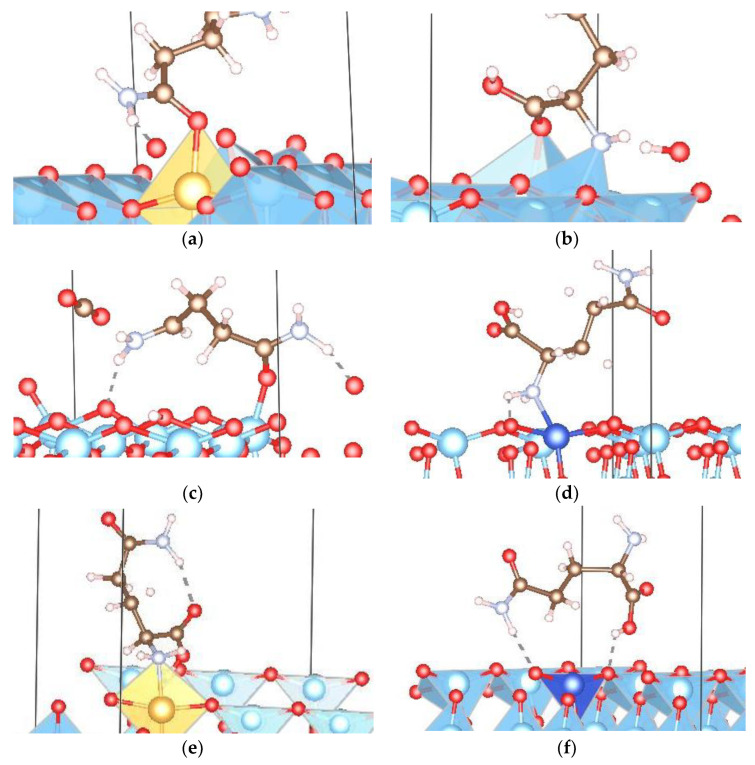
Presentation of dominant interactions in this study between (metal atoms in) the various anatase slab surfaces (configuration given in brackets) and (**a**) amide group (Orient-1—Au-doped 001 anatase surface); (**b**) carboxyl and amino groups (Orient-1—Minimum 4 001 anatase surface); (**c**) amino, amide, and carboxyl groups, with specific denaturation of carboxyl group—linear chain (Orient-4—Minimum 3 001 anatase surface); (**d**) direct interaction with dopant atom and separation of hydrogen atoms of the molecule (Orient-9—Cu-doped 101 anatase surface); (**e**) round-like shape deformation of molecule and separation of hydrogen atoms (Orient-6—Au-doped 101 anatase surface); and (**f**) heart-shaped deformation of the molecule, with carboxyl and amide group interactions (Orient-7—Cu-doped 101 anatase surface).

We could distinguish between three types of interactions in this study: physical interactions, chemical interactions with deformations of molecule and surface, and breaking of molecules/separation of H atoms, sometimes accompanied by physical or chemical interactions between the molecular fragments and the surface. If chemical interactions with deformations were present, this made itself felt by the appearance of various connections—bonds between surface and molecule atoms, unusually short atom–atom distances, and bonds between atoms in the surface and the amino, amide, or carboxyl functional groups of the molecule. According to the results presented and the interactions discussed in the previous subsections, we note that under the initial conditions (without the employment of an iterative method of searching for a better minimum), only the Orient-4 amino acid conformation showed spontaneous physical interactions with all anatase surfaces—pristine and doped (001) and (101) slab surfaces. Many of the orientation conformations on the pristine and doped (001) anatase surfaces showed physical/chemical interactions, or chemical interactions together with the breaking of the molecule (the corresponding systems are presented in Figures S1 and S2, Table S18 in the Supplementary Materials) with the surface/dopant atom. Similarly, various orientation conformations of the molecule on pristine (101) anatase surfaces after optimization frequently exhibited separated H atoms, physical interactions, or physical interactions in conjunction with separated H atoms, while various orientations of glutamine on doped (101) surfaces showed a chemical interaction between molecule and slab/dopant atoms or such an interaction together with dissociation of H atoms from the molecule (Figures S3 and S4, Table S19). Some of the most interesting deformations and dominant interactions are presented in Figure 7.

The ability of the investigated material, anatase, to interact with the glutamine molecule, proved to be enhanced by Au/Ag/Cu doping for both types of surfaces since this induced stronger interactions. In particular, the (001) surfaces showed quite strong interactions—typically stronger than the corresponding (101) surface slab, often resulting in a break-up of the molecule, while the (101) surfaces showed moderately strong physical interactions, typically leading at most to the loss of a hydrogen atom, for both pristine and doped surfaces. In addition, the doped regions of both the (001) and (101) slab surfaces exhibited direct chemical interactions between the Au/Cu-dopant atoms and the molecule, while the (001) Ag-doped anatase surface showed O–H bonding, together with the breaking of the molecule. In comparison with other dopants, Cu-doped (001) surfaces were most likely to induce a break-up of the glutamine molecule. For both the (101) and (001) surfaces, the Ag dopant induced physical interactions, but the 001 surface case also exhibited a chemical interaction with the breaking of the molecule. In contrast, in the case of the 101 surface, at most, a separation of the H atoms, without subsequent chemical bonding, occurred. The lowest interaction energy for non-broken molecule systems was calculated for the pristine 001 slab surface that exhibited chemical interaction and the Ag-doped 101 slab surface that exhibited physical interaction.

Here, we also note that, depending on the minimum configuration of the molecule on the surface for a given orientation conformation, the strength of the interaction could vary, such that the molecule remained whole in one instance and became broken in another. While this kind of control would be very desirable, it is unlikely to be achieved in a simple manner; nevertheless, this underlines the richness and subtlety of the complex energy landscape of the glutamine-on-anatase surface system. In this way, anatase could be used for the purpose of achieving physical interactions with glutamine, for the adsorption of glutamine, for chemical interaction, and for the molecule’s breaking, which can be adjusted by using a corresponding dopant atom and its position and concentration. Experimental application, depending on its goal, can be performed by manipulating the orientation of molecules and adjusting the composition (by doping with a selected dopant or not) and properties of the material, according to the envisioned application.

Finally, regarding the methodology we have employed, we note that performing the limited global search via a sequence of local minima at the ab initio level by the implementation of an iterative method led to noticeable improvements in the energy of the local minimum both for the pristine model and doped system interactions, compared to the standard way of just slowly letting the molecule settle on the surface for a number of orientations. While a full global optimization on the ab initio level of the molecule on the surface would have been desirable, this approach of dopant-substitution and re-substitution cycles in the substrate surface allowed us to greatly reduce the computational effort while still achieving substantial improvements in the energy and variety of the local minimum configurations of the glutamine–anatase system and might prove to be a useful tool for future theoretical investigations of similar inorganic–organic systems.

## 4. Summary, Discussion, and Conclusion

This study presents a computational investigation of the energy landscape, on the ab initio level, of a molecule + substrate system—glutamine on pristine and doped TiO_2_, in a vacuum. We discussed possible inorganic–organic interactions of the systems depending on the properties of the investigated TiO_2_ material, which may be applicable as a potential anticancer agent and/or nanoscale therapeutics. Here, glutamine was chosen as an example system because of its important role in cancer metabolism: it has been identified as an alternative to glucose for fueling the tricarboxylic acid (TCA) cycle in cancer cells, and many tumor cells depend on extracellular glutamine for survival.

We prepared systems of various glutamine conformations on quasi-2D-slab surfaces, with 001 and 101 orientations, of pristine and Au/Ag/Cu-doped anatase, in order to mimic the interactions of nanoparticles and an amino acid molecule. The ab initio calculations were performed on the DFT level with the LDA and GGA-PBE functionals, implemented in two different codes—CRYSTAL17 and Quantum Espresso. Due to the low symmetry of systems and the large number of atoms involved, these calculations were computationally very expensive and highly memory-demanding. Thus, we employed a new iterative method of searching for low-energy minima for molecule-on-ceramic-type surface systems by alternating between doped and undoped surfaces, which proved to be very efficient for this kind of inorganic–organic systems.

Clearly, we study a simplified model because we do not include a real medium but let the molecule approach the surface in a vacuum, and, furthermore, we do not include thermal energy in the system as one would do for a molecular dynamics simulation. Instead, we reduce the initial energy of the combined molecule + substrate system while we are moving the molecule downhill into the minima via gradient descent on the energy landscape of the system. During this relaxation, the combined system reduces its total energy by (a) moving the molecule toward the surface, (b) changing the shape of the molecule, (c) slightly deforming the surface, and, in certain cases, (d) breaking up the molecule. Note that while we are moving the molecule in an infinitesimal fashion toward the surface during the gradient minimization, and we essentially extract all the “kinetic” energy all the time, we do not force the molecule to stay intact during this process until it has settled into a minimum configuration on the surface. Thus, the system moves into one of the many basins of the landscape of the molecule + substrate system, starting at a higher energy than the final minimum configuration. In particular, this energy can be high enough to allow the system to pass through/move past a number of saddle points on its downhill path, and, depending on the starting configuration, we can either reach a local minimum where the molecule has remained intact or one where the molecule has been broken into two pieces. In particular, this means that in certain situations, the energy released is large enough to overcome the energetic barriers that would have prevented the break-up of the isolated molecule located on the surface. Thus, there can be enough energy in the system—in some way equivalent to a spatially (and temporally) localized sufficiently high finite temperature—such that some downhill paths will result in a break-up.

This simplified model helps us study an important limiting case—the approach of the glutamine molecule to the surface without any noticeable hindrance by other molecules surrounding the TiO_2_ nanoparticle. We note that going beyond this model system, we would either have to make other strong assumptions about the medium, or we would need to perform ab initio or QM/MM molecular dynamics calculations of the glutamine + TiO_2_ system within a realistic medium, which would go beyond the purview of this study.

Using this approach, a number of valuable insights into possible molecule–surface interactions of the investigated systems could be obtained. The investigated material, anatase, proved to exhibit enhanced interactions with the glutamine molecule after Au/Ag/Cu doping compared to the pristine surface. Furthermore, by selecting different types of doping of the anatase substrate and varying the initial orientation of the molecule, this nanocrystalline material can be used for fine tuning the physical and chemical interactions with the glutamine molecule or inducing a break-up of or a dissociation of H atoms from the molecule. In particular, the interactions depend on the dopant atom selection, the relative positions of the dopant atom and the molecule, the dopant’s concentration, the relative orientation of the molecule with respect to the slab surface orientation, the type of surface orientation, and the distance between the molecule and surface. The 001 anatase surfaces showed stronger interactions than the 101 surfaces, in agreement with the earlier investigations [1,2,3,19,20,42]. One of the minimum conformations of glutamine on the undoped 001 surface showed the lowest interaction energy for the whole set of molecule configurations explored, together with direct bonds between the Au/Cu dopant atoms and the molecule, while the Ag-doped 001 surfaces preferred adsorption by O–H bonding accompanied by a breaking of the molecule and showed the lowest interaction energy among the broken molecule configurations. Cu-doped 001 surfaces mostly tend to break the molecule and show the biggest energy gain, while Cu-doped 101 surfaces induce the separation of hydrogen atoms. In general, 101 surfaces showed strong physical interactions, typically leading only to the loss of a hydrogen atom with the separation range C–H of 1.208–1.278 Å, for both pristine and doped surfaces. The Au-doped 101 anatase surface with the molecule’s H atom separation showed the biggest energy gain, while the Ag-doped 101 slab surface exhibited mostly physical interaction and showed the strongest interaction energy for non-separated H atom structures and the biggest energy gain. Based on the potential application envisioned, manipulation of the molecule’s and material’s relative orientations and positions could produce promising results in practice.

While it would also be interesting to study ways to dope TiO_2_ surfaces in the experiment and theoretically analyze the kinetic stability of such—possibly thermodynamically metastable—substrates (without the addition of glutamine, which takes place in the second step which is the one analyzed in our study), this is not in the purview of this study where we assume the availability of such a surface; note that we are in the low-doping limit (only one Ti atom has been substituted by a dopant atom). Here, we focused on the glutamine-(doped/undoped) substrate interactions, and we did not investigate the issues associated with the doping of anatase surfaces and their general properties and experimental realizability—for discussions of the effect of low doping concentration on the electronic/catalytic properties of TiO_2_, in experimental and computational studies (DFT), we refer to the literature [55,56,57,58,59,60].

The most important result of this study might be that even without exposing the system to high temperatures or irradiation by high-energy photons, we find that just the simple adsorption process of glutamine on TiO_2_ (for pure and, even more strongly, for doped) surfaces can locally release enough energy to lead to a break-up of the molecule. We performed (c.f. Tables S20 and S21) a conversion of the break-up energies (the difference between the final local minimum energy and the single-point energy of the starting configuration of the system) into equivalent local temperatures (K), where for simplicity, we assumed that the energy released is equally distributed over the atoms of the molecule. In this fashion, we can associate this energy with a local temperature of the molecule that would be available for the break-up if the system were in thermal equilibrium at that temperature—at least for a short time until this excess energy has dissipated throughout the whole nanoparticle and surrounding medium; we note that this short time-window for such a break-up reaction to take place corresponds to an entropic contribution to the free energy barrier of reaction [61], analogous to the attempt-frequency pre-factor in transition state theory [62,63]. Furthermore, since we can, in principle, move from an unbroken local minimum configuration of the molecule on the surface back to the initial configuration and then on toward a minimum configuration that corresponds to a broken molecule on the surface, the two corresponding energy differences between the initial configuration and the two final local minimum configurations yield upper limits on the energy barriers between the two local minima on the energy landscape. More precise estimates of such transition barriers could be obtained using various transition barrier sampling techniques such as transition path sampling [64], nudged elastic band methods [65], or the threshold algorithm [66].

We note that these equivalent local temperatures are quite high and thus should be more than sufficient for crossing the potential energy barriers of the system. According to data by Rodante et al. [67] on the thermal decomposition of glutamine, the activation energy and the temperature range obtained by using different methods, such as thermogravimetry, were as follows: E_a_ = 5.9 kcal/mol and T = 173–481 °C. Clearly, the local temperatures associated with the energy released in our calculations are much higher, even if we assume that the local substrate atoms adjacent to the glutamine molecule acquire half of the energy released (thus halving the equivalent local temperatures). Of course, in practice—i.e., if we consider the environment of interest, e.g., the human body—one would have to replace the vacuum of our calculations with the appropriate medium (fluids, tissue, etc.) surrounding the glutamine molecule and the TiO_2_ nanoparticle, which would further reduce the equivalent local temperature we obtained for the vacuum case. Nevertheless, the final objective—the destruction of the glutamine molecule by the catalytic activity of the nanoparticle—should be achievable.

On the other hand, one might be concerned about the possible negative side effects of such a break-up reaction and the relatively high equivalent local temperatures that may appear in the process. Here, one needs to keep two aspects separate: the equivalent local temperature on the nanometer scale, which controls the catalytic reaction we investigate but is present only for a short time, and the (equilibrium) temperature in the human body on the micro/millimeter scale of cells and tissue. Unless one were to flood the body with TiO_2_ particles, the effect of these local reactions should not lead to a great rise in temperature, since the fluid and tissue surrounding the reacting entities will absorb much of the energy and thus dilute the heat generated. In this context, we note that the decomposition products of glutamine on 001 anatase surfaces with O-C-O separated chains appeared in two different molecular structures (C_4_H_7_N_2_O (Orient-4—Minimum 4) and C_4_H_9_N_2_O (Orient-4—Minimum 3, Orient-6—Minimum 3, Orient-4—Ag/Cu-doped, and Orient-6—Ag-doped)) without any rings; in some cases, they are chemically bonded to the TiO_2_ surface via oxygen. However, neither of them corresponds to a compound with known potential influence on human health.

Of course, this effect of the environment surrounding the glutamine + TiO_2_ system—both within the human body and in more general situations—and, conversely, its response to a large local energy release (including possible medical side effects such as the damage to important biomolecules) needs to be investigated in more detail, both experimentally and theoretically. Regarding such future theoretical work, it will be natural to first consider the case of adding a fluid medium, as mentioned above, in which the movement of the glutamine molecule to the surface takes place.

In this respect, our study presents a “first insights” type of investigation, where we explore the general feasibility of using doped and undoped TiO_2_ for achieving such break-ups in the first place, by investigating a simplified model system. We feel that the large variety of minimum configurations of glutamine on the surface (both broken and unbroken ones) presented here, and their detailed analysis, could greatly contribute to our understanding of the interactions between glutamine (in this case, in vacuum) and the TiO_2_ surface (the potential anticancer material). Furthermore, our results serve as a strong indication that such glutamine–TiO_2_ interactions should be important, either in a helpful way by breaking up dangerous molecules or in an adversarial one by destroying valuable biomolecules, and should be investigated further. Quite generally, our study shows that the application of well-prepared anatase nanoparticles, where some parts of the surfaces are Au/Ag/Cu-doped and others are undoped, should allow us to control the rate of adsorption/break-up processes of amino acids. The information obtained in this work regarding potential interactions between glutamine and pristine/doped anatase could contribute to designing new or enhanced medicaments and therapeutic systems at the nanoscale. Furthermore, our results support the expectation that TiO_2_ nanoparticles can be applied as an anticancer or antimicrobial agent, as suggested in the past. 

More generally, amino acids as the building blocks of proteins, cell membranes, and various organic species can be affected by such nanoparticles with a variety of purposes in the mind of the experimentalist or medical practitioner. Here, we note that the methods presented in this study should be directly transferable to investigations of analogous organic molecules on ceramic surfaces. This indicates that the results presented can contribute to general investigations of biomolecules on metal-oxide surfaces, as well as to the potential design of new anticancer materials, drug delivery systems, and nanoscale therapeutics mentioned above. Going beyond the field of medicine, the insights gained in this study should also be relevant to the investigation of environmental remediation, catalytic and corrosion resistance materials, energy conversion, and green energy production [2,3,4,5,6,7,8,9,12,23,28,29,30,31,32,33,34,35,36,37,38,43,68,69,70,71,72,73].

## Figures and Tables

**Table 1:** The energies of the optimized single glutamine (L) molecule in a vacuum and pristine and doped anatase slabs (3 × 3 supercell) for 001 and 101 types of surfaces, with the top 3 atom layers of the slab allowed to relax but keeping the rest of the slab fixed. The energies were computed with the Quantum Espresso code (DFT-GGA + PBE).

System	Energy (Ry)
Glutamine	−206.0196618
Pristine anatase slab 001 surface	−3297.6523021
Pristine anatase slab 101 surface	−3296.4745584
Au-doped anatase slab 001 surface	−3293.4406901
Au-doped anatase slab 101 surface	−3292.3081166
Ag-doped anatase slab 001 surface	−3474.5630748
Ag-doped anatase slab 101 surface	−3473.4073269
Cu-doped anatase slab 001 surface	−3580.1960144
Cu-doped anatase slab 101 surface	−3579.0250252

**Table 2:** Total energies (Ry) of optimized systems of various conformations of glutamine (L) molecule (orientations 1–10) on pristine anatase slab surfaces 001 and 101 types, after the first local optimization.

System	001	101
**Orient-1**	−3503.9665405	−3502.7640868
**Orient-2**	−3503.9520073	−3502.7636141
**Orient-3**	−3504.0123309	−3502.8028739
**Orient-4**	−3504.0150932	−3502.8226043
**Orient-5**	−3503.9572802	−3502.7618474
**Orient-6**	−3503.9622164	−3502.7801019
**Orient-7**	−3503.9631739	−3502.7783099
**Orient-8**	−3503.9596757	−3502.7595644
**Orient-9**	−3503.9516392	−3502.7952086
**Orient-10**	−3503.9563369	−3502.7635405

The lowest total energies are marked in yellow.

**Table 3:** The lowest total energies (Ry) of systems of the various glutamine (L) molecule conformations (Orient-1, -3, -4, -6, and -7) on pristine and Au/Ag/Cu-doped anatase 001 surfaces, corresponding gain energies for the cases of broken and unbroken molecules, and the interaction energies for unbroken molecules.

001	Unbroken				Broken		
System	Orient	Total Energy (Ry)	Energy Gain (Ry)	Interaction Energy (Ry)	Orient	Total Energy (Ry)	Energy Gain (Ry)
Pristine	1	−3504.0640618	−0.3920979	−0.1950718	7	−3504.0509188	−0.3789549
Au-doped	4	−3499.8401899	−0.3798380	−0.1928299	3	−3499.9391179	−0.4787660
Ag-doped	1	−3680.9600300	−0.3772934	−0.0859600	4	−3681.2178991	−0.6351625
Cu-doped	1	−3786.6187830	−0.4031068	−0.0952430	4	−3786.8429452	−0.6272690

The values marked in yellow correspond to the lowest energies.

**Table 4:** The lowest total energies (Ry) of systems of the various glutamine (L) molecule conformations (Orient-3, -4, -6, -7, and -9) on pristine and Au/Ag/Cu-doped anatase 101 surfaces, corresponding energy gain for the cases of molecules with and without separated H atoms, and the interaction energies for the cases without separated H atoms.

101	Non-Separated H Atoms				Separated H Atoms		
System	Orient	Total Energy (Ry)	Energy Gain (Ry)	Interaction Energy (Ry)	Orient	Total Energy (Ry)	Energy Gain (Ry)
Pristine	3	−3502.8244786	−0.3302584	−0.0750086	3	−3502.8242836	−0.3300634
Au-doped	9	−3498.7046715	−0.3768931	−0.1227615	6	−3498.7446577	−0.4168793
Ag-doped	3	−3679.8244536	−0.3974649	−0.1566136	6	−3679.8171251	−0.3901364
Cu-doped	3	−3785.4294650	−0.3847780	−0.1506750	9	−3785.3841755	−0.3394885

The values marked in yellow correspond to the lowest energies.

**Table 5:** The lowest interaction energies (Ry) for intact structures of the glutamine molecule on pristine and Au/Ag/Cu-doped 001 and 101 anatase slab surfaces for the unbroken molecules with the corresponding types of interaction.

Unbroken	001			101		
System	Orient	Interaction Energy (Ry)	Type of Interaction	Orient	Interaction Energy (Ry)	Type of Interaction
Pristine	1	−0.1950718	chemical	3	−0.0750086	physical
Au-doped	4	−0.1928299	chemical	9	−0.1227615	chemical
Ag-doped	1	−0.0859600	physical	3	−0.1566136	physical
Cu-doped	1	−0.0952430	chemical	3	−0.1506750	chemical

The values marked in yellow correspond to the lowest energies.

## Data Availability

Not applicable.

## Supplementary Materials

The following supporting information can be downloaded at https://www.mdpi.com/article/10.3390/nano13192688/s1, Table S1: An overview of the electronic features of dopant atoms used in this study, Tables S2 and S3: Total energies (Ry) of systems of various glutamine (L) molecule conformations on 001 pristine and Au/Ag/Cu-doped anatase surfaces, Tables S4 and S5: Total energies (Ry) of systems of various glutamine (L) molecule conformations on 101 pristine and Au/Ag/Cu-doped anatase surfaces, Tables S6 and S12: Energy gain (Ry) calculated for optimized structures of various glutamine (L) conformations on pristine 001 and 101 anatase slab surfaces, Tables S7 and S13: Interaction energies (Ry) of systems of various unbroken glutamine (L) molecule conformations on 001 and 101 pristine anatase surfaces, Tables S8 and S14: Energy gain (Ry) calculated for optimized structures of various glutamine (L) conformations on Au/Ag/Cu-doped 001 and 101 anatase slab surfaces, Tables S9 and S15: Interaction energies (Ry) of systems of various unbroken glutamine (L) molecule conformations (Orient-1, -3, -4, -6, and -7) on Au/Ag/Cu-doped 001 and 101 anatase surfaces, Tables S10, S11, S16 and S17: Interatomic distances of physical/chemical interactions noticed in systems of various glutamine (L) molecule conformations on 001 and 101 pristine and doped anatase slab surfaces, Tables S18 and S19: Interaction types of optimized glutamine (L) various conformations with pristine and doped 001 and 101 anatase slab surface systems, Tables S20 and S21: Calculated energy differences of the initial state and unbroken/broken or non-separated H atom/separated H atom molecule state for a number of initial configurations, converted into local temperatures (K), for the 001- and 101-surface-type systems. Here, we assume that the energy release has been equally distributed over the 20 atoms in the glutamine molecule, Figures S1, S5–S14 and S25–S39: Visualized various glutamine (L) molecule conformations (Orient-1, -3, -4, -6, and -7, Minimum 1–4) on pristine 001 anatase surfaces, Figures S2 and S40–S54: Various glutamine (L) molecule conformations (Orient-1, -3, -4, -6, and -7) on Au/Ag/Cu-doped 001 anatase surfaces, Figure S3, S15–S24 and S55–S69: Visualized various glutamine (L) molecule conformations (Orient-3, -4, -6, -7, and -9, Minimum 1–4) on pristine 101 anatase surfaces, Figures S4 and S70–S84: Visualized various glutamine (L) molecule conformations (Orient-3, -4, -6, -7, and -9) on Au/Ag/Cu-doped 101 anatase surfaces, Figures S85–S90: Visualized electron densities of various glutamine (L) molecule conformations on pristine and Au/Ag/Cu-doped 001 and 101 anatase surfaces.
