# Supplementary material for: Energy Landscape of Relaxation and Interaction of an Amino Acid, Glutamine (L), on Pristine and Au/Ag/Cu-Doped TiO2 Surfaces
Source: Nanomaterials (Basel). 2023 Sep 30;13(19):2688. doi: 10.3390/nano13192688 (PMC10574630; doi:10.3390/nano13192688)
Supplement: Supplementary file 1 [file nanomaterials-13-02688-s001.zip › nanomaterials-2501956-supplementary.pdf]

Supplementary Materials

Table S1. An overview of the electronic features of dopant atoms used in this study [1].

Element/ Dopant	Oxidation state	Atomic number (Z)	Atomic radius (pm)	Electronic configuration
Ti	+4	22	147	[Ar] $3d^2$ $4s^2$
Au	+3, +1	79	144	[Xe] $4f^{14}$ $5d^{10}$ $6s^1$
Ag	+1	47	144	[Kr] $4d^{10}$ $5s^1$
Cu	+2, +1	29	128	[Ar] $3d^{10}$ $4s^1$

Table S2. Total energies [Ry] of systems of various Glutamine (L) molecule conformations (Orient 1, 3, 4, 6, and 7) on 001 pristine anatase surfaces.

System	Orient 1	Orient 3	Orient 4	Orient 6	Orient 7
**Minimum 1**	−3503.9665405	−3504.0123309	−3504.0150932	−3503.9622164	−3503.9631739
**Minimum 2**	−3504.0640618	−3504.0344883	−3504.0441305	−3503.9623672	−3503.9632939
**Minimum 3**	−3504.0289059	−3504.0344684	−3503.9597967	−3503.9950844	−3503.9647623
**Minimum 4**	−3504.0284921	−3504.0338616	−3503.8987253	−3503.9916856	−3504.0509188

*The lowest total energy of an unbroken molecule's system is colored in red; The energies of broken molecule structures are colored in yellow, and the lowest energy of this type of structure is marked in green.

Table S3. Total energies [Ry] of systems of various Glutamine (L) molecule conformations (Orient 1, 3, 4, 6, and 7) on 001 Au / Ag / Cu -doped anatase surfaces.

System	Orient 1	Orient 3	Orient 4	Orient 6	Orient 7
**Au-doped**	−3499.8348464	−3499.9391179	−3499.8401899	−3499.7434322	−3499.7430405
**Ag-doped**	−3680.9600300	−3681.0389907	−3681.2178991	−3681.1920492	−3680.8670047
**Cu-doped**	−3786.6187830	−3786.7095249	−3786.8429452	−3786.6176504	−3786.5948840

* The lowest total energies for each type of surface for whole molecules are colored in red, while broken structures are colored in yellow, and their lowest energies are marked in green.

Table S4. Total energies [Ry] of systems of various Glutamine (L) molecule conformations (Orient 3, 4, 6, 7, and 9) on 101 pristine anatase surfaces.

System	Minimum 1	Minimum 2	Minimum 3	Minimum 4
**Orient 3**	−3502.8028739	−3502.8039039	−3502.8242836107	−3502.8244786
**Orient 4**	−3502.8226043	−3502.8226410	−3502.8222355	−3502.8060474
**Orient 6**	−3502.7801018	−3502.8007256	−3502.8043085	−3502.7812959
**Orient 7**	−3502.7783099	−3502.7797589	−3502.7790869	−3502.7906431
**Orient 9**	−3502.7952086	−3502.7938126	−3502.7965273	−3502.7959975

*The lowest total energy of an unbroken molecule's system is colored in red; The energies of broken molecule structures are colored in yellow, and the lowest energy is marked in green.

Table S5. Total energies [Ry] of systems of various Glutamine (L) molecule conformations (Orient 3, 4, 6, 7, and 9) on 101 Au / Ag / Cu -doped anatase surfaces.

System	Au-doped	Ag-doped	Cu-doped
**Orient 3**	−3498.7410649	−3679.8244536	−3785.4294650
**Orient 4**	−3498.7042240	−3679.7189948	−3785.3912780
**Orient 6**	−3498.7446577	−3679.8171251	−3785.3406376
**Orient 7**	−3498.6128959	−3679.7130683	−3785.3561339
**Orient 9**	−3498.7046715	−3679.7843836	−3785.3841755

* The lowest total energies for each type of surface for whole molecules are colored in red, while broken structures are colored in yellow, and their lowest energies are marked in green.

Table S6. Energy gain [Ry] calculated for optimized structures of various Glutamine (L) conformations (Orient 1, 3, 4, 6, and 7) on pristine 001 anatase slab surfaces.

System	Orient 1	Orient 3	Orient 4	Orient 6	Orient 7

**Minimum 1**	−0.2945805	−0.3403709	−0.3431332	−0.2902564	−0.2912139
**Minimum 2**	−0.3920979	−0.3625283	−0.3721705	−0.2904072	−0.2913339
**Minimum 3**	−0.3569459	−0.3625084	−0.2878367	−0.3231244	−0.2928023
**Minimum 4**	−0.3565321	−0.3619016	−0.2267653	−0.3197256	−0.3789549

*The lowest energy gain for an unbroken molecule's system is colored in red; The gain energies of broken molecule structures are colored in yellow, and the lowest energy is marked in green.

Table S7. Interaction energies [Ry] of systems of various unbroken Glutamine (L) molecule conformations (Orient 1, 3, 4, 6, and 7) on 001 pristine anatase surfaces.

System	Orient 1	Orient 3	Orient 4	Orient 6	Orient 7
**Minimum 1**	−0.0128905	−0.0811909	−0.1504532	−0.1198564	−0.0065839
**Minimum 2**	−0.1950718	−0.1779383	−0.1589205	−0.0068672	−0.0066539
**Minimum 3**	−0.1721059	−0.1784484	broken	broken	−0.006292
**Minimum 4**	−0.1720821	broken	broken	−0.0086656	broken

*The lowest interaction energy for an unbroken molecule's system is colored in red, while broken structures are colored in yellow.

Table S8. Energy gain [Ry] calculated for optimized structures of various Glutamine (L) conformations (Orient 1, 3, 4, 6, and 7) on Au / Ag / Cu-doped 001 anatase slab surfaces.

System	Orient 1	Orient 3	Orient 4	Orient 6	Orient 7
**Au-doped**	−0.3744964	−0.4787660	−0.3798380	−0.2830822	−0.2826905
**Ag-doped**	−0.3772934	−0.4562507	−0.6351625	−0.6093092	−0.2842647

Cu-doped	−0.4031068	−0.4938449	−0.6272690	−0.4019704	−0.379204

*The lowest energy gain for each unbroken molecule's system is colored in red; The gain energies of broken molecule structures are colored in yellow, and the lowest energies are marked in green.

Table S9. Interaction energies [Ry] of systems of various unbroken Glutamine (L) molecule conformations (Orient 1, 3, 4, 6, and 7) on 001 Au / Ag / Cu-doped anatase surfaces.

System	Orient 1	Orient 3	Orient 4	Orient 6	Orient 7
**Au-doped**	−0.1779764	broken	−0.1928299	−0.0088822	−0.0071105
**Ag-doped**	−0.0859600	broken	broken	broken	−0.0084447
**Cu-doped**	−0.0952430	broken	broken	−0.0687404	−0.0613540

*The lowest interaction energies for the unbroken molecule systems are colored in red, while broken structures are colored in yellow.

Table S10. Interatomic distances of physical/chemical interactions noticed in systems of various glutamine (L) molecule conformations (Orient 1, 3, 4, 6, and 7) on 001 pristine anatase slab surfaces.

System	Orient 1	Orient 3	Orient 4	Orient 6	Orient 7
**Minimum 1**	-	-	O2---Ti8 1.970 Å	-	-
**Minimum 2**	H7---O5 1.476 Å<br>O2---Ti8 1.928 Å	H2---O5 1.515 Å	H7---O6 2.035 Å<br>O2---Ti8 1.984 Å	-	-
**Minimum 3**	H7---O5 1.445 Å<br>O2---Ti8 1.984 Å	H2---O5 1.509 Å	H7---O6 1.470 Å<br>O2---Ti9 1.956 Å<br>H2---O9 1.919 Å<br>H10---O11 0.989 Å<br>Molecule's separated linear chain part:<br>C2---O3 1.171 Å	Separated H atom:<br>H10---O11 0.990 Å<br>Molecule's separated linear chain part:<br>C2---O1 1.171 Å<br>C2---O3 1.176 Å	-

			C2---O1 1.176 Å		
**Minimum 4**	H7---O5 1.445 Å<br><br>O2---Ti8 1.984 Å	H2---O5 1.038 Å<br><br>N1---Ti8 2.024 Å<br><br>O1---Ti9 2.271 Å	Broken molecule:<br><br>H7---O6 1.023 Å<br><br>H10---O11 0.985 Å<br><br>Molecule's separated linear chain part:<br><br>C2---O3 1.173 Å<br><br>C2---O1 1.174 Å	H7---O5 1.445 Å<br><br>O2---Ti8 1.984 Å	H7---O5 1.003 Å<br><br>O2---Ti9 2.140 Å

* The interatomic distances of broken molecule structures are marked in yellow.

Table S11. Interatomic distances of physical/chemical interactions noticed in systems of various glutamine (L) molecule conformations (Orient 1, 3, 4, 6, and 7) on 001 Au / Ag / Cu -doped anatase slab surfaces.

**System**	**Orient 1**	**Orient 3**	**Orient 4**	**Orient 6**	**Orient 7**
**Au-doped**	H7---O5 1.490 Å<br><br>O2---Au1 2.057 Å	H2---O5 1.447 Å<br><br>N1---Au1 2.066 Å<br><br>Separated part:<br><br>H10---O11 0.989 Å<br><br>O3---O10 2.028 Å	H7---O5 1.516 Å<br><br>O2---Au1 2.066 Å	-	-
**Ag-doped**	H7---O5 1.707 Å	H2---O5 1.434 Å<br><br>Separated part:<br><br>H10---O11 0.991 Å<br><br>O3---H10 1.969 Å	H7---O6 1.459 Å<br><br>H2---O8 1.824 Å<br><br>H10---O11 0.989 Å<br><br>Molecule's separated linear chain part:<br><br>C2---O3 1.169 Å<br><br>C2---O1 1.177 Å	H9---O5 1.677 Å<br><br>H2---O11 1.889 Å<br><br>H10---O11 0.988 Å<br><br>Molecule's separated linear chain part:<br><br>C2---O3 1.173 Å<br><br>C2---O1 1.174 Å	-
**Cu-doped**	H7---O5 1.643 Å	H2---O5 1.038 Å	H7---O6 1.693 Å	H2---O11 1.964 Å	H7---O5 1.782 Å

	O2---Au1 2.033 Å	N1---Cu1 1.900 Å<br>O1---Ti8 2.022 Å<br>Separated part:<br>H10---O11 0.994 Å<br>O3---H10 1.859 Å	H8---O5 1.675 Å<br>H10---O11 0.985 Å<br>Molecule's separated linear chain part:<br>C2---O3 1.172 Å<br>C2---O1 1.175 Å		

* The interatomic distances of broken molecule structures are marked in yellow.

Table S12. Energy gain [Ry] calculated for optimized structures of various Glutamine (L) conformations (Orient 3, 4, 6, 7, and 9) on pristine 101 anatase slab surfaces.

System	Minimum 1	Minimum 2	Minimum 3	Minimum 4
**Orient 3**	−0.3298049	−0.3096839	−0.3300634	−0.3302584
**Orient 4**	−0.3283843	−0.328421	−0.3280155	−0.3118274
**Orient 6**	−0.2858818	−0.3065056	−0.3100885	−0.2870759
**Orient 7**	−0.2840899	−0.2855389	−0.2848669	−0.2964231
**Orient 9**	−0.3009886	−0.2995926	−0.3023073	−0.3017775

*The lowest energy gain for an unbroken molecule's system is colored in red; The gain energies of broken molecule structures are colored in yellow, and the lowest energy is marked in green.

Table S13. Interaction energies [Ry] of systems of various unbroken Glutamine (L) molecule conformations (Orient 3, 4, 6, 7, and 9) on 101 pristine anatase surfaces.

System	Minimum 1	Minimum 2	Minimum 3	Minimum 4
**Orient 3**	H separated	H separated	H separated	−0.0750086
**Orient 4**	−0.0582243	−0.0589310	−0.0575155	−0.0342674
**Orient 6**	H separated	H separated	H separated	H separated
**Orient 7**	−0.0227599	−0.0251489	−0.0235069	−0.0497531
**Orient 9**	−0.0381086	−0.0418826	−0.0410673	−0.0392875

*The lowest interaction energy for the unbroken molecule system is colored in red, while broken structures are colored in yellow.

Table S14. Energy gain [Ry] calculated for optimized structures of various Glutamine (L) conformations (Orient 3, 4, 6, 7, and 9) on Au / Ag / Cu-doped 101 anatase slab surfaces.

System	Au-doped	Ag-doped	Cu-doped
**Orient 3**	−0.4132849	−0.3974649	−0.3847780
**Orient 4**	−0.3764440	−0.2920048	−0.346588
**Orient 6**	−0.4168793	−0.3901364	−0.2959476
**Orient 7**	−0.2851159	−0.2860783	−0.3114439
**Orient 9**	−0.3768931	−0.3573936	−0.3394885

*The lowest energy gain for each unbroken molecule's system is colored in red; The gain energies of broken molecule structures are colored in yellow, and the lowest energies are marked in green.

Table S15. Interaction energies [Ry] of systems of various unbroken Glutamine (L) molecule conformations (Orient 3, 4, 6, 7, and 9) on 101 Au / Ag / Cu-doped anatase surfaces.

System	Au-doped	Ag-doped	Cu-doped
**Orient 3**	H separated	−0.1566136	−0.1506750
**Orient 4**	−0.1051139	−0.0386248	−0.0889582
**Orient 6**	H separated	H separated	H separated
**Orient 7**	−0.0214959	−0.0230283	−0.0618940
**Orient 9**	−0.1227615	−0.1081236	H separated

*The lowest interaction energies for the unbroken molecule systems are colored in red, while broken structures are colored in yellow.

Table S16. Interatomic distances of physical/chemical interactions noticed in systems of various glutamine (L) molecule conformations (Orient 3, 4, 6, 7, and 9) on 101 pristine and Au / Ag/ Cu pristine anatase surfaces.

System	Minimum 1	Minimum 2	Minimum 3	Minimum 4
**Orient 3**	H separation:<br>C3---H4 1.232 Å	H separation:<br>C3---H4 1.225 Å	H10---O11 1.574 Å<br>H separation:	H10---O11 1.560 Å

	C4---H6 1.225 Å	C4---H6 1.222 Å	C3---H4 1.212 Å	
**Orient 4**	O2---Ti8 2.295 Å<br>H9---O8 2.039 Å	O2---Ti8 2.279 Å<br>H9---O8 2.044 Å	O2---Ti8 2.298 Å<br>H9---O8 1.989 Å	-
**Orient 6**	H2---O11 1.907 Å<br>O1---H2 1.975 Å<br>H separation:<br>C4---H5 1.270 Å<br>C1---H1 1.272 Å<br>C3---H3 1.268 Å	H10---O20 2.025 Å<br>H separation:<br>C3---H3 1.211 Å<br>C1---H1 1.213 Å	H separation:<br>C4---C5 1.263 Å<br>C3---H3 1.208 Å<br>C1---H1 1.210 Å	H2---O11 1.934 Å<br>O1---H2 1.975 Å<br>H separation:<br>C3---H5 1.266 Å<br>C1---H1 1.271 Å<br>C4---H5 1.270 Å
**Orient 7**	-	-	-	H10---O11 1.665 Å
**Orient 9**	H2---O5 2.076 Å	H2---O5 1.990 Å	H2---O5 2.003 Å	H2---O5 2.097 Å

* The interatomic distances of broken molecule structures are marked in yellow.

Table S17. Interatomic distances of physical/chemical. interactions noticed in systems of various glutamine (L) molecule conformations (Orient 3, 4, 6, 7, and 9) on 101 Au / Ag/ Cu -doped anatase surfaces.

**System**	**Au-doped**	**Ag-doped**	**Cu-doped**
**Orient 3**	N1---Au1 2.145 Å<br>H10---O11 1.557Å<br>H-separation:<br>C3---H4 1.208 Å	H10---O11 1.419 Å<br>H2---O5 2.075 Å	H10---O11 1.398 Å<br>N1---Cu1 2.004 Å<br>H2---O5 1.896 Å
**Orient 4**	O2---Au1 2.136 Å<br>H9---O8 1.989 Å	H9---O8 1.879 Å	H9---O8 1.952 Å<br>N1---Cu1 2.092 Å<br>H7---O5 1.794 Å<br>H8---O20 2.042 Å
**Orient 6**	N1---Au1 2.102 Å<br>H10---O20 1.626 Å<br>O1---H8 2.046 Å	H10---O20 1.586 Å<br>H separation:<br>C3---H3 1.245 Å	H2---O11 1.764 Å<br>H separation:<br>C3---H3 1.267 Å

	H separation:<br>C3---H3 1.215 Å	C1---H1 1.231 Å	C1---H1 1.276 Å<br>C4---H5 1.265 Å
**Orient 7**	-	-	H10---O20 1.714 Å<br>H8---O5 2.064 Å
**Orient 9**	N1---Au1 2.150 Å<br>H2---O5 1.925 Å	H2---O5 1.648 Å	N1---Cu1 2.044 Å<br>H2---O5 1.634 Å<br>H separation:<br>C3---H4 1.210 Å<br>C4---H6 1.210 Å

* The interatomic distances of broken molecule structures are marked in yellow.

Table S18. Interaction types of optimized Glutamine (L) various conformations with pristine and doped 001 anatase slab surface system.

**Interaction Type 001**	**Physical interaction**	**Chemical interaction**	**Broken molecule**
**Pristine**	Orient 1 – Minimum 1<br>Orient 3 – Minimum 1, 2 3<br>Orient 6 – Minimum 1, 2<br>Orient 7 - Minimum 1, 2, 3<br>Orient 2, 5, 8, 9, 10 – Minimum 1	Orient 1 – Minimum 2, 3, 4<br>Orient 4 – Minimum 1, 2<br>Orient 6 – Minimum 4	Orient 3 – Minimum 4<br>Orient 4 – Minimum 3, 4<br>Orient 6 – Minimum 3<br>Orient 7 – Minimum 4
**Au-doped**	Orient 6<br>Orient 7	Orient 1<br>Orient 4	Orient 3
**Ag-doped**	Orient 1<br>Orient 7	*Orient 3<br>*Orient 4	Orient 3<br>Orient 4<br>Orient 6
**Cu-doped**	Orient 6<br>Orient 7	Orient 1<br>*Orient 3<br>*Orient 4	Orient 3<br>Orient 4

*Orients with an asterisk are with chemical interaction and broken molecule. Structures with the lowest energies are marked in yellow.

Table S19. Interaction types of optimized Glutamine (L) various conformations with pristine, and doped 101 anatase slab surface system.

Interaction Type 101	Physical interaction	Chemical interaction	Separated H atoms of a molecule
**Pristine**	Orient 3 – Minimum 4<br>Orient 4 – Minimum 4<br>Orient 7 – Minimu 1, 2, 3, 4<br>Orient 9 – Minimu 1, 2, 3, 4	Orient 4 – Minimum 1, 2, 3	Orient 3 – Minimum 1, 2, 3<br>Orient 6 – Minimum 1, 2, 3, 4
**Au-doped**	Orient 7	Orient 3<br>Orient 4<br>*Orient 6<br>Orient 9	Orient 3<br>Orient 6
**Ag-doped**	Orient 3<br>Orient 4<br>Orient 7<br>Orient 9	-	Orient 6
**Cu-doped**	Orient 7	Orient 3<br>Orient 4<br>*Orient 9	Orient 6<br>Orient 9

*Orients with an asterisk are with chemical interaction and broken molecule. Structures with the lowest energies are marked in yellow.

Table S20. Calculated energy differences of the initial state and unbroken/broken molecule-state for a number of initial configurations, converted into local temperatures [K], for the surface 001 type systems. Here, we assume that the energy released has been equally distributed over the 20 atoms in the glutamine molecule.

001	Un-broken		Broken	
**System**	**Orient**	**T [K]**	**Orient**	**T [K]**
**Pristine**	1	938	7	849
**Au – doped**	4	1203	3	2361
**Ag – doped**	1	1737	4	3706
**Cu – doped**	1	1291	4	2965

Table S21. Calculated energy differences of the initial state and unbroken/broken molecule-state for a number of initial configurations where the break-up of the molecule consisted of the loss of hydrogen atoms, converted into local temperatures [K], for the surface 101 type systems. Here, we assume that the energy released has been equally distributed over the 20 atoms in the glutamine molecule.

101	Non-separated H atoms		Separated H atoms	
**System**	**Orient**	**T [K]**	**Orient**	**T [K]**
**Pristine**	3	5474	3	5473
**Au – doped**	9	5077	6	6731
**Ag – doped**	3	7179	6	6271
**Cu – doped**	3	6133	9	3590

In Supplementary Material are presented figures of all investigated Glutamine (L) molecule conformations on pristine/un-doped and Au / Ag / Cu doped anatase 001 and 101 surfaces, visualized by the Vesta program.

<u>Atoms' color legend:</u>

Slabs: Ti – blue, O – red, Au – gold, Ag – silver, Cu – orange

Molecule: C – brown, O – red, N – light blue, H – rose

System	Minimum 1	Minimum 2	Minimum 3	Minimum 4
Orient 1	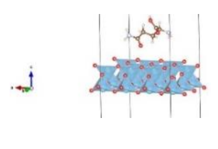	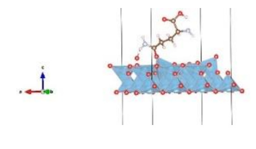	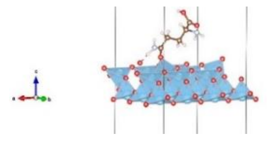	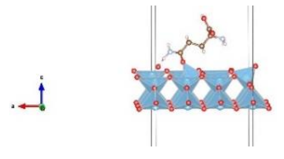
Orient 3	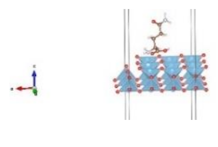	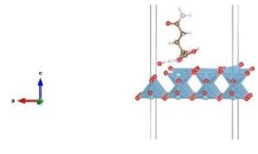	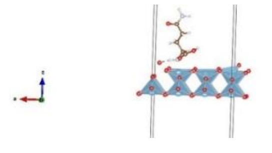	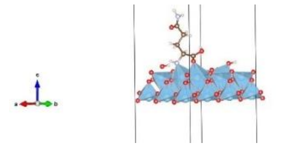
Orient 4	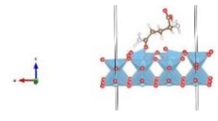	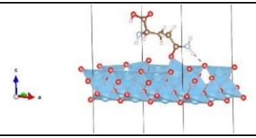	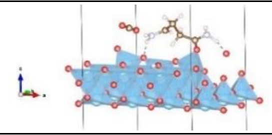	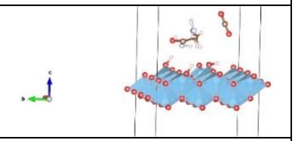
Orient 6	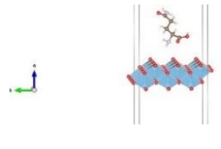	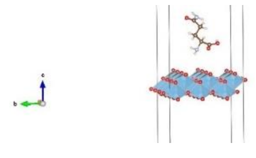	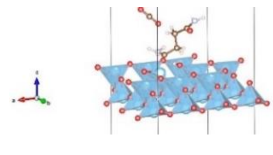	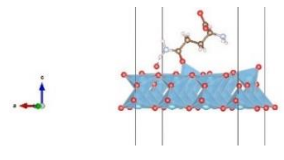
Orient 7	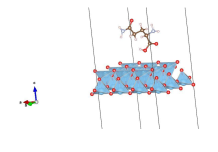	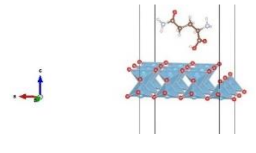	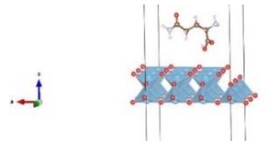	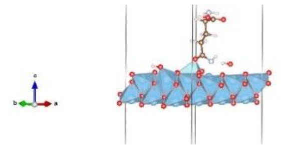

Figure S1. Visualized various Glutamine (L) molecule conformations (Orient 1, 3, 4, 6, and 7, Minimum 1–4) on pristine 001 anatase surfaces.

System	Au-doped	Ag-doped	Cu-doped
**Orient 1**	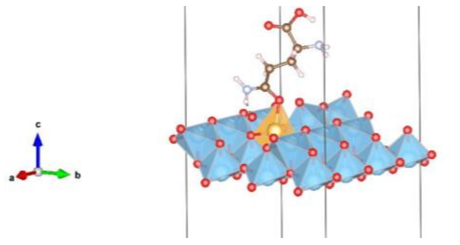	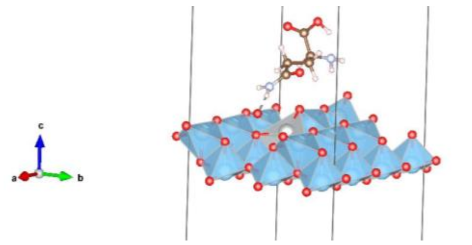	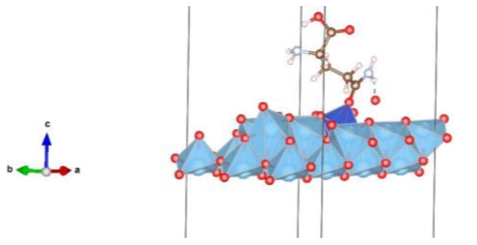
**Orient 3**	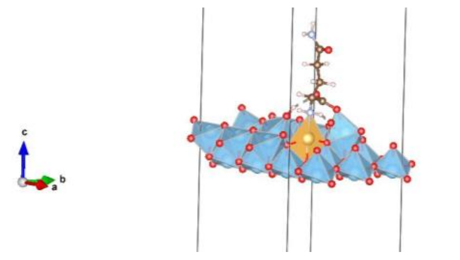	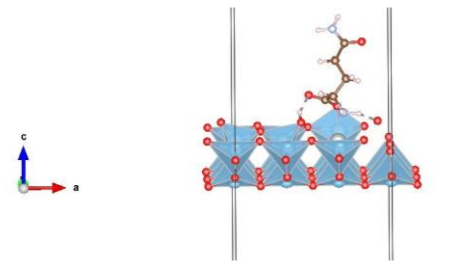	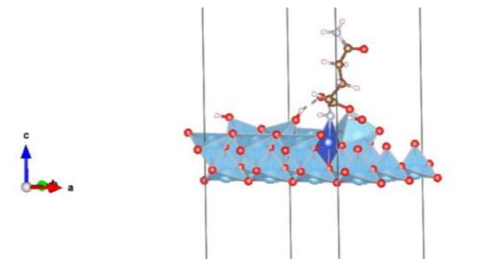
**Orient 4**	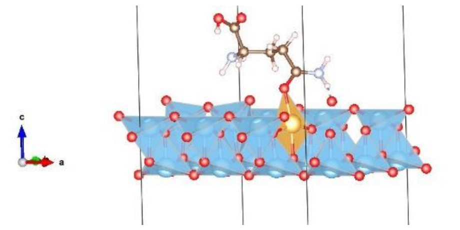	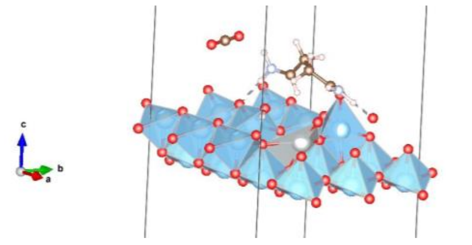	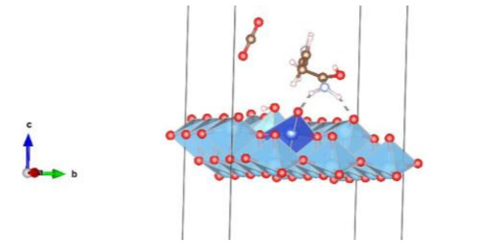
**Orient 6**	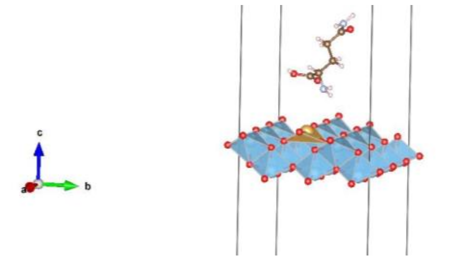	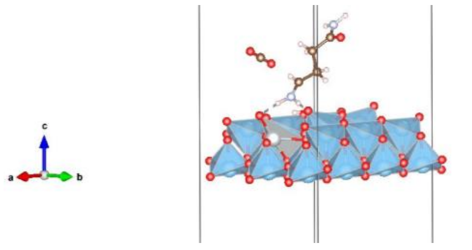	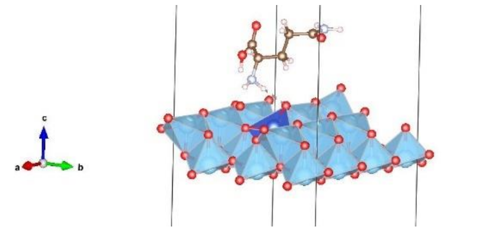
**Orient 7**	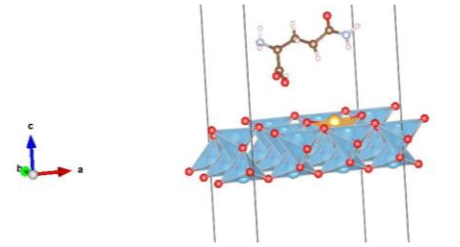	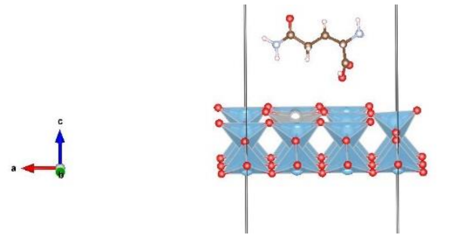	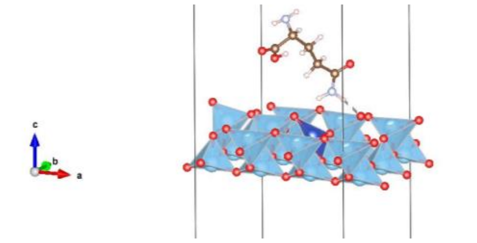

Figure S2. Various Glutamine (L) molecule conformations (Orient 1, 3, 4, 6, and 7) on Au / Ag / Cu -doped 001 anatase surfaces.

System	Minimum 1	Minimum 2	Minimum 3	Minimum 4
Orient 3	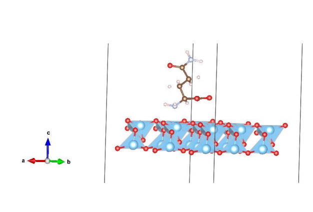	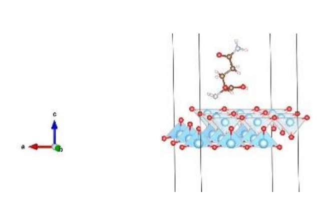	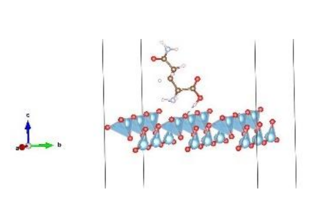	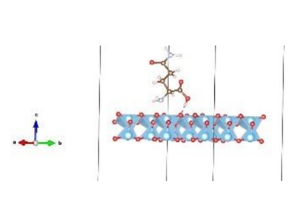
Orient 4	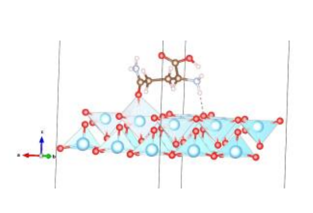	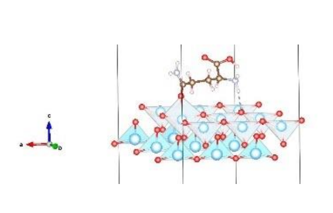	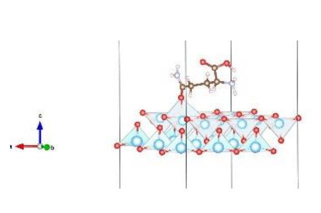	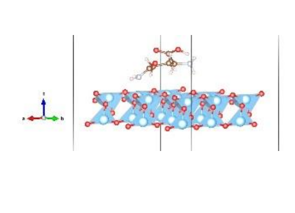
Orient 6	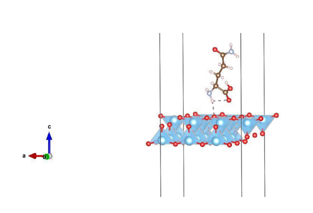	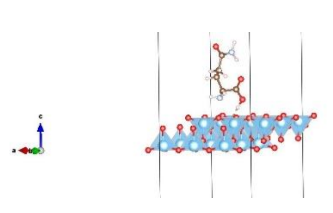	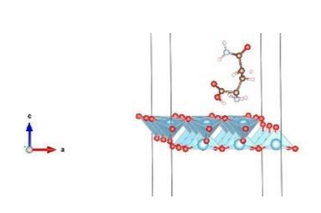	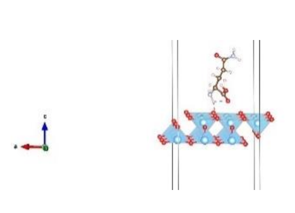
Orient 7	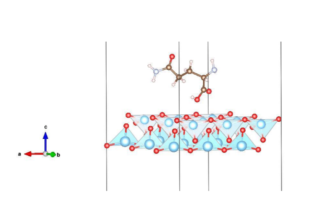	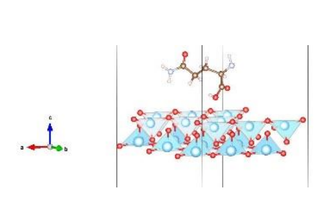	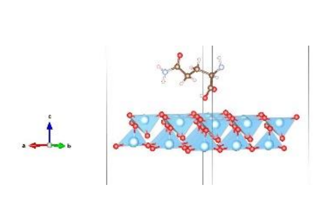	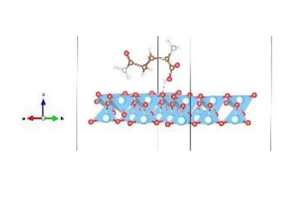
Orient 9	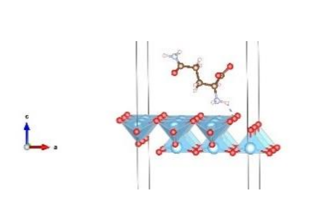	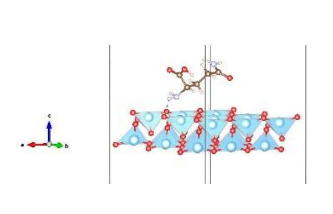	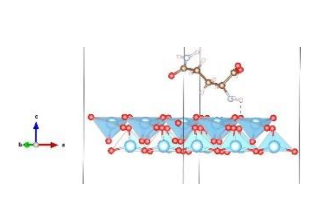	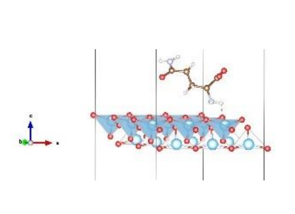

Figure S3. Visualized various Glutamine (L) molecule conformations (Orient 3, 4, 6, 7, and 9, Minimum 1–4) on pristine 101 anatase surfaces.

System	Au-doped	Ag-doped	Cu-doped
Orient 3	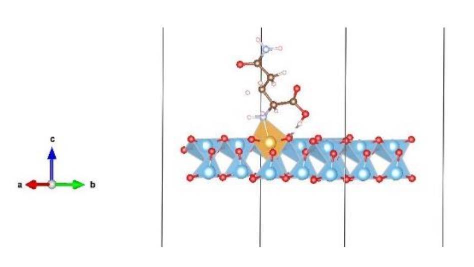	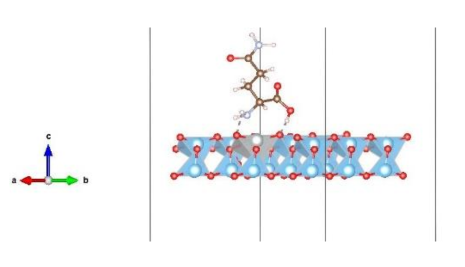	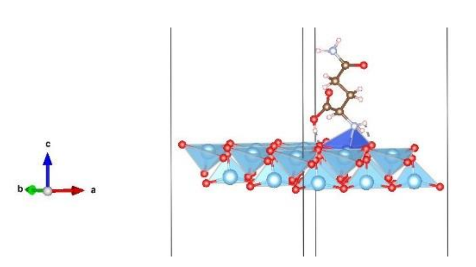

**Orient 4**	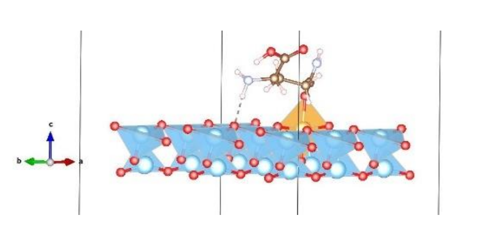	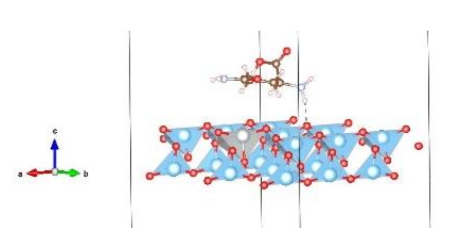	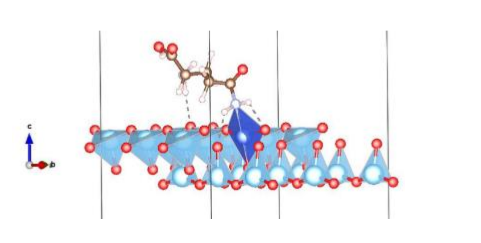
**Orient 6**	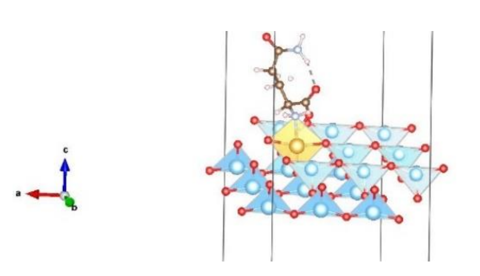	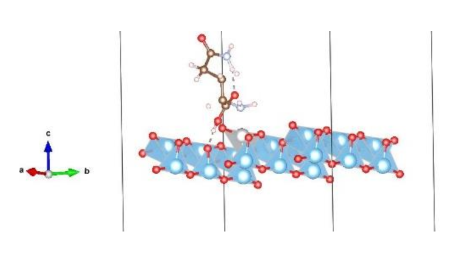	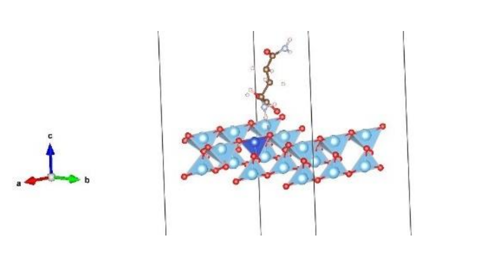
**Orient 7**	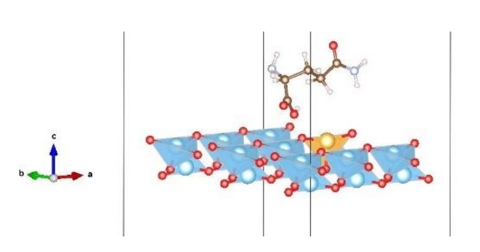	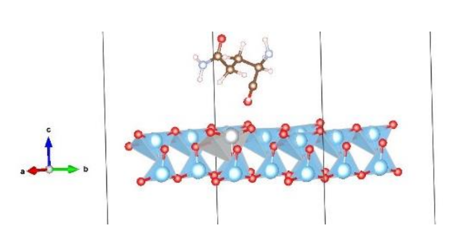	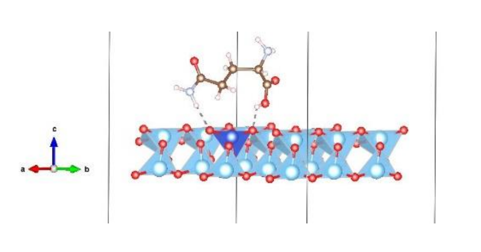
**Orient 9**	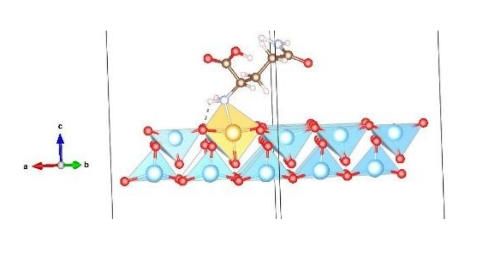	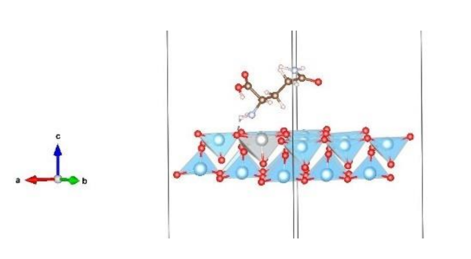	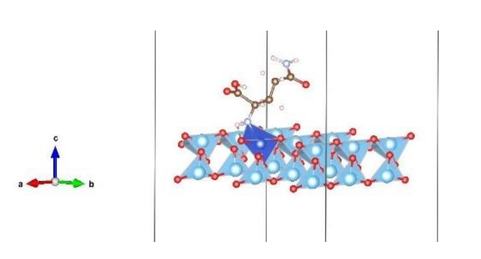

Figure S4. Visualized various Glutamine (L) molecule conformations (Orient 3, 4, 6, 7, and 9) on Au / Ag / Cu -doped 101 anatase surfaces.

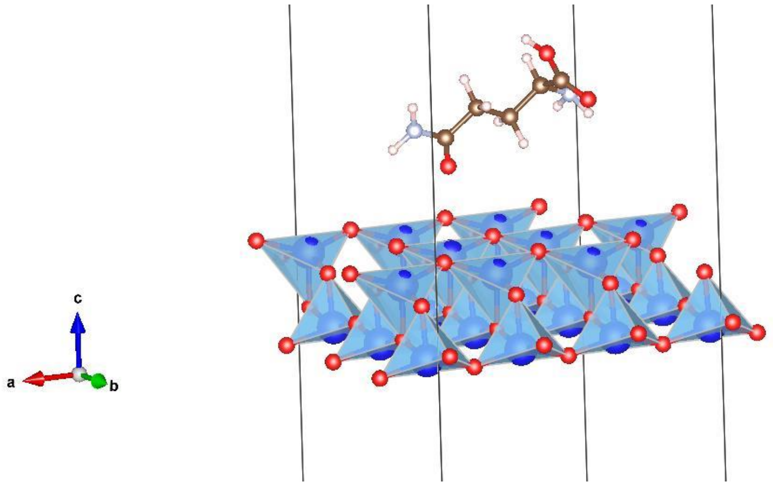

Figure S5. Optimized system Orient 1 Glutamine (L) molecule conformation on pristine 001 anatase slab surface (Minimum 1).

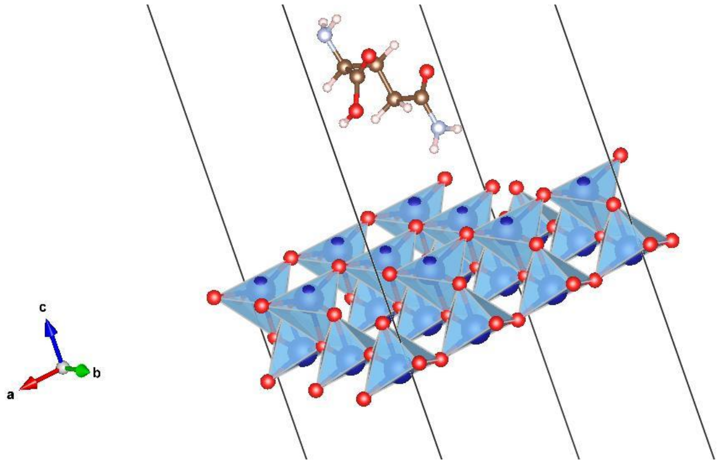

Figure S6. Optimized system Orient 2 Glutamine (L) molecule conformation on pristine 001 anatase slab surface (Minimum 1).

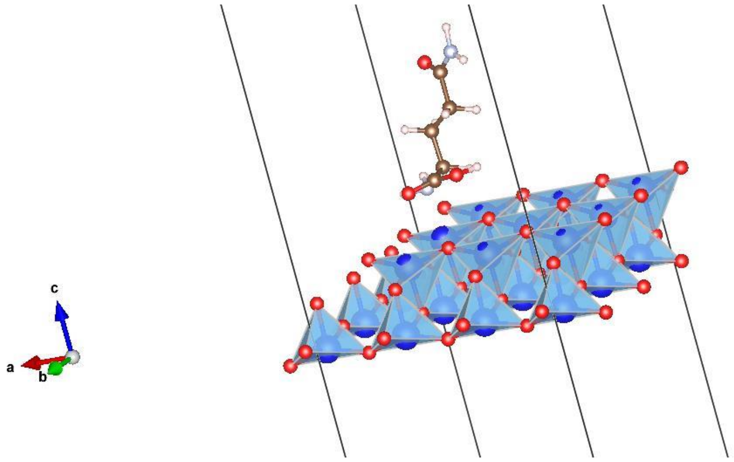

Figure S7. Optimized system Orient 3 Glutamine (L) molecule conformation on pristine 001 anatase slab surface (Minimum 1).

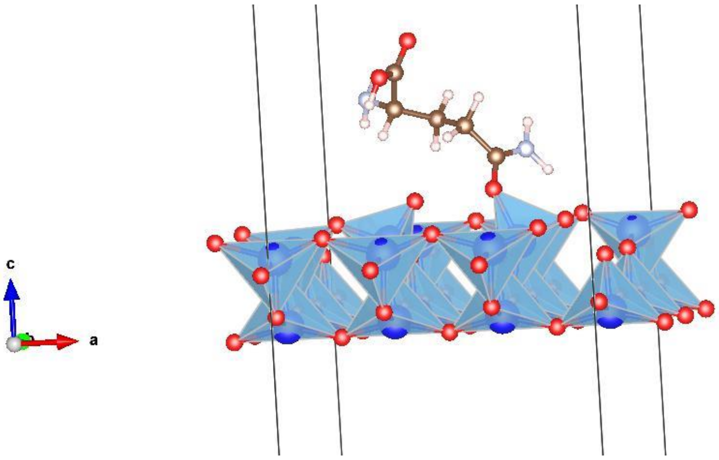

Figure S8. Optimized system Orient 4 Glutamine (L) molecule conformation on pristine 001 anatase slab surface (Minimum 1).

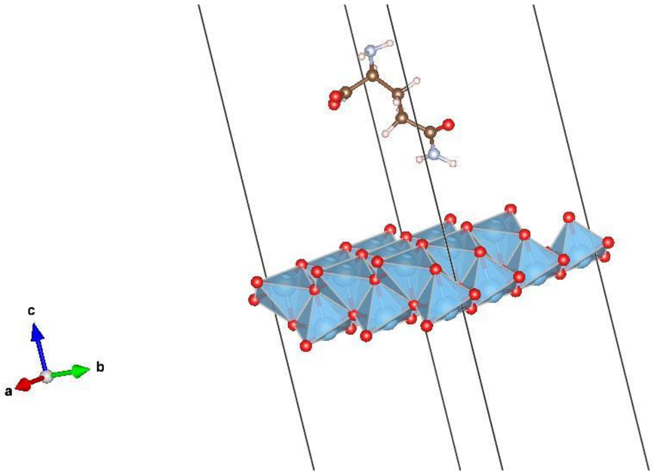

Figure S9. Optimized system Orient 5 Glutamine (L) molecule conformation on pristine 001 anatase slab surface (Minimum 1).

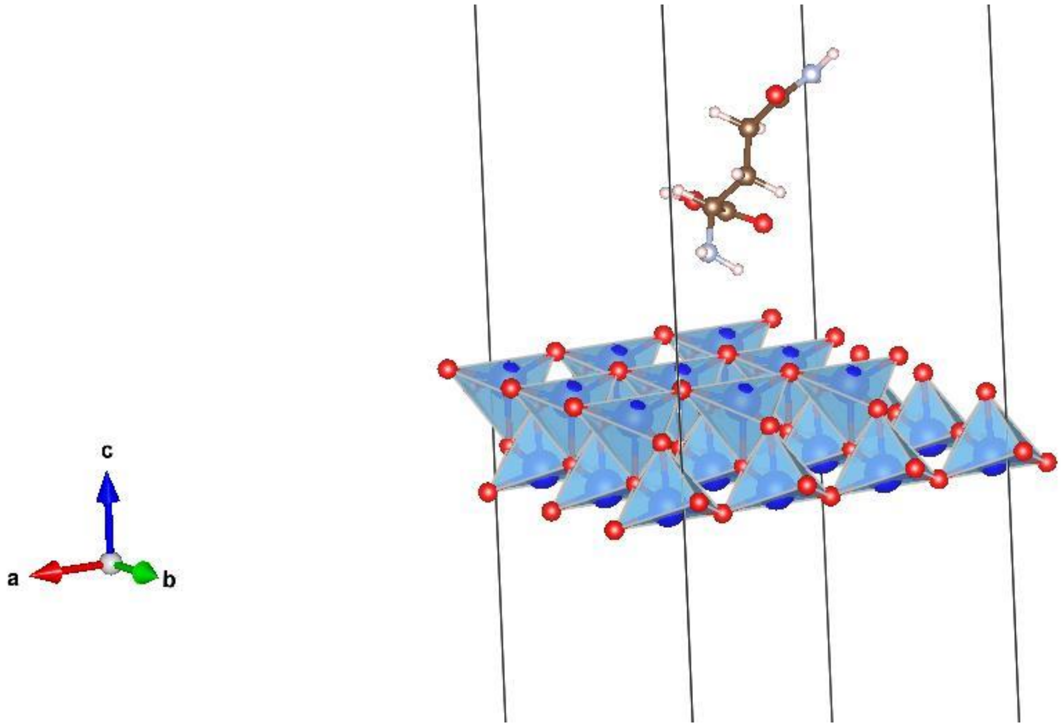

Figure S10. Optimized system Orient 6 Glutamine (L) molecule conformation on pristine 001 anatase slab surface (Minimum 1).

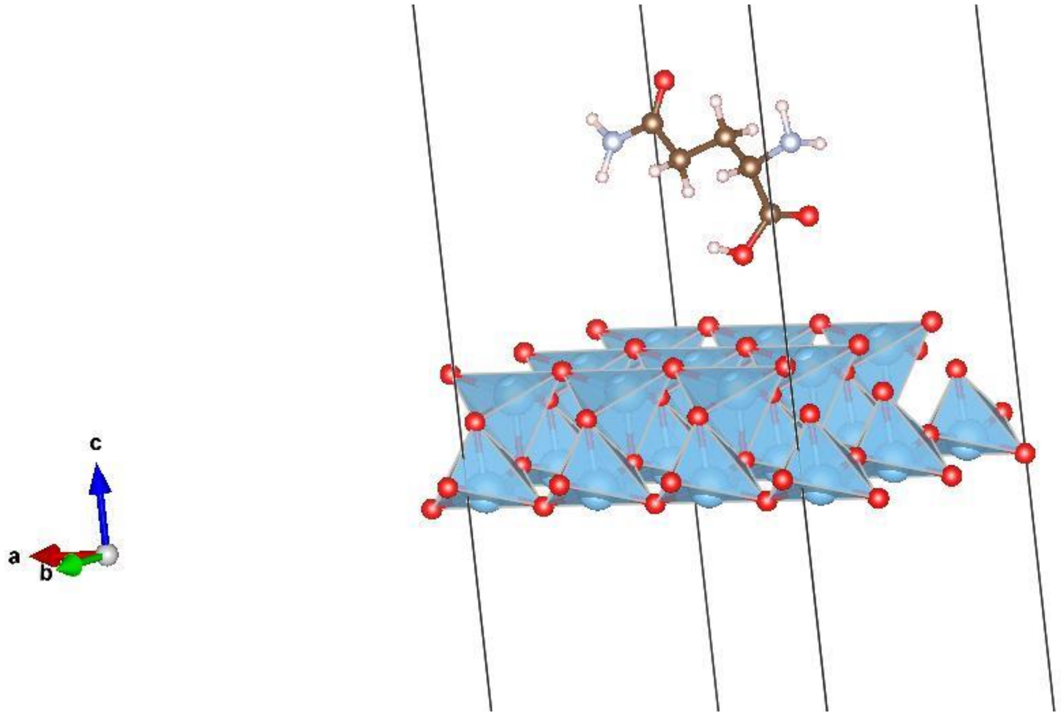

Figure S11. Optimized system Orient 7 Glutamine (L) molecule conformation on pristine 001 anatase slab surface (Minimum 1).

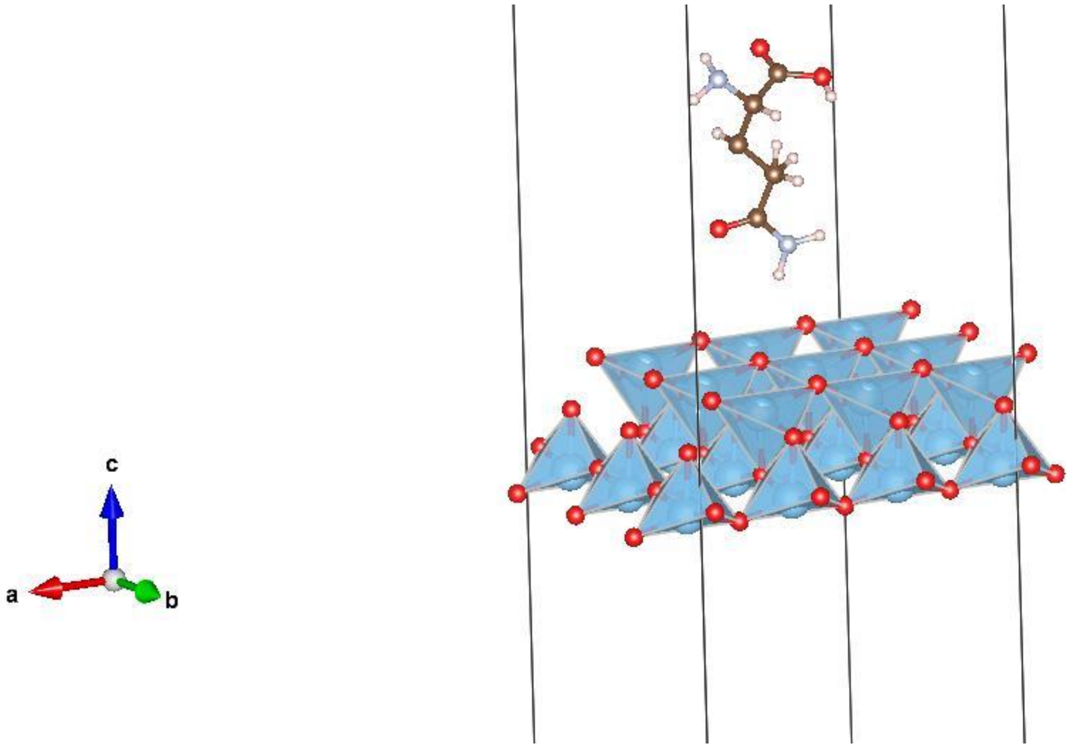

Figure S12. Optimized system Orient 8 Glutamine (L) molecule conformation on pristine 001 anatase slab surface (Minimum 1).

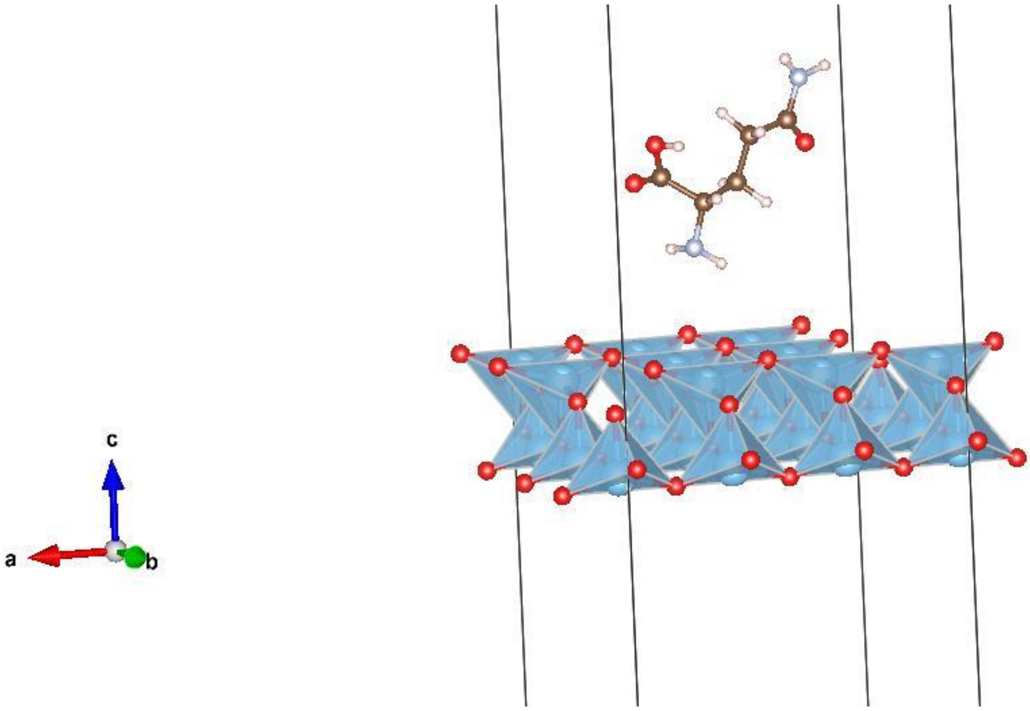

Figure S13. Optimized system Orient 9 Glutamine (L) molecule conformation on pristine 001 anatase slab surface (Minimum 1).

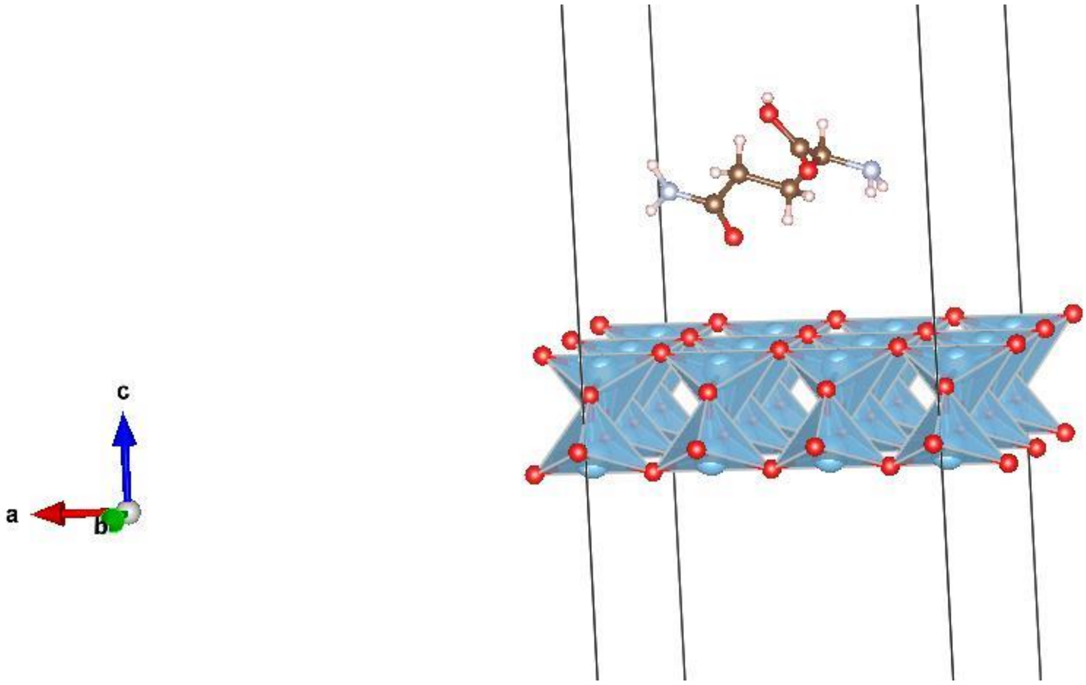

Figure S14. Optimized system Orient 10 Glutamine (L) molecule conformation on pristine 001 anatase slab surface (Minimum 1).

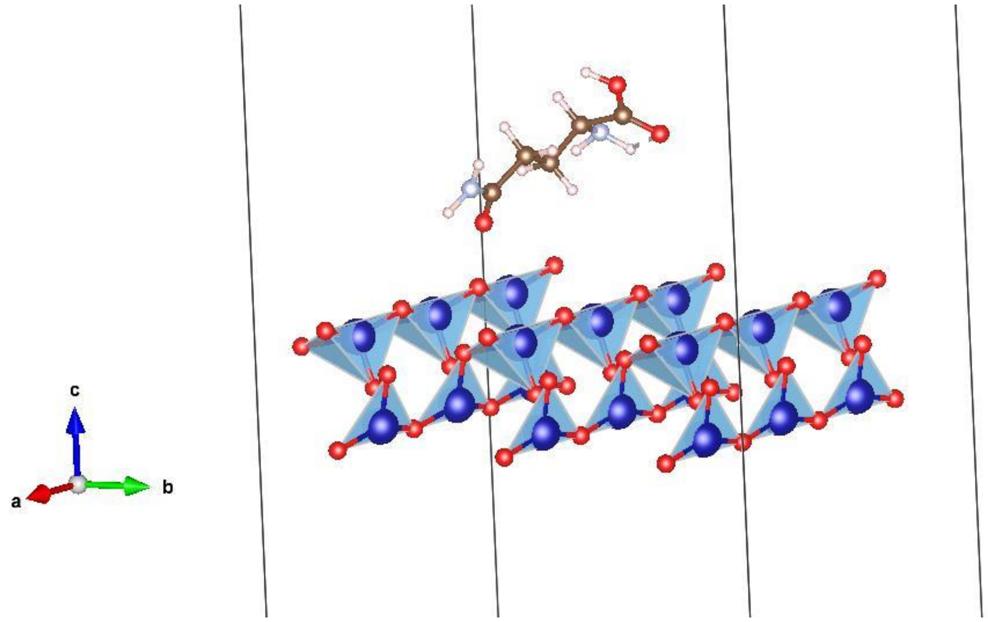

Figure S15. Optimized system Orient 1 Glutamine (L) molecule conformation on pristine 101 anatase slab surface (Minimum 1).

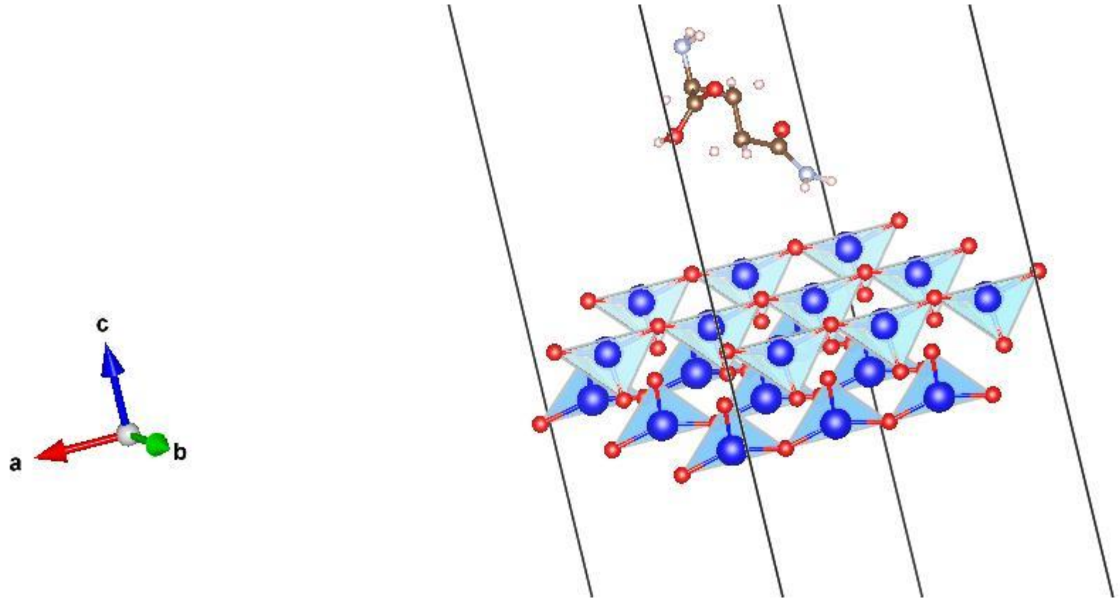

Figure S16. Optimized system Orient 2 Glutamine (L) molecule conformation on pristine 101 anatase slab surface (Minimum 1).

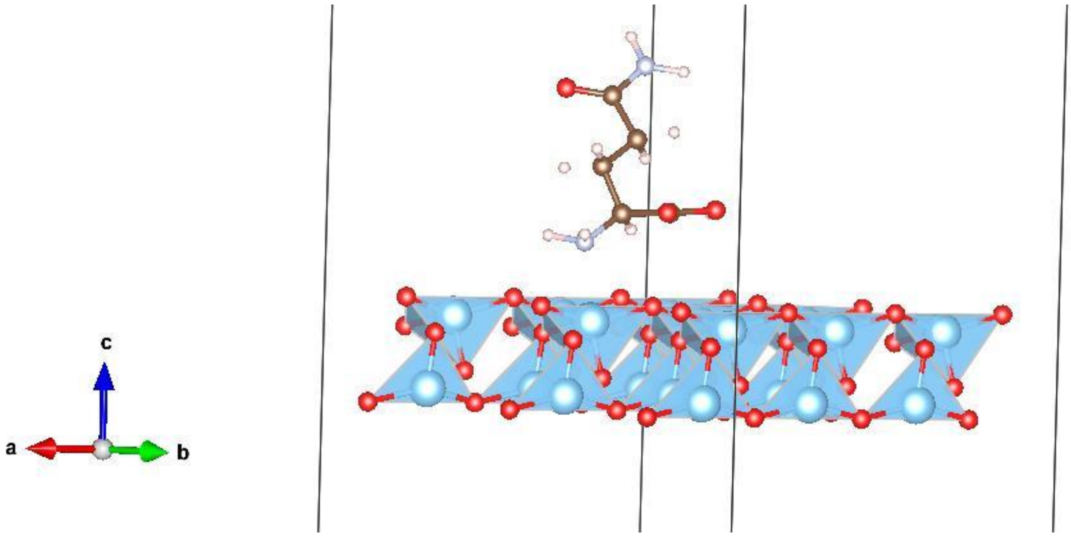

Figure S17. Optimized system Orient 3 Glutamine (L) molecule conformation on pristine 101 anatase slab surface (Minimum 1).

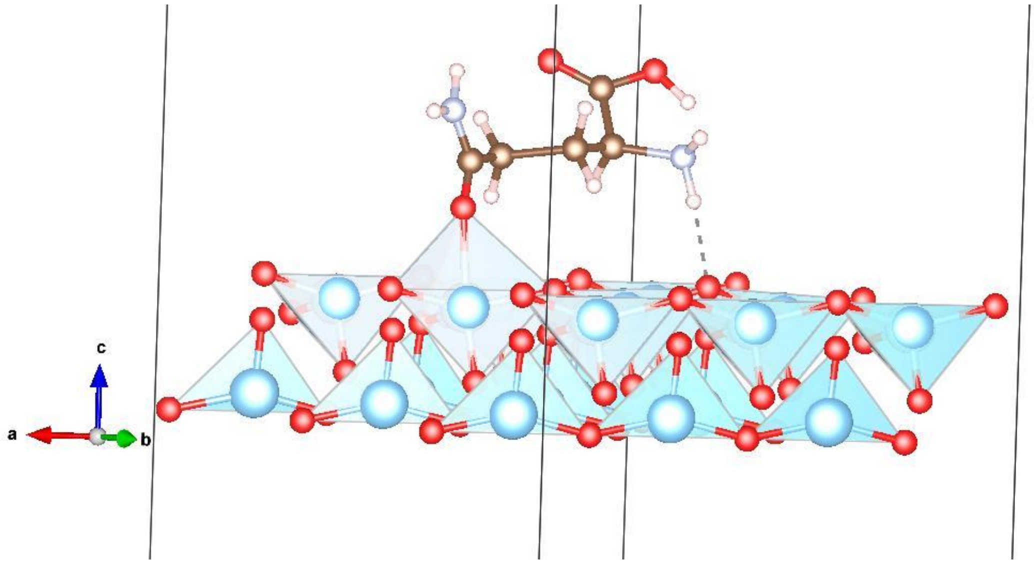

Figure S18. Optimized system Orient 4 Glutamine (L) molecule conformation on pristine 101 anatase slab surface (Minimum 1).

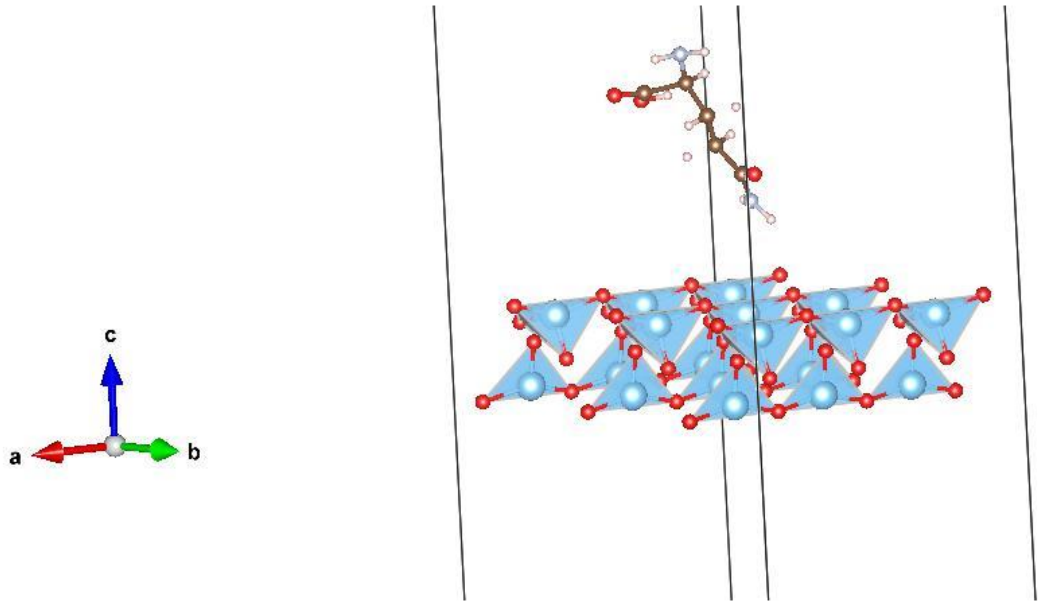

Figure S19. Optimized system Orient 5 Glutamine (L) molecule conformation on pristine 101 anatase slab surface (Minimum 1).

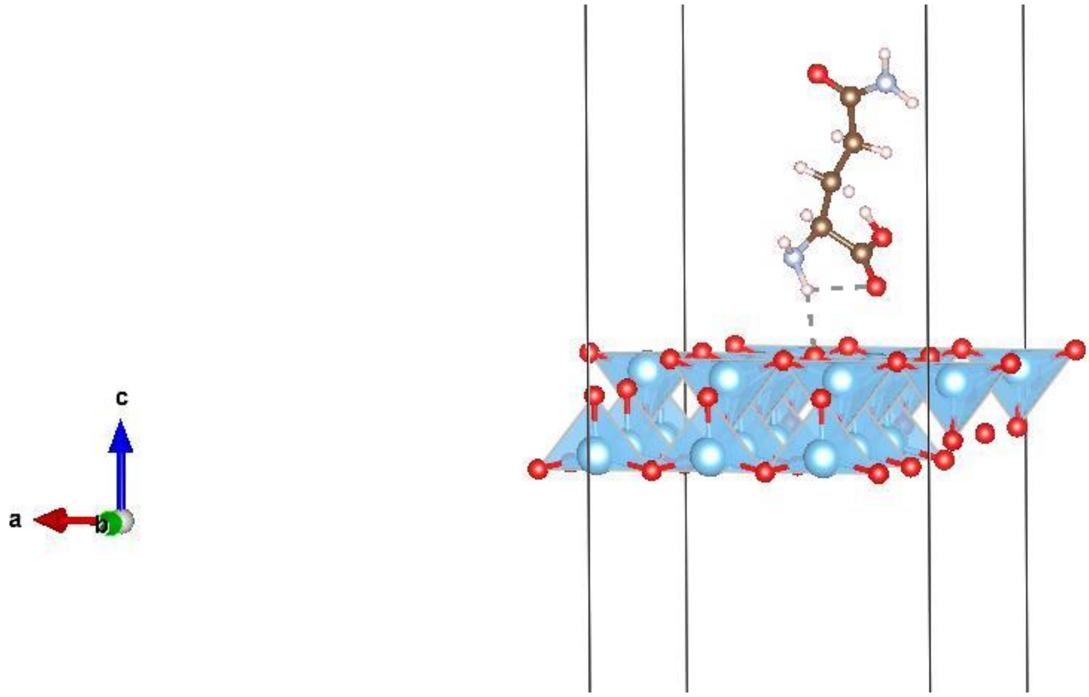

Figure S20. Optimized system Orient 6 Glutamine (L) molecule conformation on pristine 101 anatase slab surface (Minimum 1).

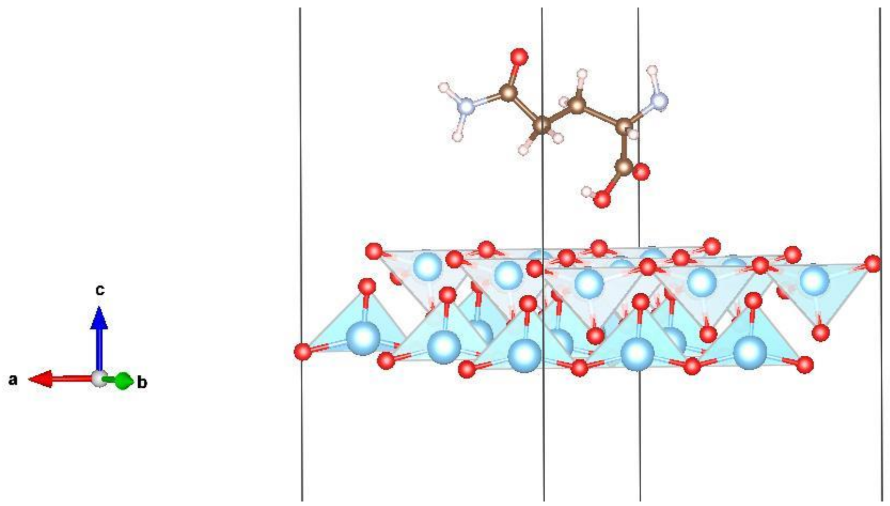

Figure S21. Optimized system Orient 7 Glutamine (L) molecule conformation on pristine 101 anatase slab surface (Minimum 1).

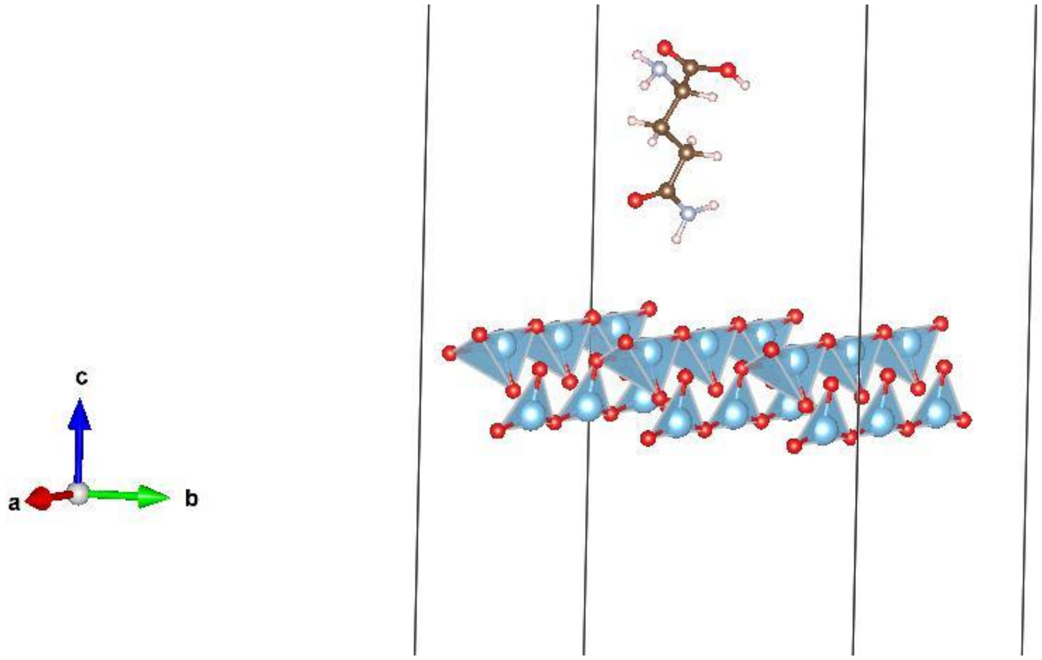

Figure S22. Optimized system Orient 8 Glutamine (L) molecule conformation on pristine 101 anatase slab surface (Minimum 1).

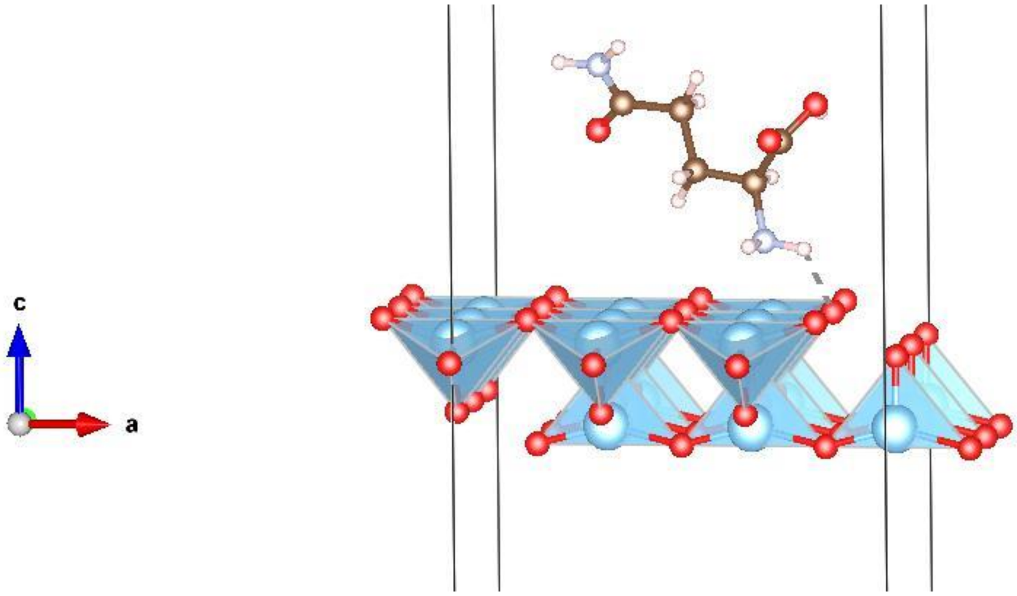

Figure S23. Optimized system Orient 9 Glutamine (L) molecule conformation on pristine 101 anatase slab surface (Minimum 1).

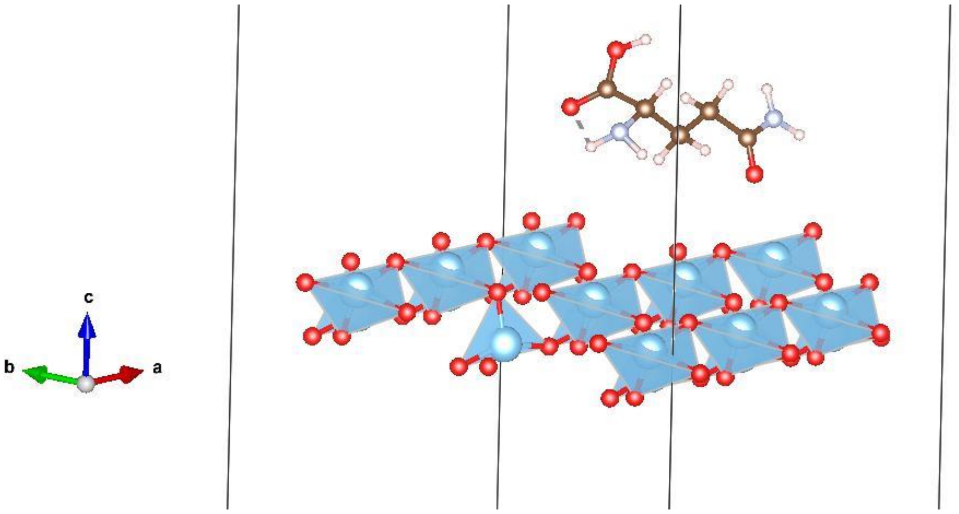

Figure S24. Optimized system Orient 10 Glutamine (L) molecule conformation on pristine 101 anatase slab surface (Minimum 1).

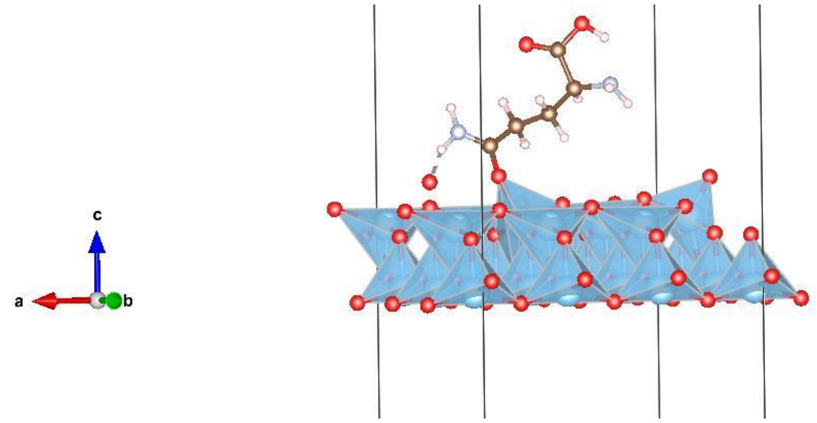

Figure S25. Optimized system Orient 1 Glutamine (L) molecule conformation on pristine 001 anatase slab surface (Minimum 2).

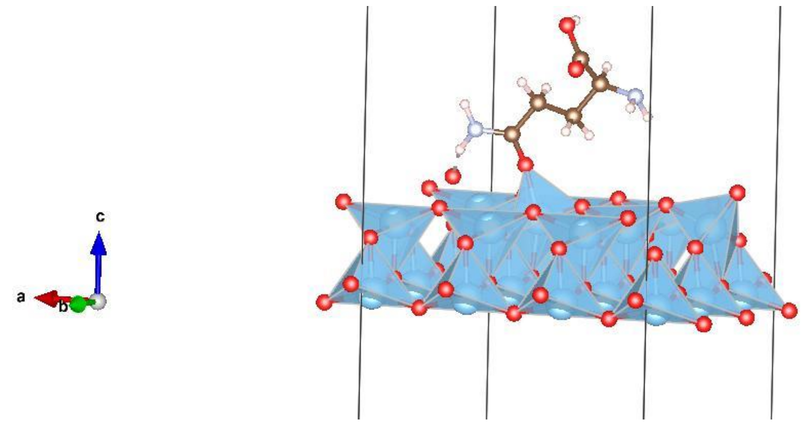

Figure S26. Optimized system Orient 1 Glutamine (L) molecule conformation on pristine 001 anatase slab surface (Minimum 3).

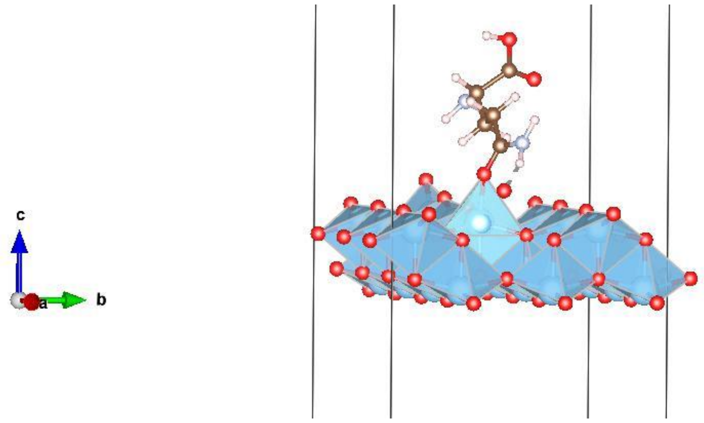

Figure S27. Optimized system Orient 1 Glutamine (L) molecule conformation on pristine 001 anatase slab surface (Minimum 4).

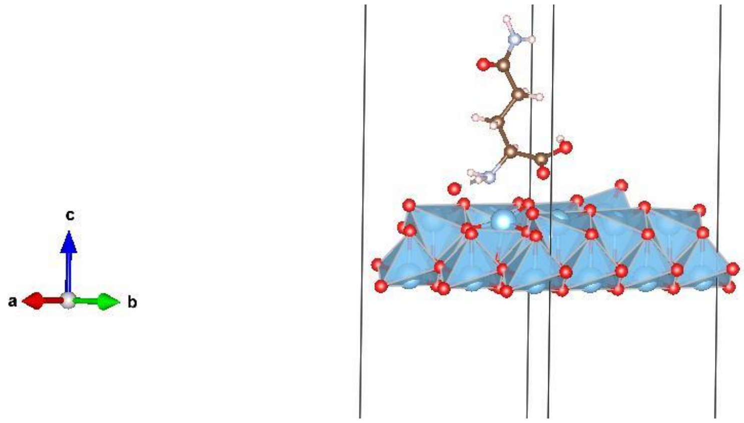

Figure S28. Optimized system Orient 3 Glutamine (L) molecule conformation on pristine 001 anatase slab surface (Minimum 2).

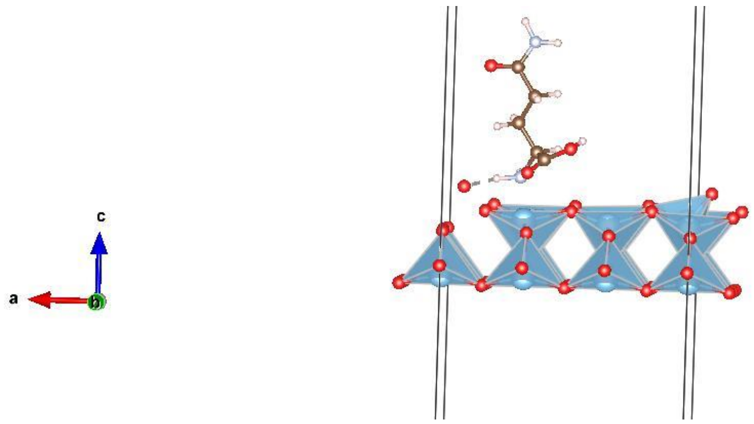

Figure S29. Optimized system Orient 3 Glutamine (L) molecule conformation on pristine 001 anatase slab surface (Minimum 3).

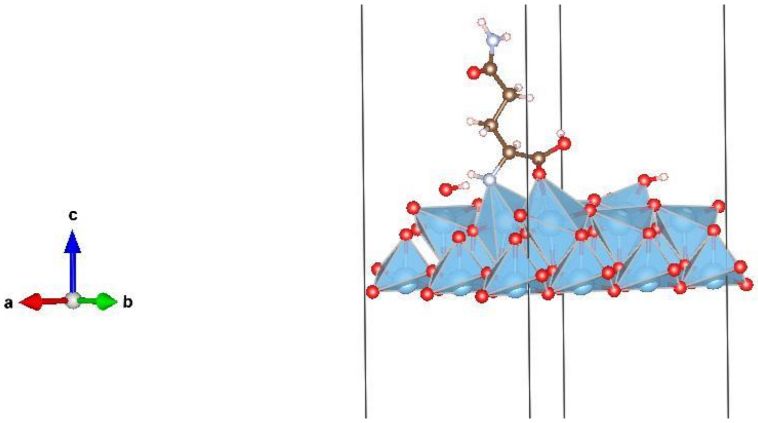

Figure S30. Optimized system Orient 3 Glutamine (L) molecule conformation on pristine 001 anatase slab surface (Minimum 4).

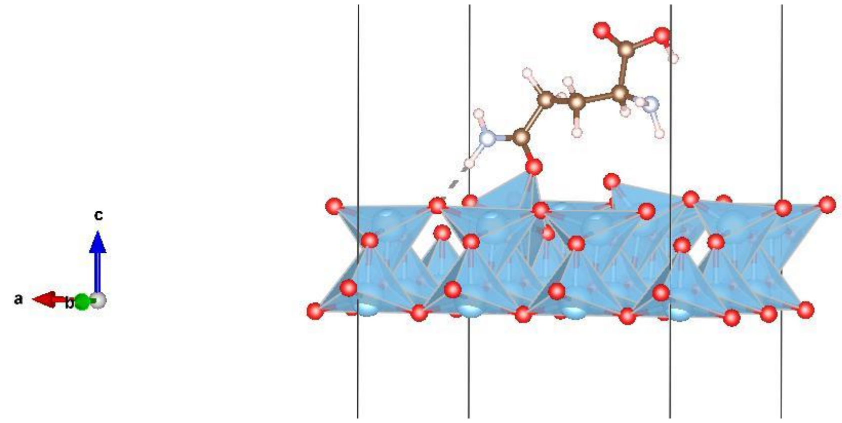

Figure S31. Optimized system Orient 4 Glutamine (L) molecule conformation on pristine 001 anatase slab surface (Minimum 2).

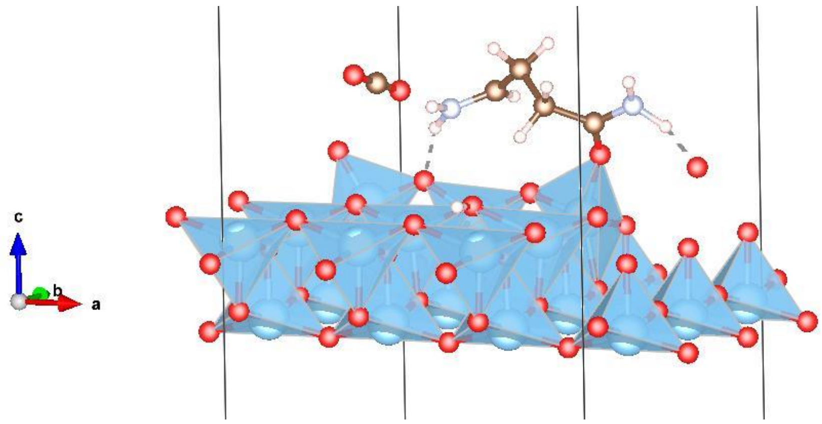

Figure S32. Optimized system Orient 4 Glutamine (L) molecule conformation on pristine 001 anatase slab surface (Minimum 3).

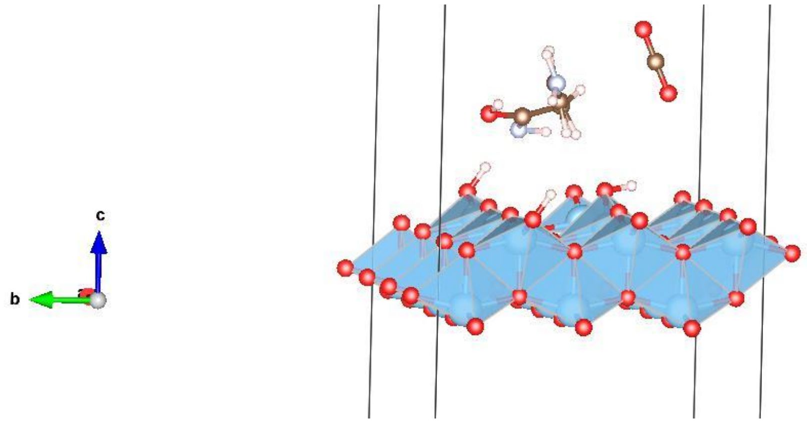

Figure S33. Optimized system Orient 4 Glutamine (L) molecule conformation on pristine 001 anatase slab surface (Minimum 4).

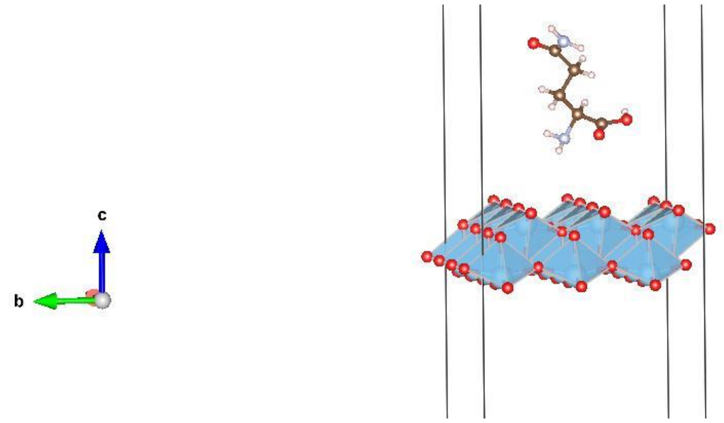

Figure S34. Optimized system Orient 6 Glutamine (L) molecule conformation on pristine 001 anatase slab surface (Minimum 2).

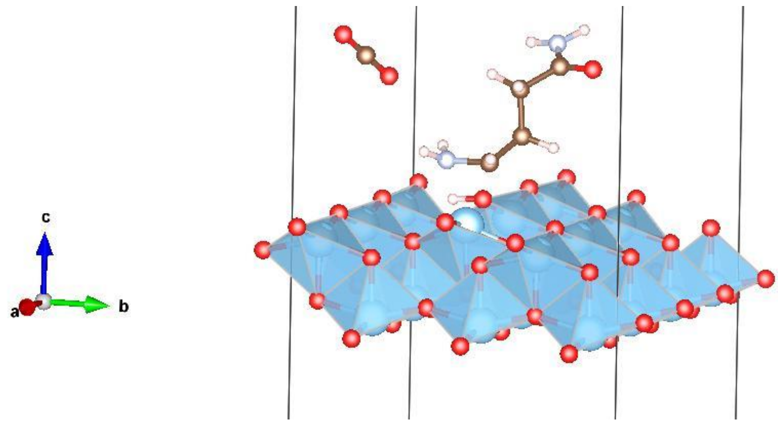

Figure S35. Optimized system Orient 6 Glutamine (L) molecule conformation on pristine 001 anatase slab surface (Minimum 3).

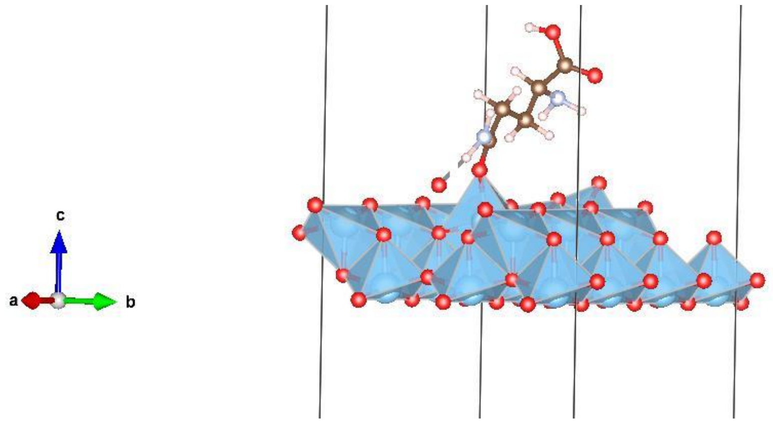

Figure S36. Optimized system Orient 6 Glutamine (L) molecule conformation on pristine 001 anatase slab surface (Minimum 4).

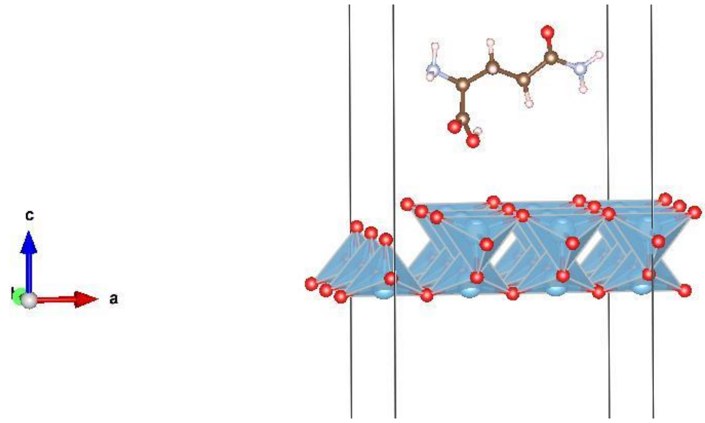

Figure S37. Optimized system Orient 7 Glutamine (L) molecule conformation on pristine 001 anatase slab surface (Minimum 2).

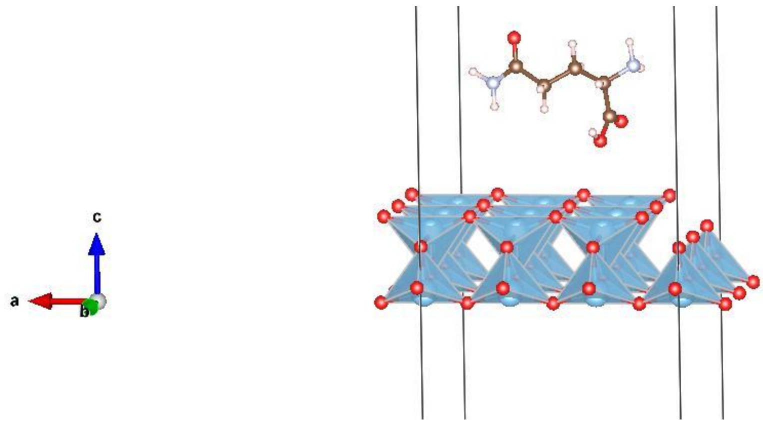

Figure S38. Optimized system Orient 7 Glutamine (L) molecule conformation on pristine 001 anatase slab surface (Minimum 3).

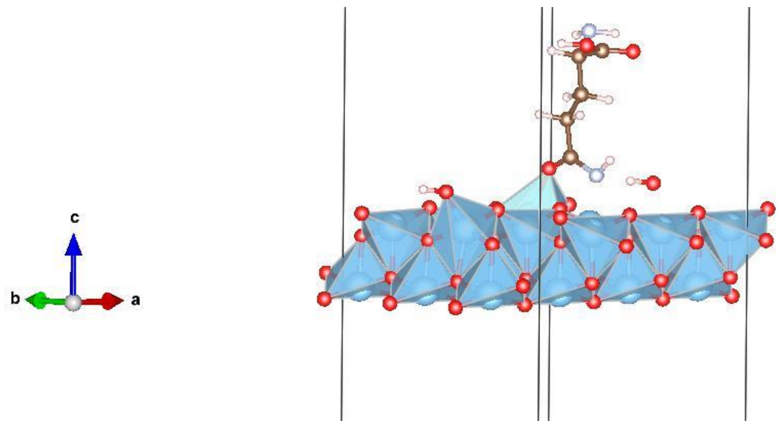

Figure S39. Optimized system Orient 7 Glutamine (L) molecule conformation on pristine 001 anatase slab surface (Minimum 4).

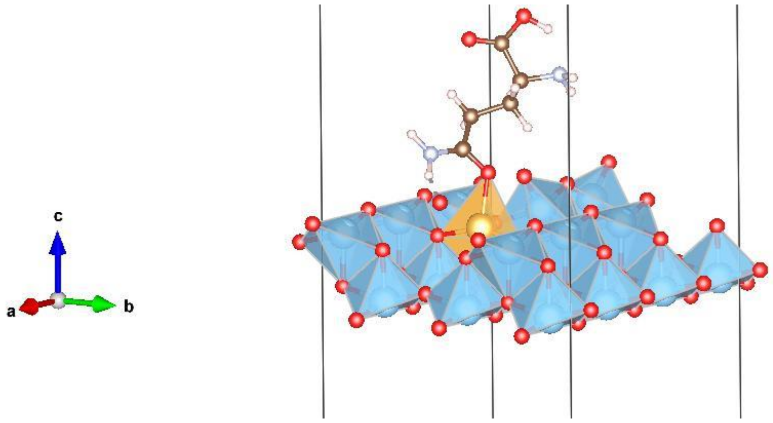

Figure S40. Optimized system Orient 1 Glutamine (L) molecule conformation on Au-doped 001 anatase slab surface.

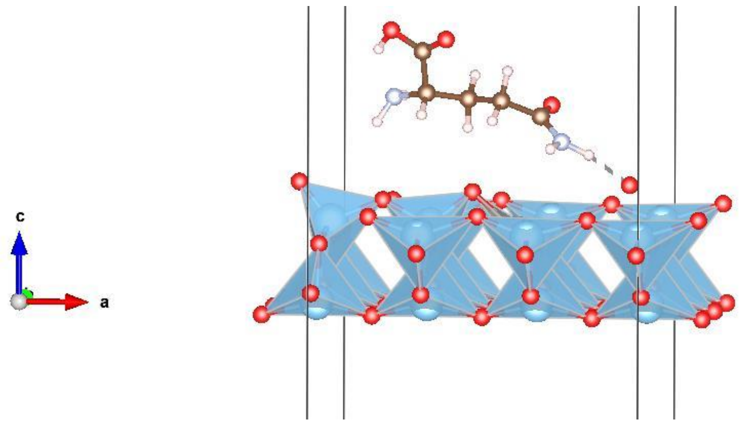

Figure S41. Optimized system Orient 1 Glutamine (L) molecule conformation on Ag-doped 001 anatase slab surface.

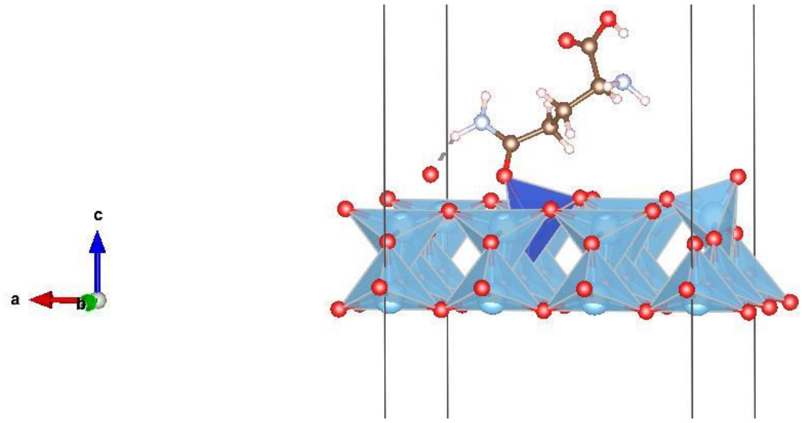

Figure S42. Optimized system Orient 1 Glutamine (L) molecule conformation on Cu-doped 001 anatase slab surface.

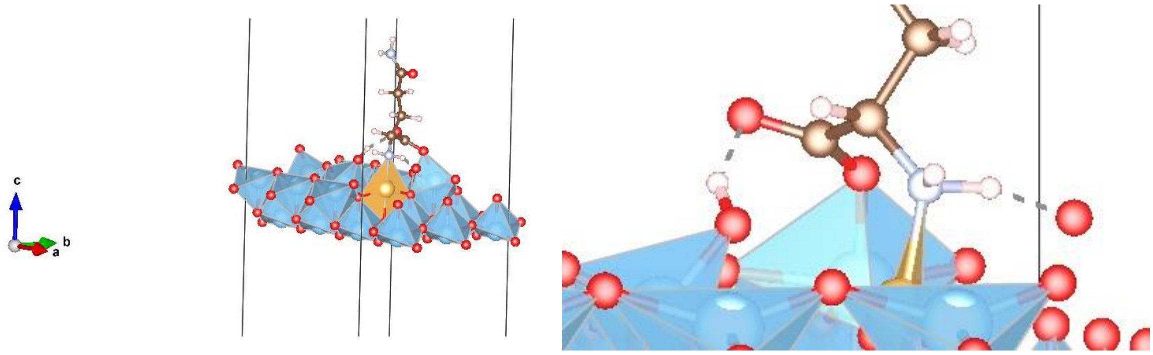

Figure S43. Optimized system Orient 3 Glutamine (L) molecule conformation on Au-doped 001 anatase slab surface (left), with the zoomed interaction (right).

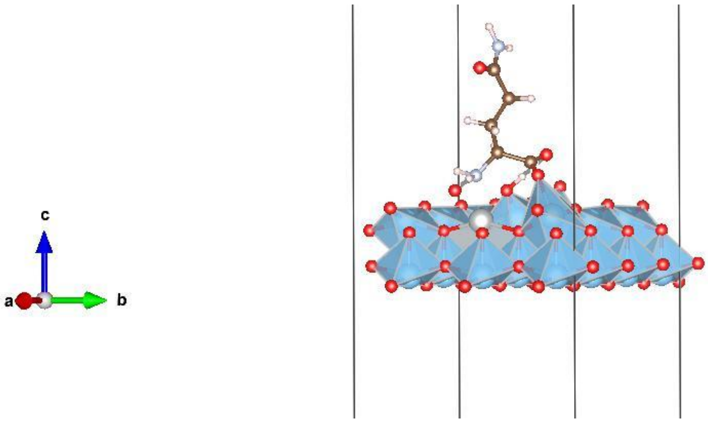

Figure S44. Optimized system Orient 3 Glutamine (L) molecule conformation on Ag-doped 001 anatase slab surface.

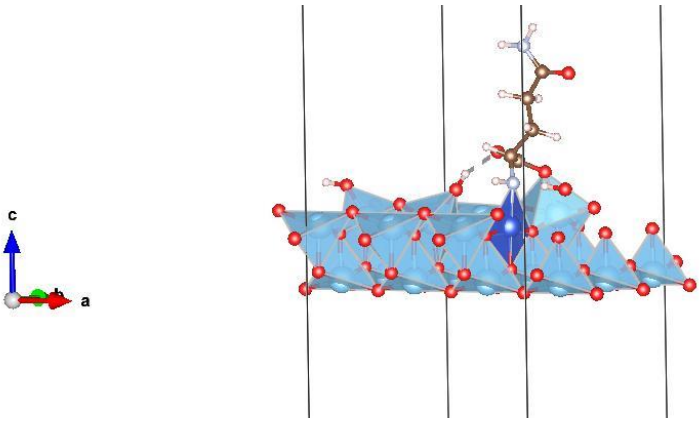

Figure S45. Optimized system Orient 3 Glutamine (L) molecule conformation on Cu-doped 001 anatase slab surface.

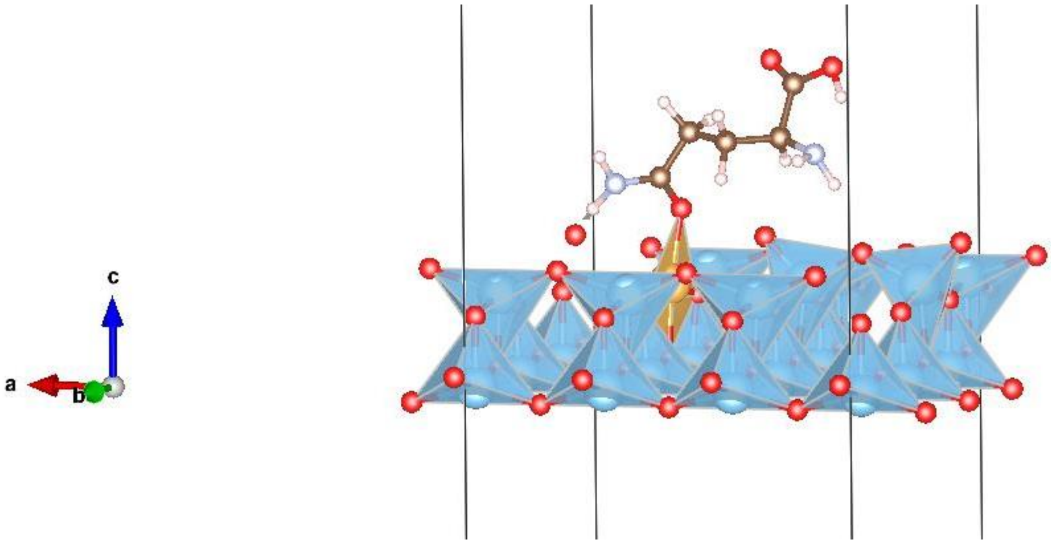

Figure S46. Optimized system Orient 4 Glutamine (L) molecule conformation on Au-doped 001 anatase slab surface.

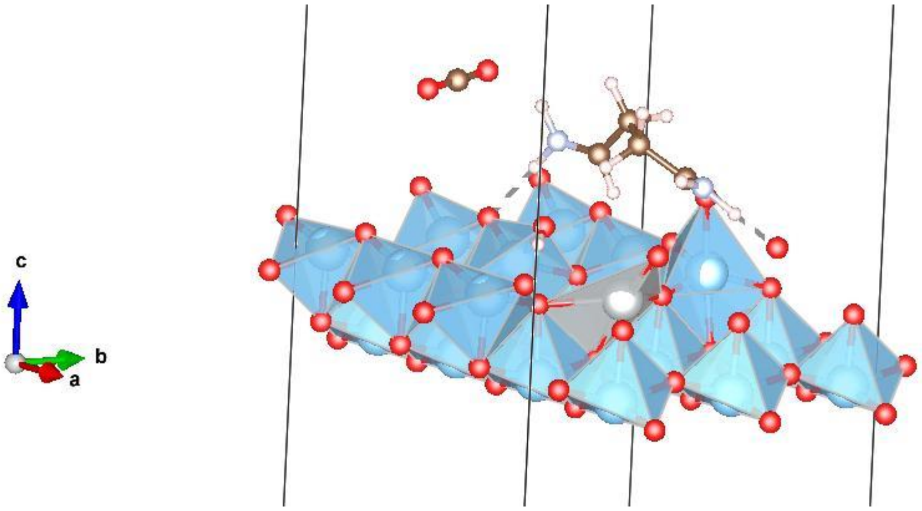

Figure S47. Optimized system Orient 4 Glutamine (L) molecule conformation on Ag-doped 001 anatase slab surface.

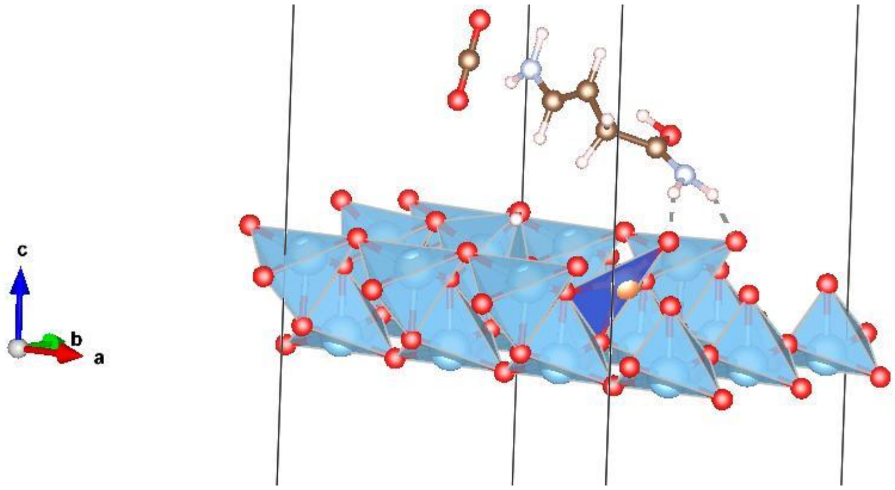

Figure S48. Optimized system Orient 4 Glutamine (L) molecule conformation on Cu-doped 001 anatase slab surface.

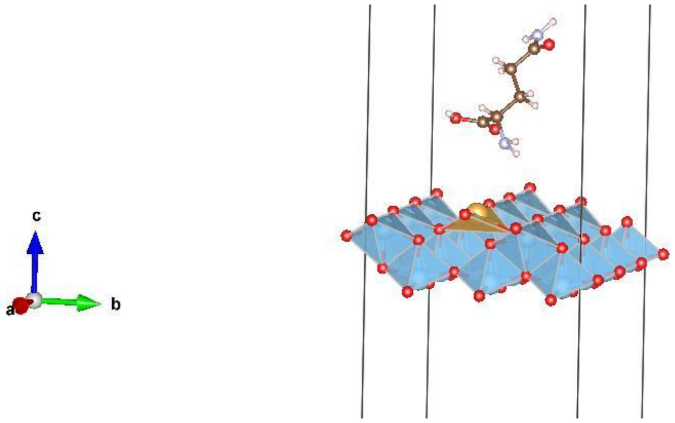

Figure S49. Optimized system Orient 6 Glutamine (L) molecule conformation on Au-doped 001 anatase slab surface.

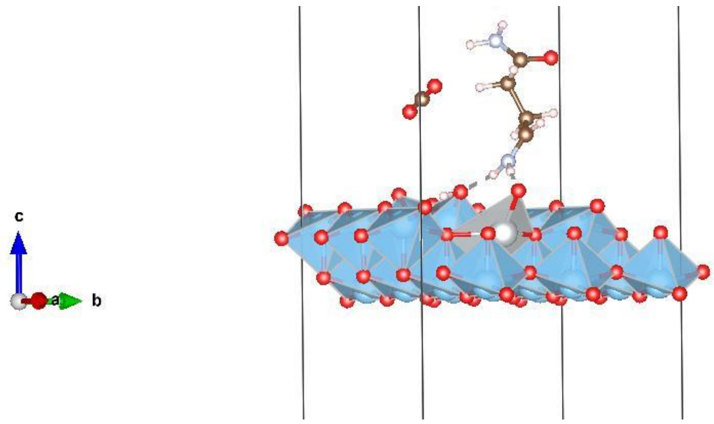

Figure S50. Optimized system Orient 6 Glutamine (L) molecule conformation on Ag-doped 001 anatase slab surface.

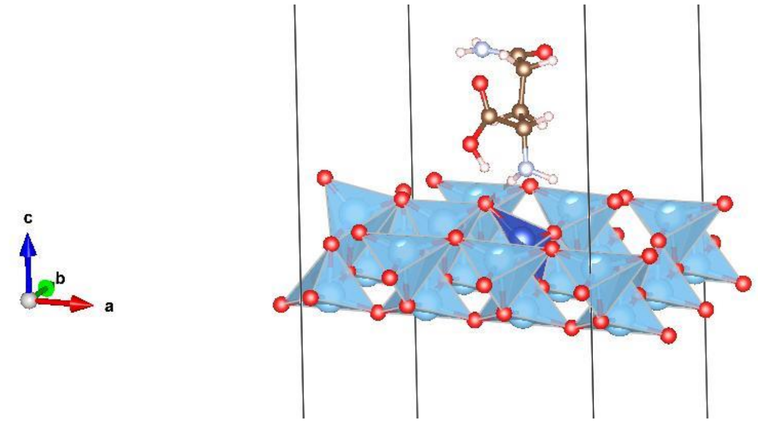

Figure S51. Optimized system Orient 6 Glutamine (L) molecule conformation on Cu-doped 001 anatase slab surface.

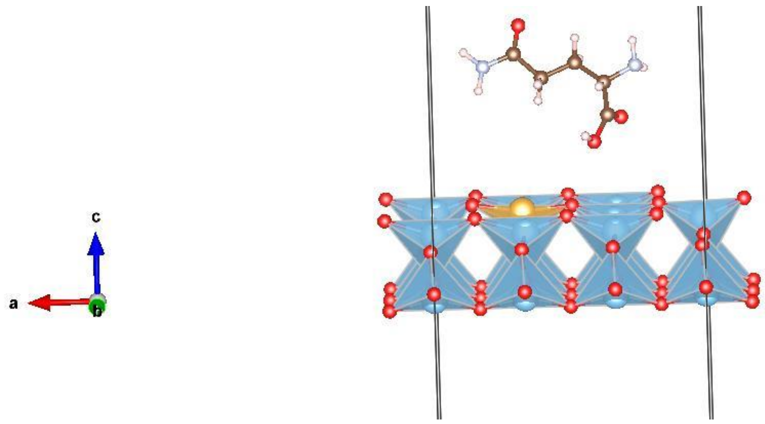

Figure S52. Optimized system Orient 7 Glutamine (L) molecule conformation on Au-doped 001 anatase slab surface.

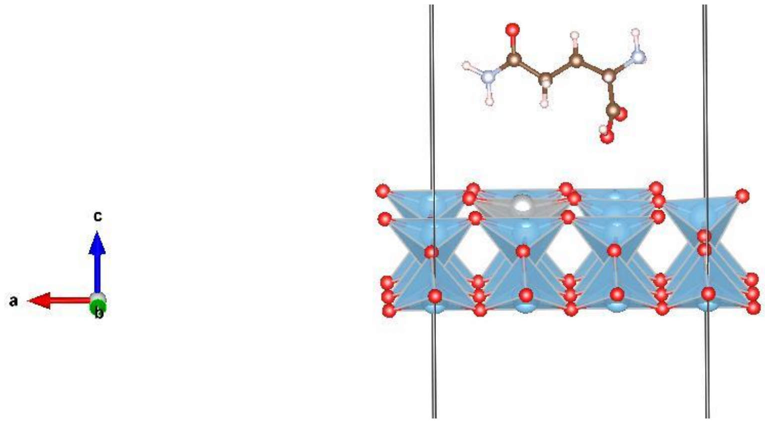

Figure S53. Optimized system Orient 7 Glutamine (L) molecule conformation on Ag-doped 001 anatase slab surface.

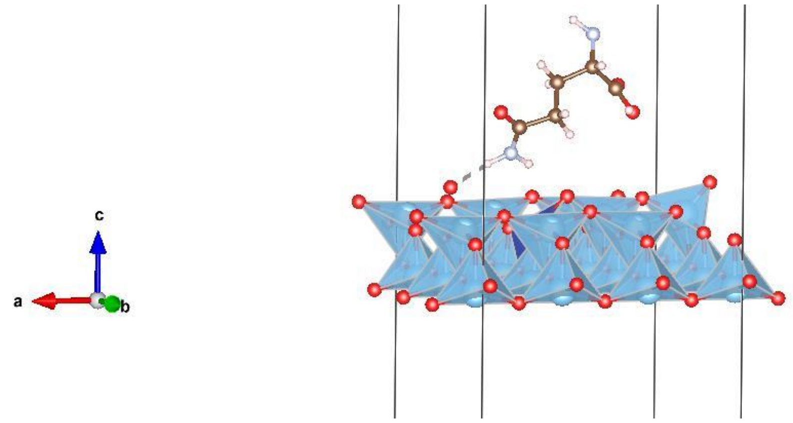

Figure S54. Optimized system Orient 7 Glutamine (L) molecule conformation on Cu-doped 001 anatase slab surface.

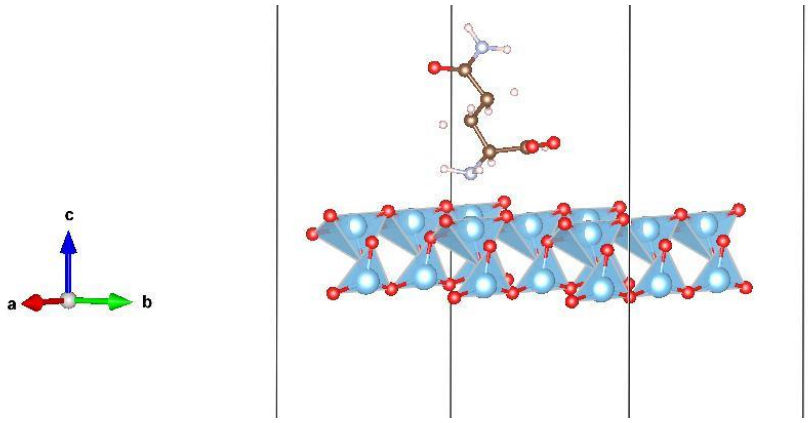

Figure S55. Optimized system Orient 3 Glutamine (L) molecule conformation on pristine 101 anatase slab surface (Minimum 2).

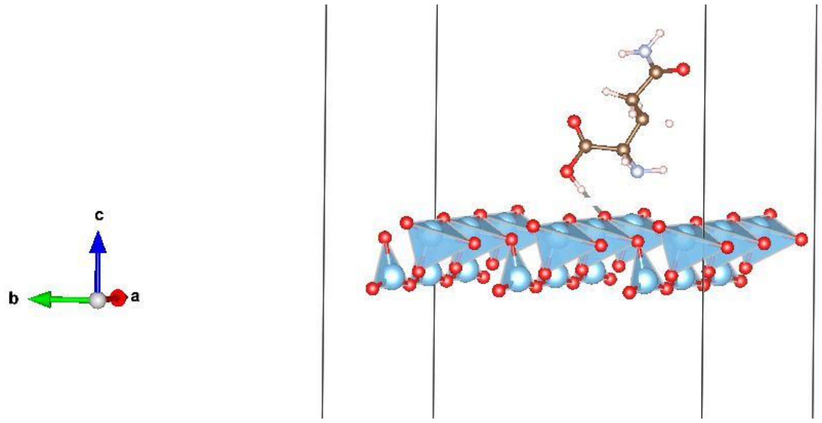

Figure S56. Optimized system Orient 3 Glutamine (L) molecule conformation on pristine 101 anatase slab surface (Minimum 3).

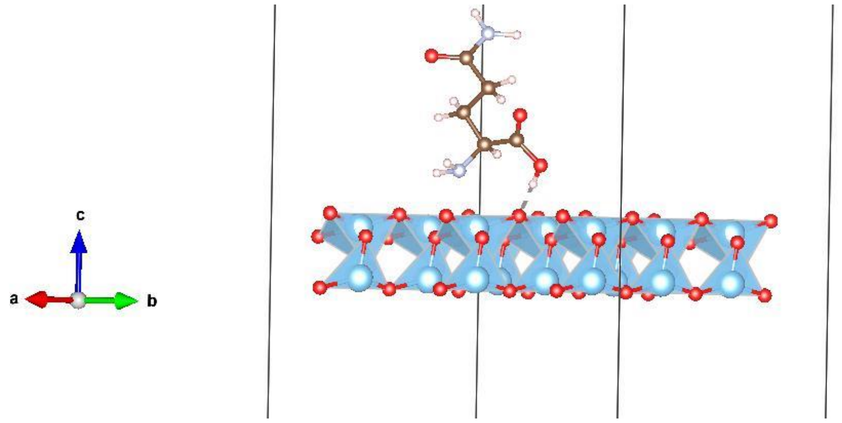

Figure S57. Optimized system Orient 3 Glutamine (L) molecule conformation on pristine 101 anatase slab surface (Minimum 4).

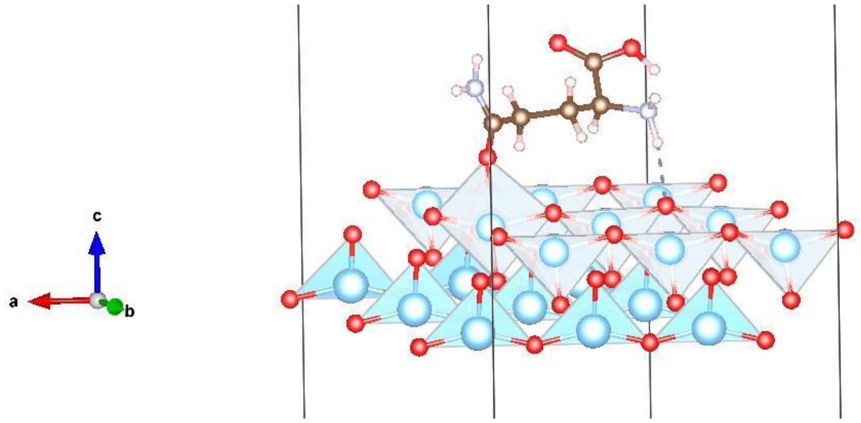

Figure S58. Optimized system Orient 4 Glutamine (L) molecule conformation on pristine 101 anatase slab surface (Minimum 2).

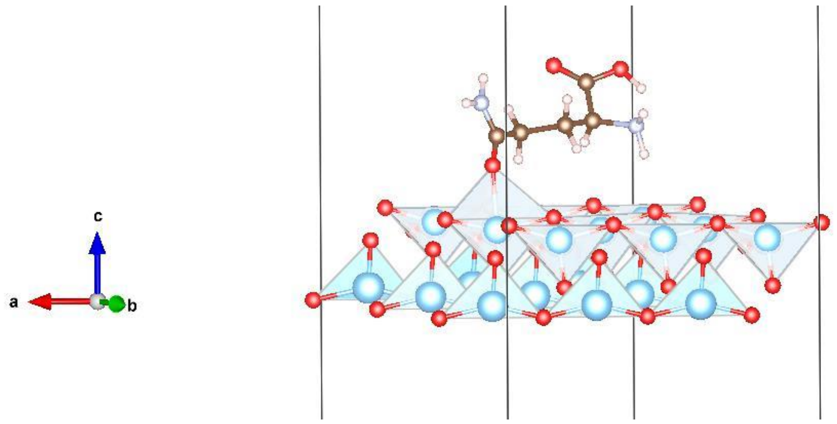

Figure S59. Optimized system Orient 4 Glutamine (L) molecule conformation on pristine 101 anatase slab surface (Minimum 3).

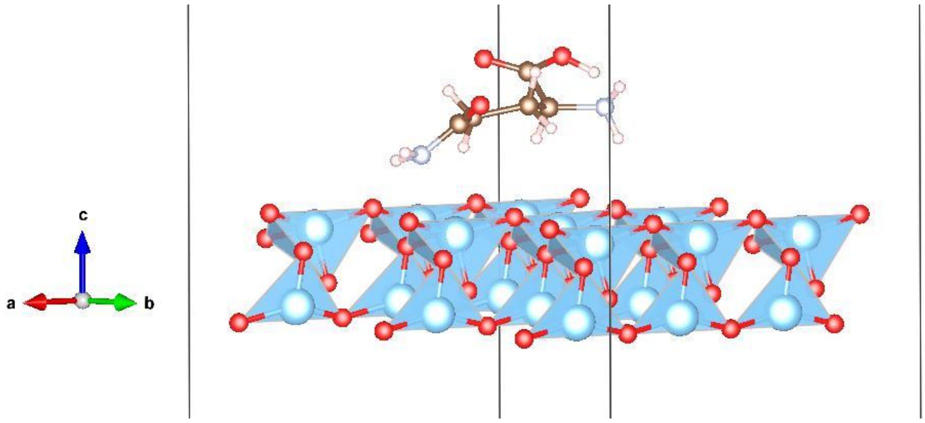

Figure S60. Optimized system Orient 4 Glutamine (L) molecule conformation on pristine 101 anatase slab surface (Minimum 4).

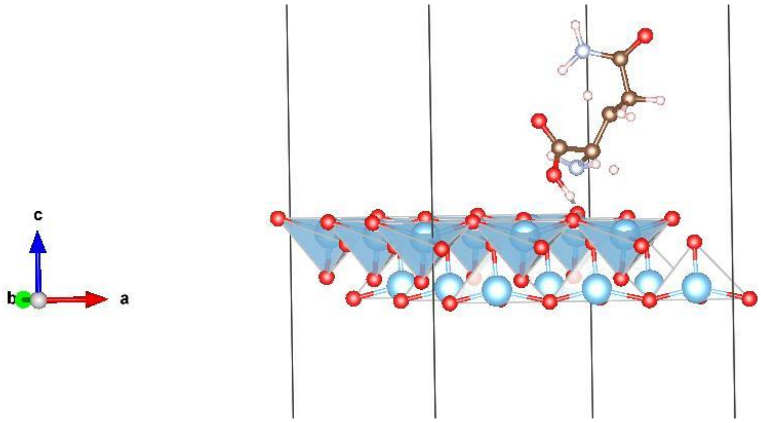

Figure S61. Optimized system Orient 6 Glutamine (L) molecule conformation on pristine 101 anatase slab surface (Minimum 2).

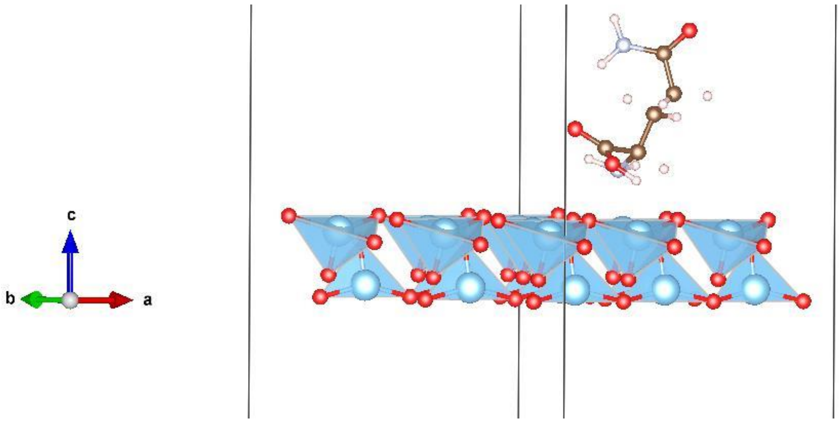

Figure S62. Optimized system Orient 6 Glutamine (L) molecule conformation on pristine 101 anatase slab surface (Minimum 3).

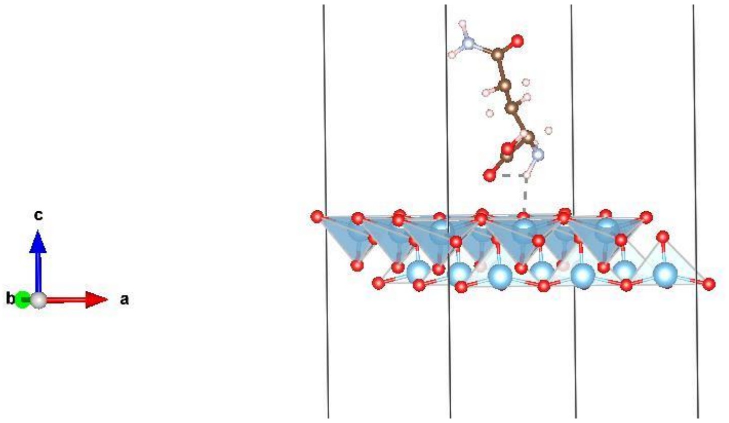

Figure S63. Optimized system Orient 6 Glutamine (L) molecule conformation on pristine 101 anatase slab surface (Minimum 4).

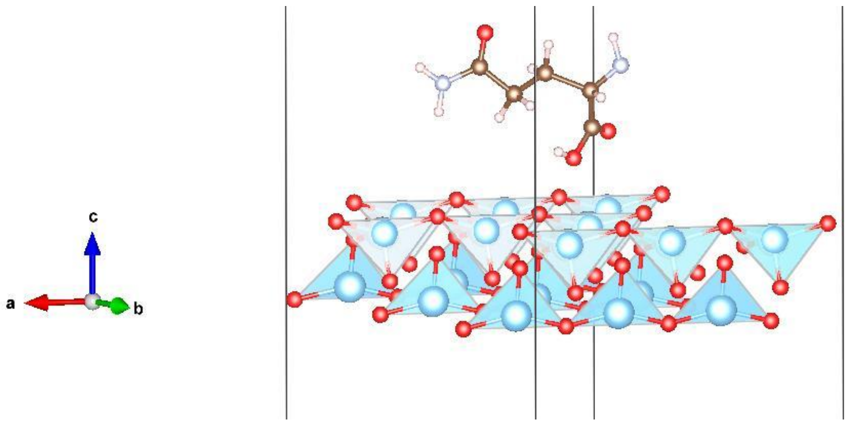

Figure S64. Optimized system Orient 7 Glutamine (L) molecule conformation on pristine 101 anatase slab surface (Minimum 2).

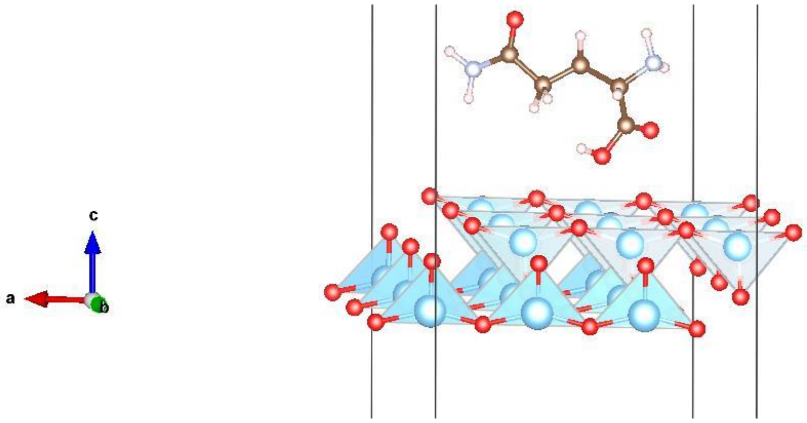

Figure S65. Optimized system Orient 7 Glutamine (L) molecule conformation on pristine 101 anatase slab surface (Minimum 3).

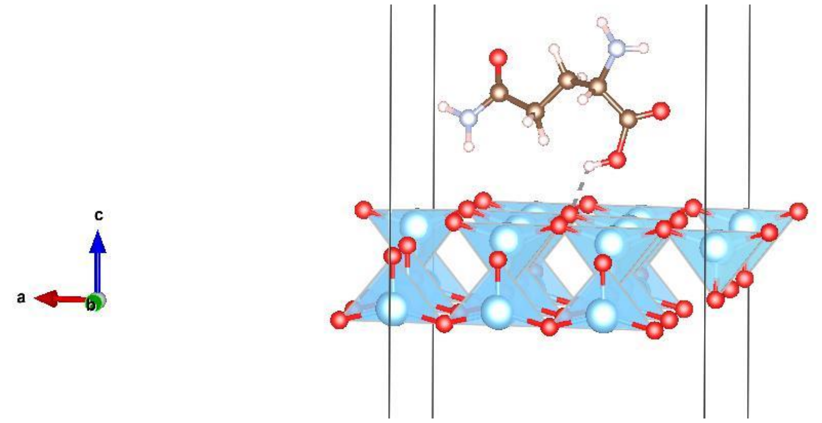

Figure S66. Optimized system Orient 7 Glutamine (L) molecule conformation on pristine 101 anatase slab surface (Minimum 4).

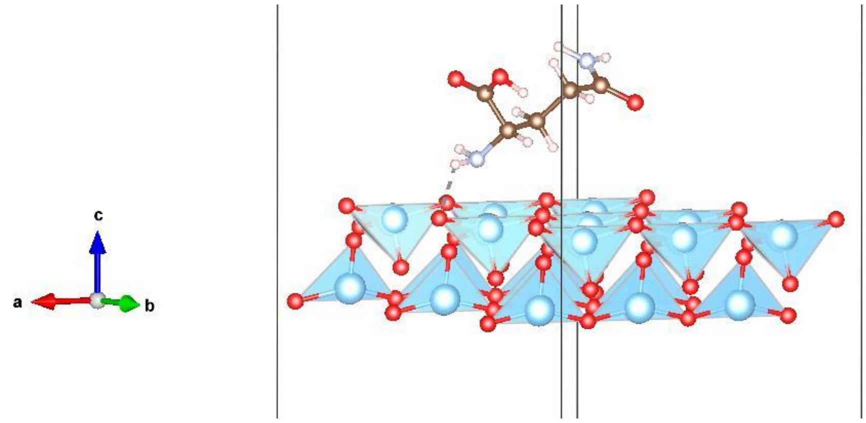

Figure S67. Optimized system Orient 9 Glutamine (L) molecule conformation on pristine 101 anatase slab surface (Minimum 2).

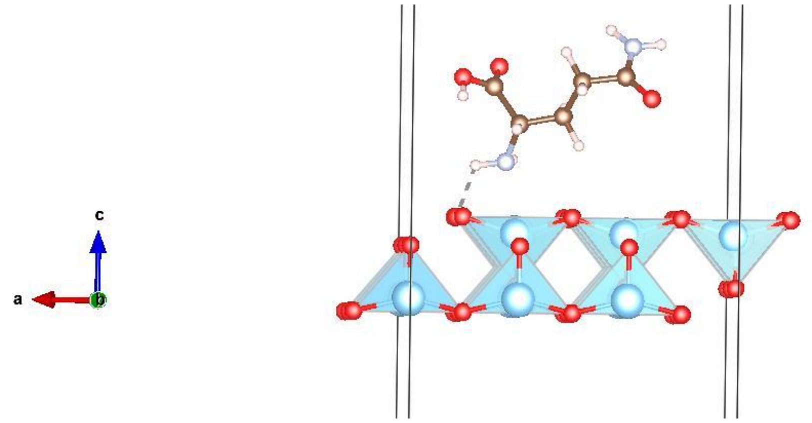

Figure S68. Optimized system Orient 9 Glutamine (L) molecule conformation on pristine 101 anatase slab surface (Minimum 3).

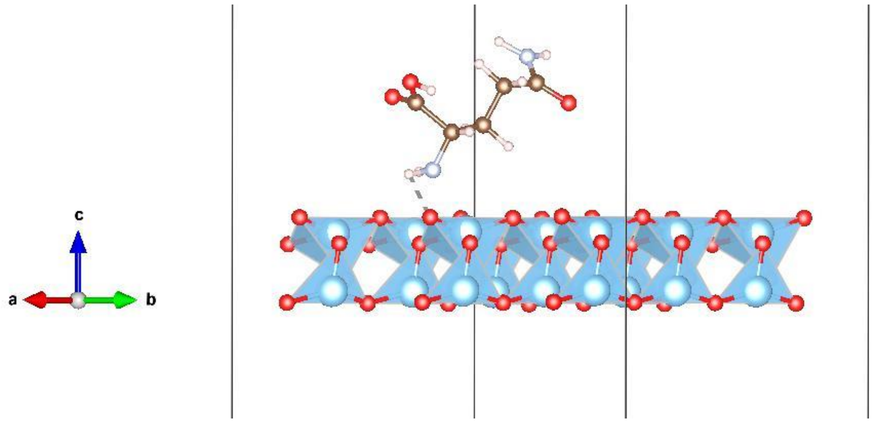

Figure S69. Optimized system Orient 9 Glutamine (L) molecule conformation on pristine 101 anatase slab surface (Minimum 4).

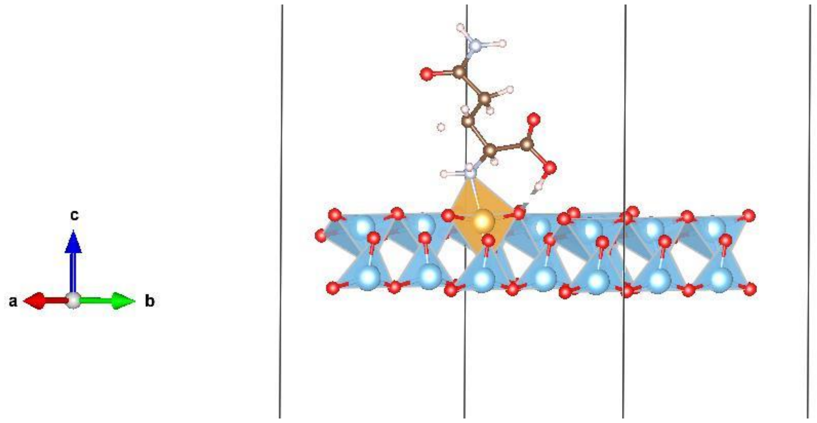

Figure S70. Optimized system Orient 3 Glutamine (L) molecule conformation on Au-doped 101 anatase slab surface.

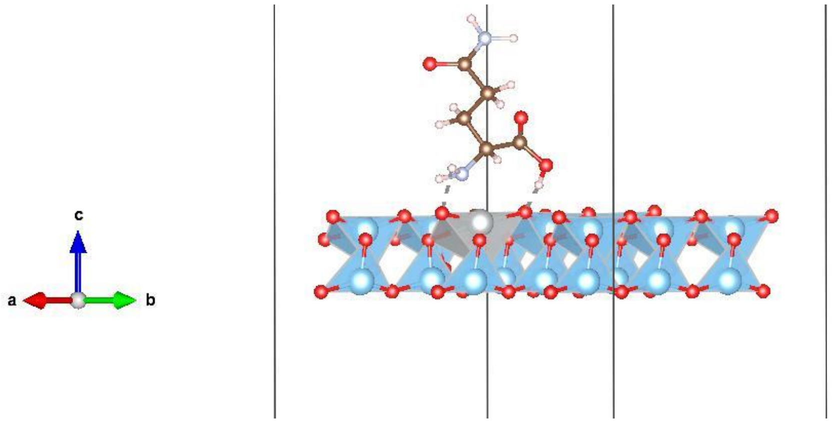

Figure S71. Optimized system Orient 3 Glutamine (L) molecule conformation on Ag-doped 101 anatase slab surface.

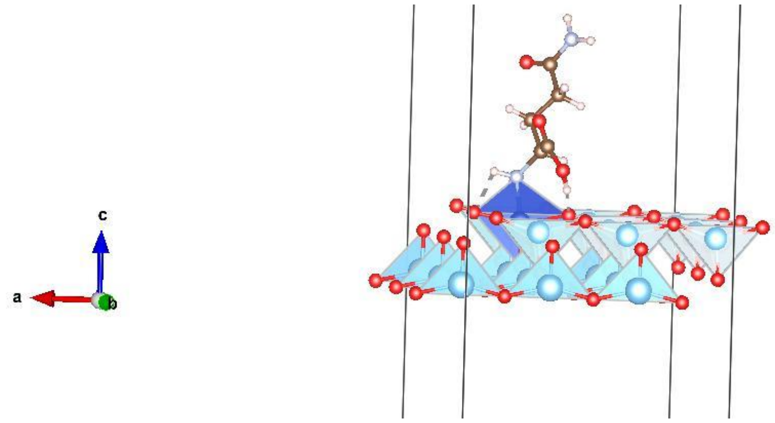

Figure S72. Optimized system Orient 3 Glutamine (L) molecule conformation on Cu-doped 101 anatase slab surface.

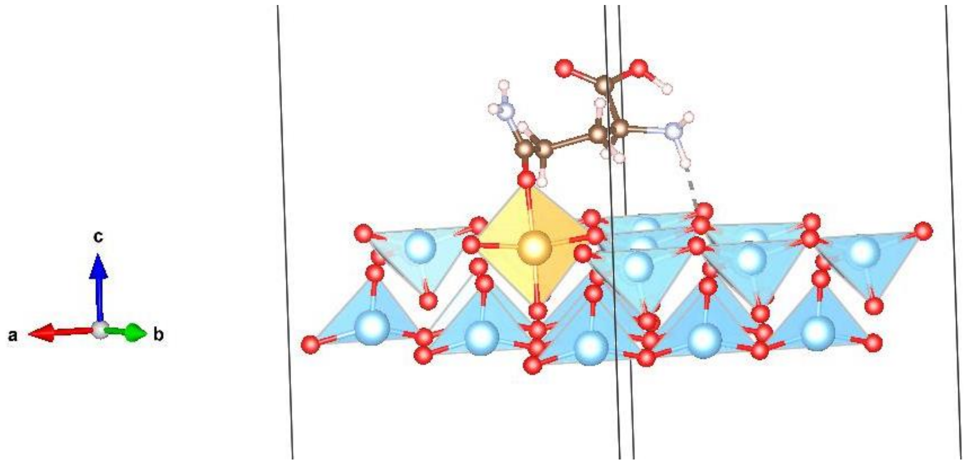

Figure S73. O Optimized system Orient 4 Glutamine (L) molecule conformation on Au-doped 101 anatase slab surface.

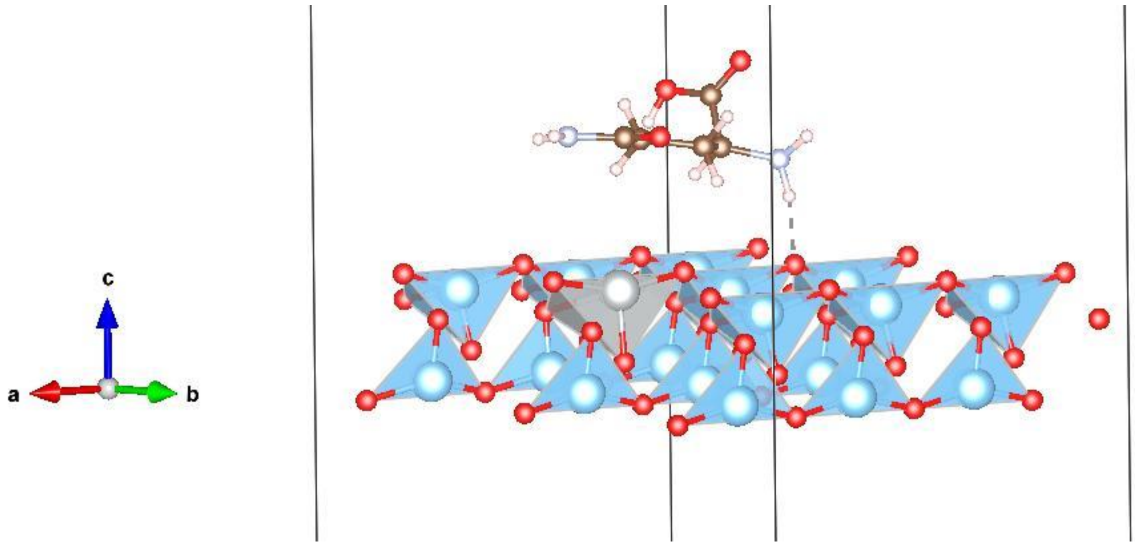

Figure S74. Optimized system Orient 4 Glutamine (L) molecule conformation on Ag-doped 101 anatase slab surface.

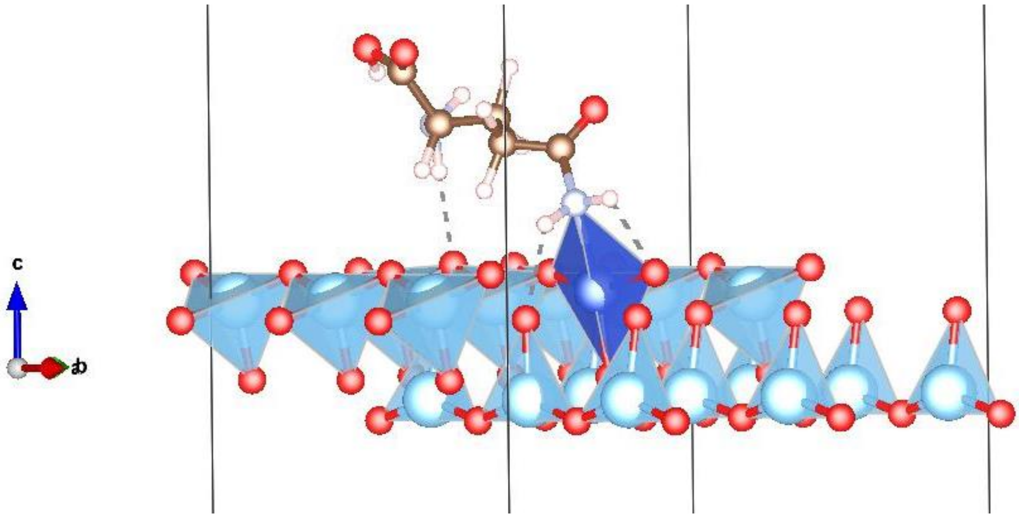

Figure S75. Optimized system Orient 4 Glutamine (L) molecule conformation on Cu-doped 101 anatase slab surface.

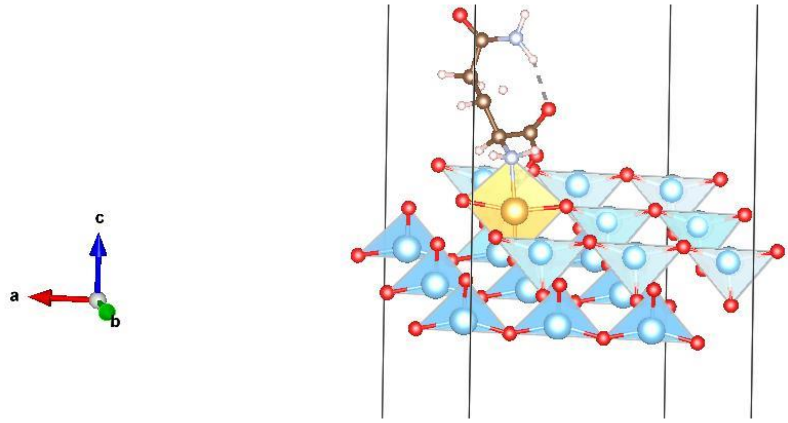

Figure S76. Optimized system Orient 6 Glutamine (L) molecule conformation on Au-doped 101 anatase slab surface.

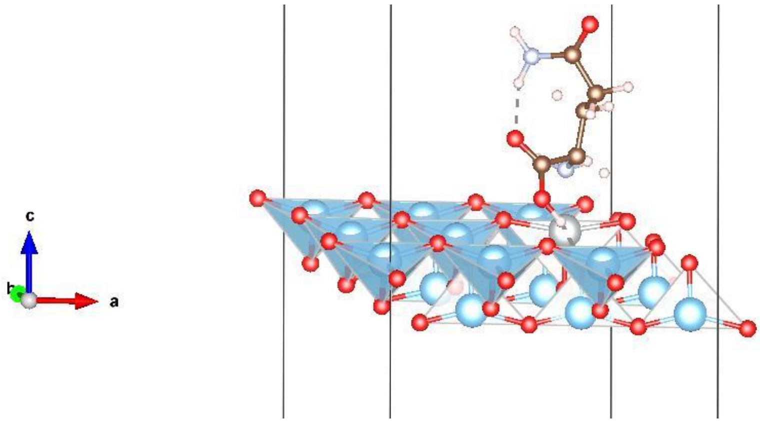

Figure S77. Optimized system Orient 6 Glutamine (L) molecule conformation on Ag-doped 101 anatase slab surface.

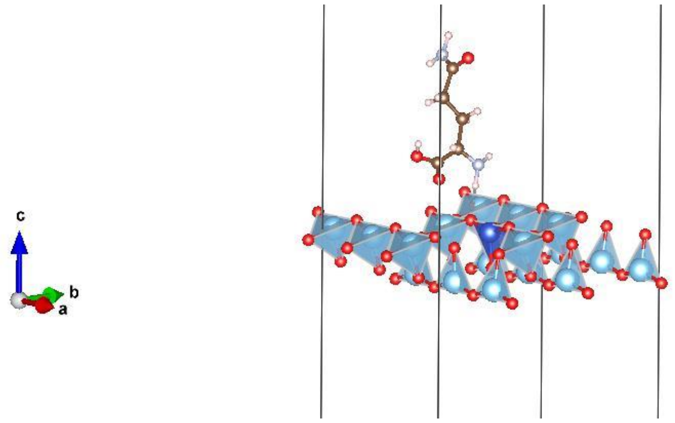

Figure S78. Optimized system Orient 6 Glutamine (L) molecule conformation on Cu-doped 101 anatase slab surface.

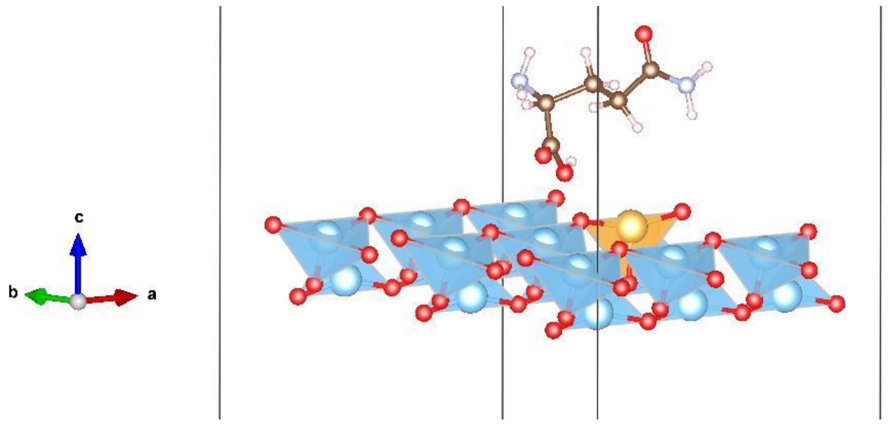

Figure S79. Optimized system Orient 7 Glutamine (L) molecule conformation on Au-doped 101 anatase slab surface.

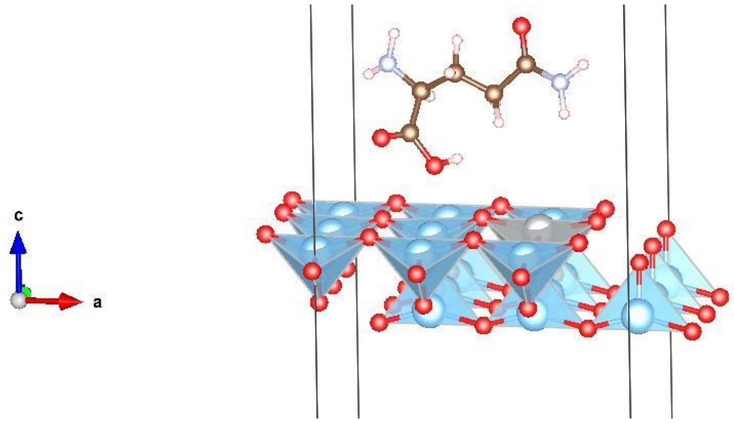

Figure S80. Optimized system Orient 7 Glutamine (L) molecule conformation on Ag-doped 101 anatase slab surface.

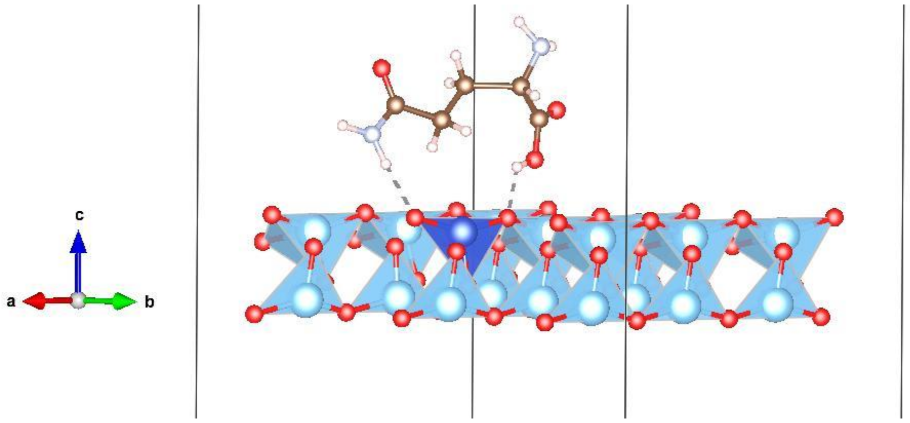

Figure S81. Optimized system Orient 7 Glutamine (L) molecule conformation on Cu-doped 101 anatase slab surface.

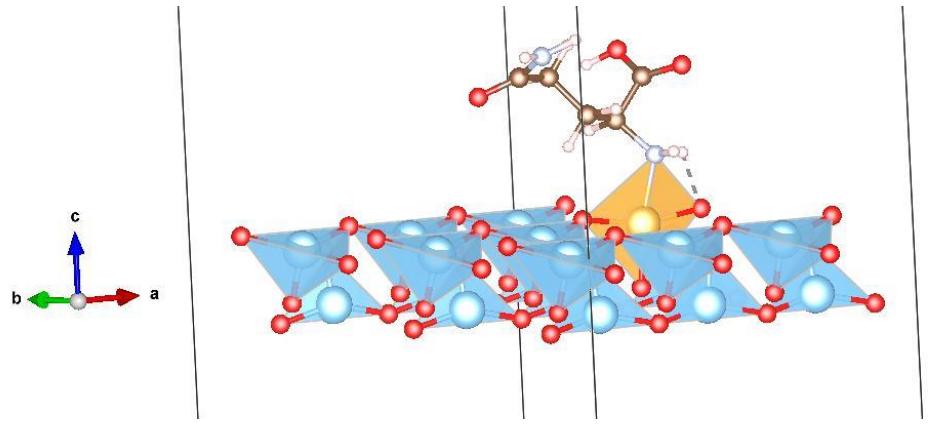

Figure S82. Optimized system Orient 9 Glutamine (L) molecule conformation on Au-doped 101 anatase slab surface.

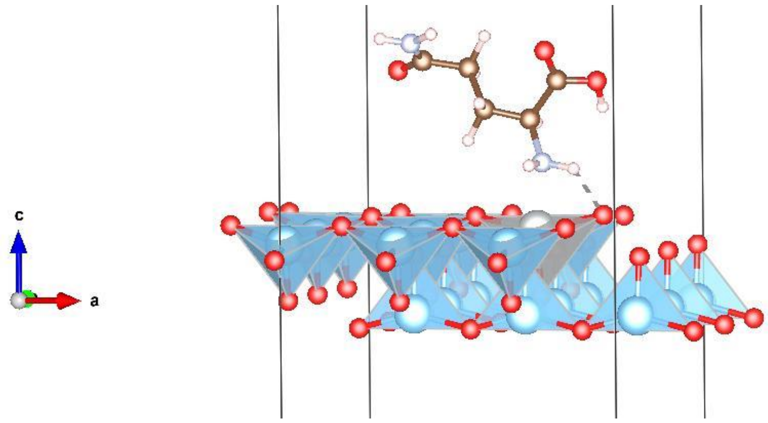

Figure S83. Optimized system Orient 9 Glutamine (L) molecule conformation on Ag-doped 101 anatase slab surface.

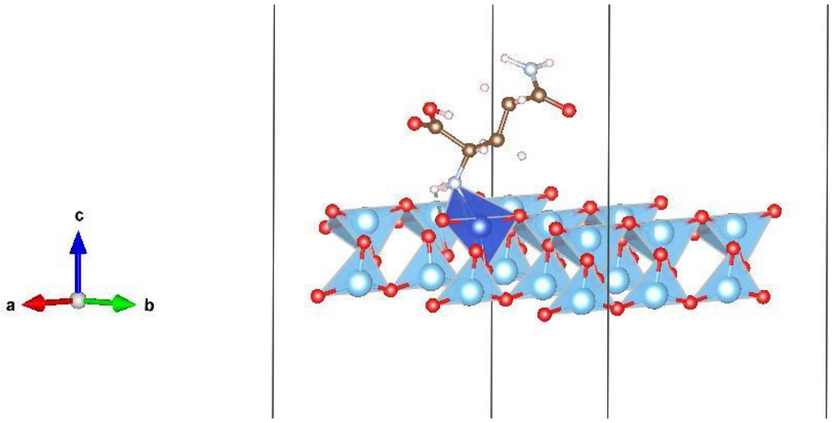

Figure S84. Optimized system Orient 9 Glutamine (L) molecule conformation on Cu-doped 101 anatase slab surface.

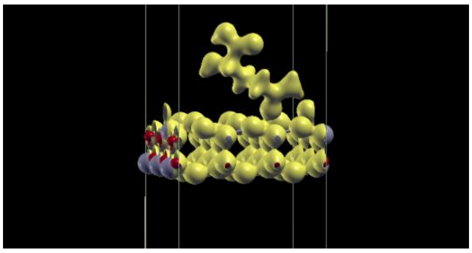

a)

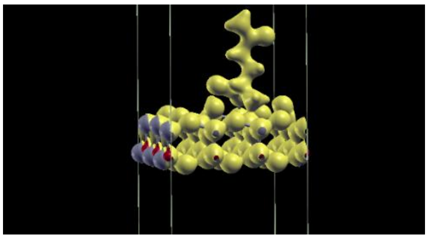

b)

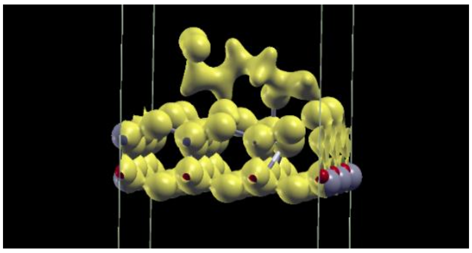

c)

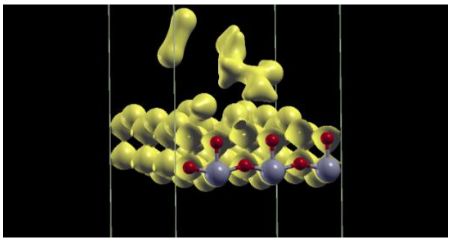

d)

Figure S85. Electron Density visualizations of some of the best energy candidates of system Glutamine (L) on 001 pristine and Au / Ag / Cu -doped 001 anatase slab surfaces calculated by Quantum Espresso code and visualized by XCrysDen program: **a**) Orient 1-pristine; **b**) Orient 3-Au-doped; **c**) Orient 4-Ag-doped; and **d**) Orient 4-Cu-doped.

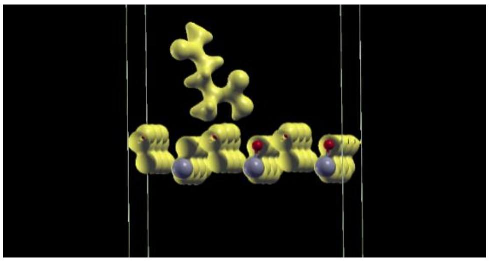

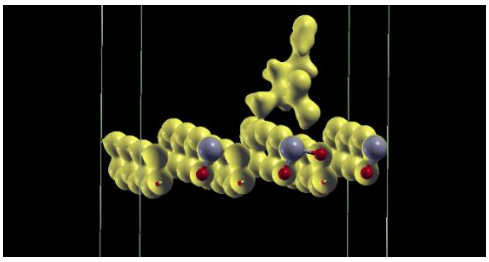

a) 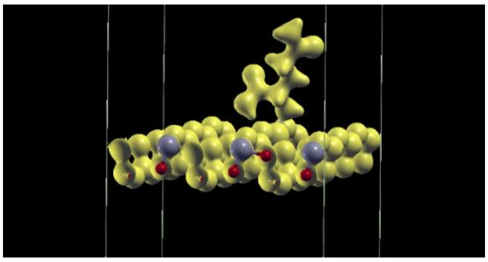

b) 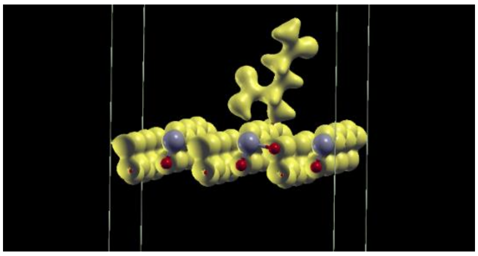

c)

d)

Figure S86. Electron Density visualizations of some of the best energy candidates of system Glutamine (L) on 101 pristine and Au / Ag / Cu -doped 001 anatase slab surfaces calculated by Quantum Espresso code and visualized by XCrysDen program: **a**) Orient 3 - pristine; **b**) Orient 6-Au-doped; **c**) Orient 3-Ag-doped; and **d**) Orient 3-Cu-doped.

**System**	**Minimum 1**	**Minimum 2**	**Minimum 3**	**Minimum 4**
**Orient 1**	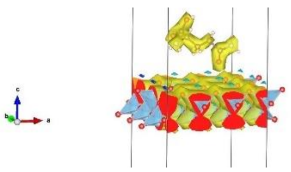	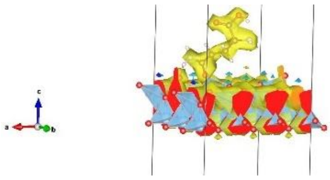	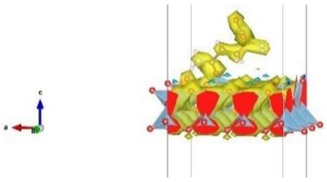	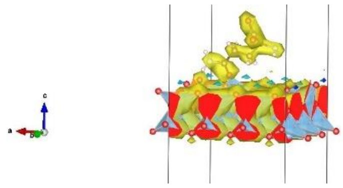
**Orient 3**	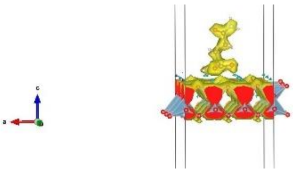	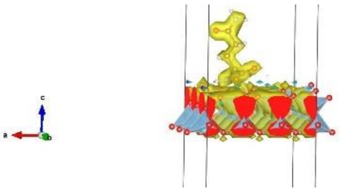	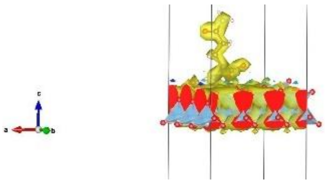	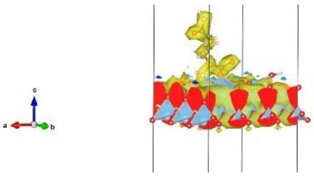
**Orient 4**	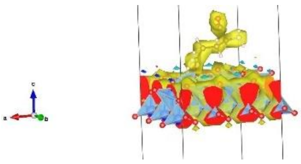	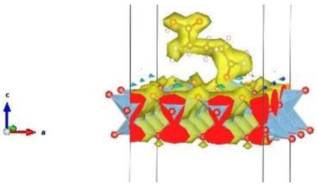	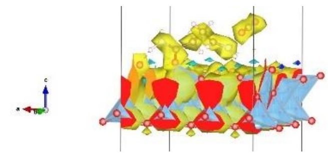	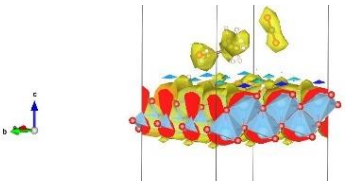
**Orient 6**	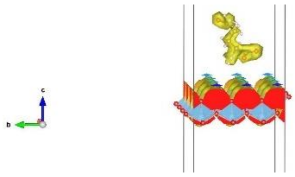	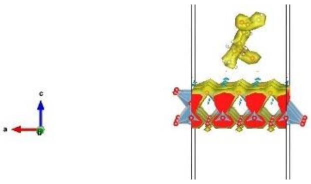	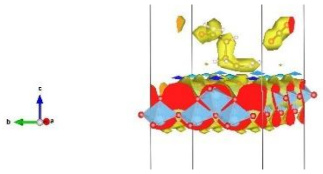	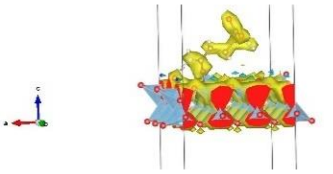

**Orient 7**	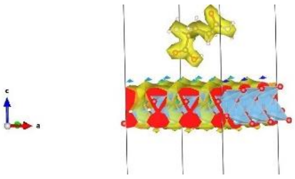	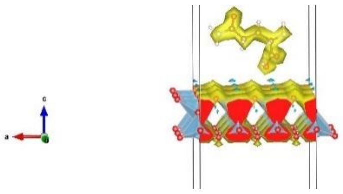	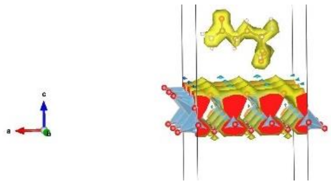	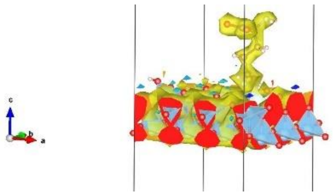

Figure S87. Visualized electron densities of various Glutamine (L) molecule conformations (Orient 1, 3, 4, 6, and 7) on pristine 001 anatase surfaces.

System	Au-doped	Ag-doped	Cu-doped
**Orient 1**	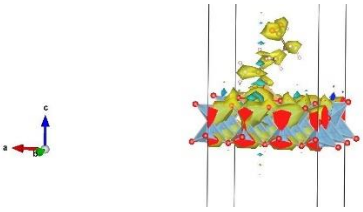	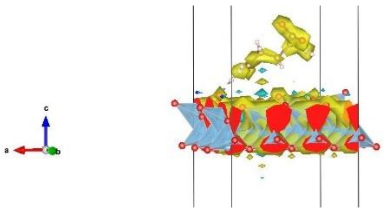	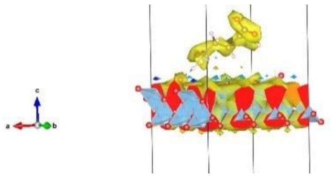
**Orient 3**	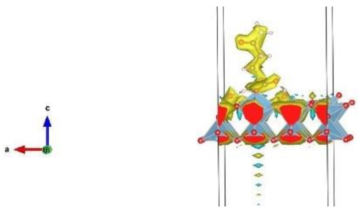	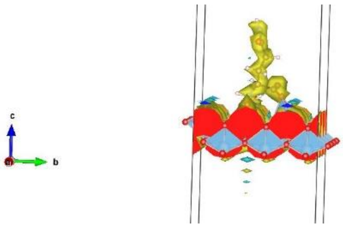	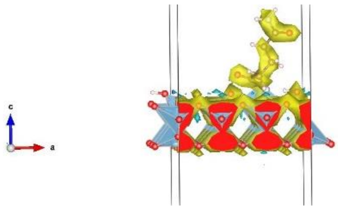
**Orient 4**	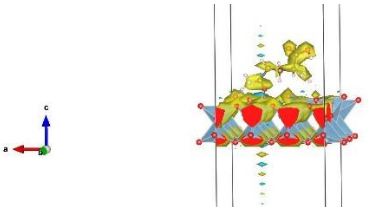	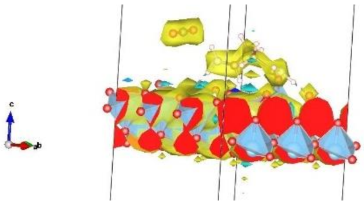	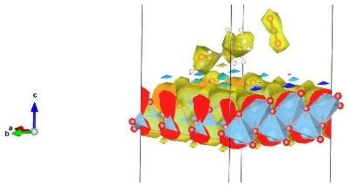
**Orient 6**	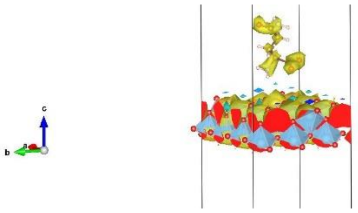	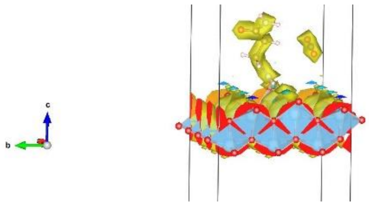	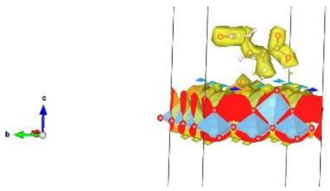
**Orient 7**	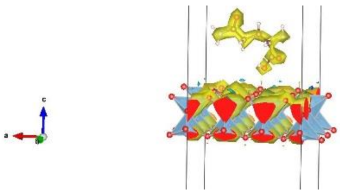	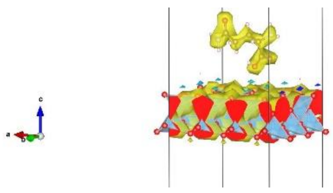	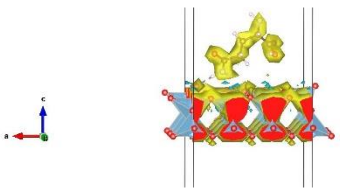

**Orient 1a***	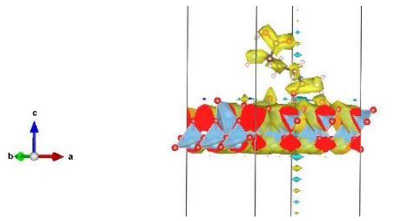	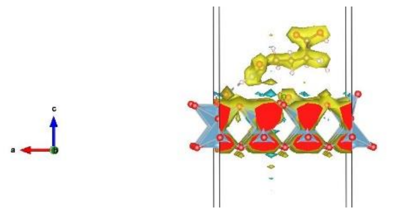	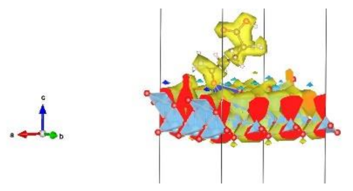

Figure S88. Visualized electron densities of various Glutamine (L) molecule conformations (Orient 1, 3, 4, 6, and 7) on Au / Ag / Cu -doped 001 anatase surfaces.

System	Pristine	Au-un-doped	Ag-un-doped	Cu-un-doped
**Orient 3**	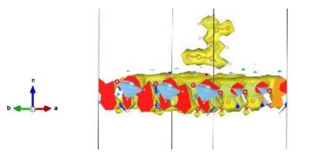	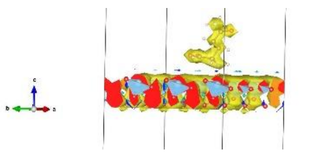	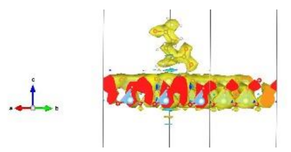	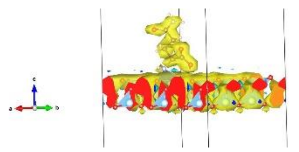
**Orient 4**	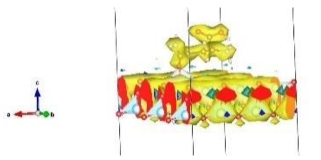	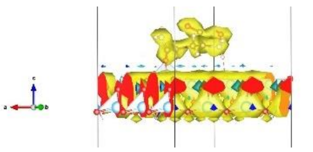	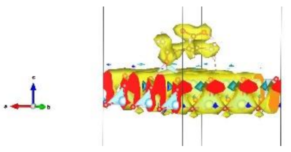	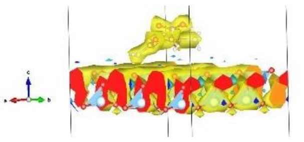
**Orient 6**	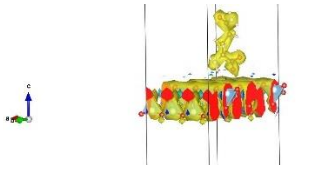	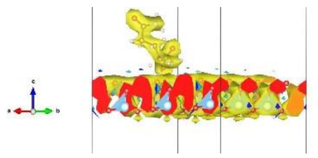	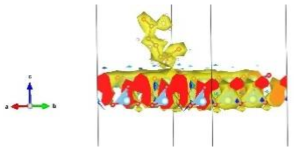	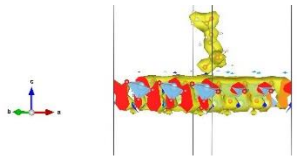
**Orient 7**	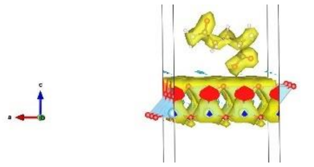	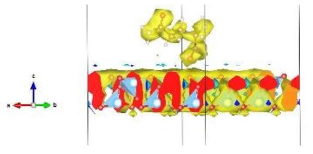	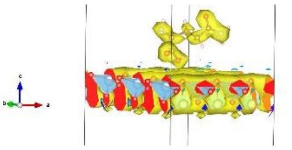	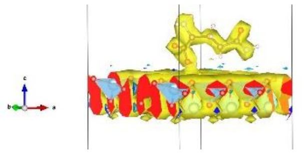
**Orient 9**	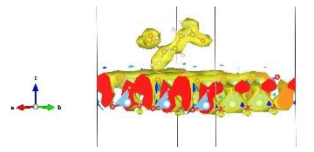	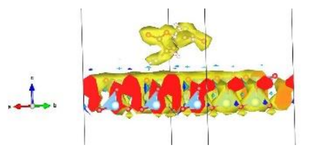	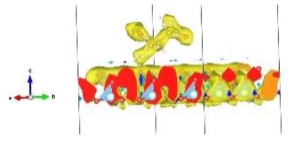	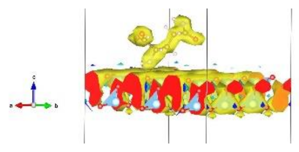

Figure S89. Visualized electron densities of various Glutamine (L) molecule conformations (Orient 3, 4, 6, 7, and 9) on pristine 101 anatase surfaces.

System	Au-doped	Ag-doped	Cu-doped
**Orient 3**	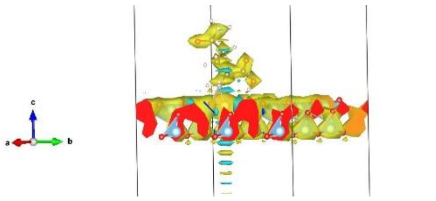	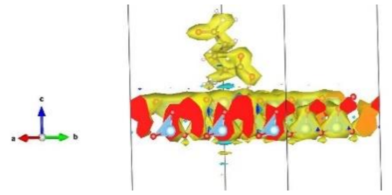	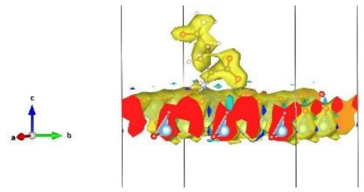
**Orient 4**	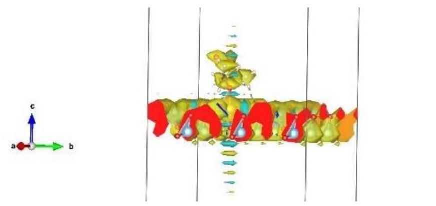	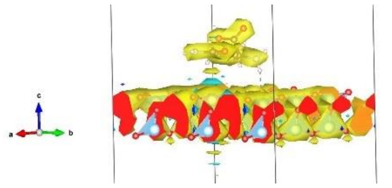	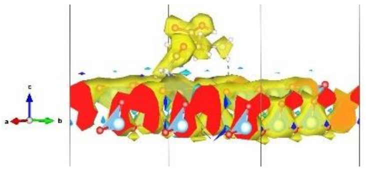
**Orient 6**	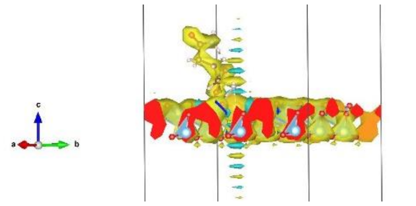	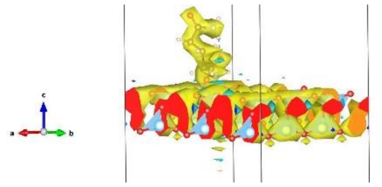	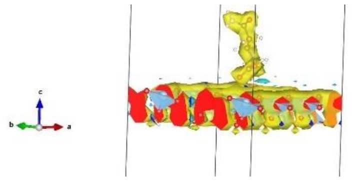
**Orient 7**	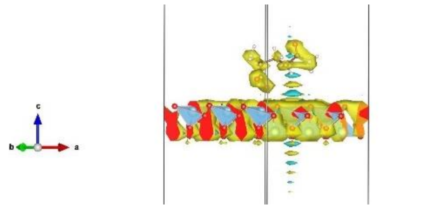	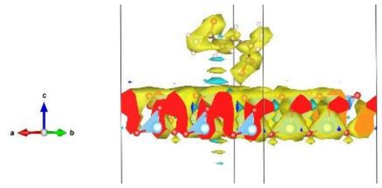	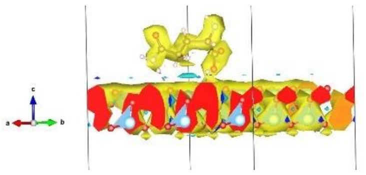
**Orient 9**	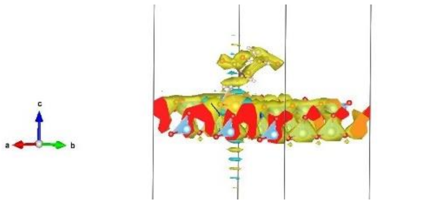	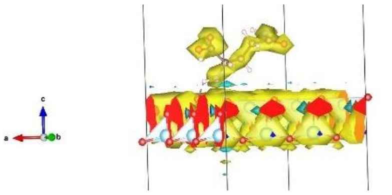	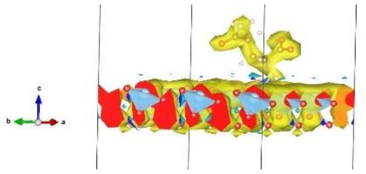

Figure S90. Visualized electron densities of various Glutamine (L) molecule conformations (Orient 3, 4, 6, 7, and 9) on Au / Ag / Cu doped 101 anatase surfaces.

References:

[1] Bensaude-Vincent, B. (1986). Mendeleev's periodic system of chemical elements. The British Journal for the History of Science, 19(1), 3-17.
